# Systematic Mapping of Protein Mutational Space by Prolonged Drift Reveals the Deleterious Effects of Seemingly Neutral Mutations

**DOI:** 10.1371/journal.pcbi.1004421

**Published:** 2015-08-14

**Authors:** Liat Rockah-Shmuel, Ágnes Tóth-Petróczy, Dan S. Tawfik

**Affiliations:** Department of Biological Chemistry, Weizmann Institute of Science, Rehovot, Israel; University College London, UNITED KINGDOM

## Abstract

Systematic mappings of the effects of protein mutations are becoming increasingly popular. Unexpectedly, these experiments often find that proteins are tolerant to most amino acid substitutions, including substitutions in positions that are highly conserved in nature. To obtain a more realistic distribution of the effects of protein mutations, we applied a laboratory drift comprising 17 rounds of random mutagenesis and selection of M.HaeIII, a DNA methyltransferase. During this drift, multiple mutations gradually accumulated. Deep sequencing of the drifted gene ensembles allowed determination of the relative effects of all possible single nucleotide mutations. Despite being averaged across many different genetic backgrounds, about 67% of all nonsynonymous, missense mutations were evidently deleterious, and an additional 16% were likely to be deleterious. In the early generations, the frequency of most deleterious mutations remained high. However, by the 17th generation, their frequency was consistently reduced, and those remaining were accepted alongside compensatory mutations. The tolerance to mutations measured in this laboratory drift correlated with sequence exchanges seen in M.HaeIII’s natural orthologs. The biophysical constraints dictating purging in nature and in this laboratory drift also seemed to overlap. Our experiment therefore provides an improved method for measuring the effects of protein mutations that more closely replicates the natural evolutionary forces, and thereby a more realistic view of the mutational space of proteins.

## Author Summary

Understanding and predicting the effects of single nucleotide polymorphisms (SNPs) is of fundamental importance in many fields. Systematic experimental mappings of the effects of such mutations within a given gene/protein comprise an essential experimental tool for determining protein function and for refining models of protein evolution, as well as an important resource for improving prediction algorithms. Here, we present the results of a laboratory system that mimics the manner by which protein sequences diverge in nature: a prolonged process of gradually accumulating random mutations that retain the protein’s structure and function. The change in frequencies of mutations over generations, as obtained by deep sequencing, enabled us to assess the relative effects of all possible SNPs at the background of an accumulating number of mutations. Compared to previous reports, we found that > 80% of all possible amino acid exchanges have potential deleterious effects, with 67% being clearly deleterious. Tolerance *vs*. purging of mutations in our prolonged drift also showed better correlation with natural diversity. Overall, our experimental setup provides a better understanding of how protein sequences diverge in nature, plus a new basis for improving the prediction accuracy of the effects of protein mutations, and specifically of SNPs.

## Introduction

The ability to reliably measure and predict the effects of amino acid mutations in proteins is of fundamental importance to protein engineering and design and for understanding protein evolution and human genetic variation. Data regarding the effects of individual mutations originate from two major sources: sequence analysis of natural proteins, and laboratory experiments. Phylogenetic analyses enable insights regarding how protein sequences diverge [1, 2] and what dictates the purging of mutations [3, 4]. Protein phylogenies also allow us to predict whether a given mutation might be deleterious or neutral, assuming that the fitness effects of mutations correlate with their occurrence in orthologous sequences (reviewed in [5–7]). The algorithms are relatively accurate in predicting disease-causing mutations [8, 9]. However, many nonsynonymous SNPs predicted to have a deleterious effect are not clearly associated with a disease phenotype [10], either because they are rare [11] or because a deleterious effect in a single gene often results in no phenotype at the organismal level [12]. Indeed, the effects of mutations at the isolated protein and organismal levels do not necessarily overlap. Predictors may also fail in assigning deleterious effects to mutations in highly conserved sites that when mutated experimentally appear to be neutral [13]. Exhaustive datasets listing the effects of all mutations within a given gene/protein, independently of organismal effects, would therefore greatly improve prediction [14]. Systematic experimental mappings of the effects of mutations within one given gene/protein are therefore crucial for understanding protein evolution, as well as an attractive resource for improving predictions [15, 16] and for refining protein design algorithms [17].

Experiments that systematically map the effects of mutations in a given protein are generally conducted through either saturation mutagenesis, using NNS codons to diversify individual sites [18–21], or random mutagenesis along the entire gene using error-prone replication [22]. In both cases, the diversified gene repertoires are subjected to selection that purges deleterious mutations, and then sequenced to identify which mutations are tolerated. Recently, advanced gene synthesis technologies and deep sequencing have yielded exhaustive mappings (for examples see [23–38]; reviewed in [16]). However, although deep mutational scanning provides a powerful means of studying protein structure-function, there remain challenges that are yet to be tackled [16]. Foremost, the relevance of the results of laboratory experimental mappings our understanding of natural protein evolution may be limited.

Specifically, there is a disagreement between the trends indicated by experimental mappings *versus* natural protein diversity. *In silico* analyses of natural protein diversities suggest that the vast majority of mutations are deleterious [39–43]. Given enough drift, mutations at other sites enable the acceptance of certain deleterious substitutions. But at a background of a given sequence, most substitutions would result in the loss of configuration stability and/or function [41, 43–45]. Experimental mappings, however, mostly portray a different picture–the majority of mutations are tolerated (for examples, see [25–27, 33, 36, 46, 47]). Accordingly, a poor correlation between the acceptance of mutations in the laboratory and the occurrence of the same exchanges in natural orthologs of the studied protein has been noted [25–27, 33, 36]. For example, positions that are 75–90% conserved in Hsp90 tolerated a range of amino acids some of which are not seen in any ortholog [25]. However, at lower expression levels, these mutations did reveal deleterious fitness effects, thus indicating that the sensitivity of the experimental system is a key parameter [48].

The comparison of results from different experimental mappings is also problematic. The experiments not only address different proteins, but also apply different mutagenesis strategies and methods of determining the effects of mutations. In some cases the measured effects of mutations relate to growth of the host organism (*e*.*g*., antibiotics resistance) and in others to the biochemical function of the targeted protein in isolation (*e*.*g*. levels of fluorescence, or of DNA methylation, as applied here). Nonetheless, the disagreement between tolerance in the laboratory and occurrence amongst natural sequence raises several questions. Does the absence of a given exchange within natural orthologs indicate its deleterious fitness effect, or does the sparse and sporadic sampling of natural sequences prevents reliable prediction? Do laboratory experimental setups adequately reproduce the constraints that shape protein sequences in nature, or do tolerance or acceptance of a mutation in the laboratory have limited relevance to the evolutionary history/future of a protein. Finally, obtaining a realistic distribution of the fitness effects (DFE) of protein mutations remains a worthy goal [36, 46, 49, 50].

To address the above questions, and obtain a more realistic distribution of fitness effects of protein mutations, we have set up a laboratory system that better mimics the manner by which protein sequences diverge in nature. To this end, we performed 17 iterative rounds of random mutagenesis and purifying selection. This laboratory experiment does not address crucial elements of natural drifts (mutation rates, population sizes, and organismal fitness demands). It does, nonetheless, mimic the process of prolonged accumulation of mutations under purifying selection to maintain the protein’s structural and functional integrity (hence the term ‘neutral drift’). As a model, we used a bacterial DNA methyltransferase, M.HaeIII, which can be readily placed under purifying selection in the laboratory. At different rounds along this prolonged drift, the ensembles of gene variants that survived the purifying selection were subjected to deep sequencing. The naïve, unselected mutational repertoire was similarly sequenced. This enabled us to determine the frequency of occurrence, and hence the relative fitness effects of all single nucleotide mutations in M.HaeIII. As described in the following pages, our results differed from those of other experimental mappings in several key respects.

## Results

### Laboratory drift of M.HaeIII

M.HaeIII is a DNA methyltransferase isolated from *Haemophilus aegyptius*. Being part of the bacterial restriction-modification system, this enzyme selectively methylates GGCC DNA sequences, and thereby protects DNA from digestion by the cognate endonuclease, HaeIII. Sequence specific methylation-restriction offers a facile way of performing laboratory evolution. As described in earlier works [51, 52] (**S1 Fig**), M.HaeIII's open reading frame was randomly mutated using PCR with an error-prone polymerase, cloned into an expression plasmid and transformed to *E*. *coli*. In each bacterium, the encoding plasmid is methylated, or not, depending on whether the M.HaeIII gene variant it encodes is properly folded and functional. Following ‘expression’ of the plasmid encoded M.HaeIII variants in individual bacteria, the plasmids were pooled and digested with HaeIII, thus selecting for M.HaeIII’s native specificity [51, 52]).

The starting point for these experiments was a variant of M.HaeIII optimized for soluble and functional expression in *E*. *coli*. This variant carried four consensus substitutions replacing the amino acid in M.HaeIII with the one that dominates in all M.HaeIII’s orthologs [50]. For the sake of simplicity we refer to this variant as *wild-type M*.*HaeIII*. These consensus substitutions are likely to have a stabilizing, compensatory effect, and spontaneously accumulate in accelerated, laboratory drifts. They may thus allow a larger variety of deleterious mutations to be accepted, especially during the first rounds of mutagenesis [53].

In *Haemophilus aegyptius*, M.HaeIII is under a strong and constitutive selection pressure imposed by the presence of the cognate restriction enzyme HaeIII–a DNase that would cause chromosomal breaks unless the genome is methylated at all HaeIII sites. The HaeIII restriction-modification system is naturally encoded by single copy chromosomal genes [54, 55]. In our experimental system, M.HaeIII was encoded by a multi-copy plasmid (~400 copies per cell). To avoid unrealistic enzyme doses, expression was driven from a tightly controlled promoter with no induction. Although M.HaeIII’s levels in *Haemophilus aegyptius* are unknown, its expression level in the *E*. *coli* cells of our experimental setup is extremely low (a similar plasmid showed no detectible GFP signal when inducer levels were ≤20 μg/ml [56], and we used no inducer). This basal expression level was nonetheless sufficient to enable wild-type M.HaeIII to methylate all GGCC sites, not only in the encoding plasmid, but also within the *E*. *coli* host's chromosome, as is the case with natural methyltransferases [51].

M.HaeIII underwent 17 rounds of random mutagenesis, at an average mutational rate of 2.2±1.6 nucleotide mutations per gene per generation followed by purifying selection (*i*.*e*., digestion of the encoding plasmids with HaeIII nuclease). To avoid false positives due to mutations in GGCC sites, the applied M.HaeIII’s coding sequence (ORF) contained no GGCC sites whist the encoding plasmid contained 14 such sites including three sites within the antibiotic selection marker. Each round, selection was repeated three times (*i*.*e*., repeated isolation of plasmid DNA pool from the grown bacteria, digestion with HaeIII, and transformation into *E*. *coli*). Subsequently, the drifted M.HaeIII’s ORF was mutagenized and recloned into a fresh plasmid for the next round of selection. We ensured the same level of selection pressure and the absence of bottlenecks throughout: ≥10^5^ independent transformants were passed to the next round (effective population size, *N*_*e*_ > 10^5^). The drifting M.HaeIII thus met the conditions that essentially eliminate the possibility of mutations fixing by chance (1/*N*_*e*_ <10^−5^). Mutations that were enriched are therefore likely to have provided a selective advantage, most typically, as shown below, a compensatory effect.

By the 17^th^ round, the drifted genes carried on average of 18±1.6 mutations per gene in total, and 9.6±0.7 nonsynonymous mutations per gene (determined in parallel by deep sequencing and conventional Sanger sequencing of the full length ORFs of randomly chosen genes; see *‘Dynamics of the laboratory drift‘* below).

### Mapping M.HaeIII’s mutational space

The mutational spectrum of the unselected, naïve gene pool (dubbed G0), and of the pools after three (G3), seven (G7) and seventeen (G17) rounds of selection, were analyzed by Illumina high-throughput sequencing. Mutations were identified using a script that aligned all codon triplets to the reference gene, wild-type M.HaeIII (**S1 File**). The background frequency that stems from the Illumina sequencing PCRs and sequencing errors was determined using the sequencing data for the region upstream of the randomly mutated ORF of M.HaeIII (the N-terminal fused His tag that was part of the encoding vector and was hence not subjected to mutagenesis). In this manner, the frequencies of all single, double and triple nucleotide mutations were determined by the fraction of sequence contigs that carry a mutation out of all contigs that covered the respective position. The amino acid mutational frequencies were subsequently determined by summing up the frequencies of all codon triplets that yield a given amino acid (See **S1, S2**and **S3 Files**).

In theory, M.HaeIII’s sequence space includes 6,580 possible amino acid mutations; *i*.*e*., 329 positions, each mutated to all other 19 different amino acids or to a stop codon (**Table 1**). However, the immediate mutational space originates from single nucleotide mutations. Subsequent nucleotide mutations within the same codon were found (dubbed double and triple mutations; **Table 1**), but at very low frequencies (in G17—an average of 0.052% and 0.003% of nonsynonymous double and triple mutations). However, these double and triple mutations only appeared at later stages, and after many other positions had changed due to single nucleotide mutations. Single nucleotide missense mutations also dominate polymorphism, and thus, our analysis focused on their effects. We thus examined all 1,957 possible missense single point mutations, namely all amino acid exchanges accessible by single nucleotide mutations; **Table 1**). The effects of stop codons were also examined as described in *‘Tolerance of nonsense mutations’*.

**Table 1:** The theoretically possible *vs*. observed mutational space of M.HaeIII.

	Nonsynonymous						Synonymous Mutations		
	Missense mutations			Nonsense Mutations (Stop codons)					
	1 nt	2 nt	3 nt	1 nt	2 nt	3 nt	1 nt	2 nt	3 nt
**All possible mutations**	**1,957**	3,190	1,104	125	145	59	321	55	18
**Before selection:**	** **			** **			** **		
Observed G0	1,880*	8	0	125	0	0	321	0	0
**After selection**:									
Observed G3	1,401	26	0	33	0	0	321	1	0
Observed G7	1,302	41	0	24	0	0	320	5	0
Observed G17	1,374	228	1	11	1	0	320	14	0
**Total observed after selection**	1,541	275	1	36	1	0	321	16	0
**Total observed (including G0)**	1,915	281	1	125	1	0	321	16	0
**Coverage (drift)**	100%	8.8%	0.1%	100%	0.7%	0.0%	100%	29.1%	0.0%

The number of 'all possible mutations’ is the number of all possible mutations derived from the DNA sequence of wild-type M.HaeIII (329 codons), either nonsynonymous mutations (missense or nonsense) or synonymous mutations. The number of 'observed' mutations comprises the sum of all the mutations identified with above background frequencies in each library. 'Coverage' relates to the percentage of the total observed mutations out of all possible mutations.

* 1,880 mutations were observed at G0 with ≥0 ‘net’ frequencies, and 77 mutations were observed at lower than background frequencies. Out of these, 35 were detected in G3, G7 and/or G17. The remaining 42 mutations were also observed with under background frequencies in G3, G7 and G17, and were assigned a ‘net’ frequency of 0 (*i*.*e*., as eliminated by selection, marked in red in **S3 File**).

‘1/2/3 nt’–all mutations accessible through single/double/triple nucleotide substitutions of a given codon.

All possible single nucleotide mutations were detected in the unselected G0 library at the raw data level– 329X9 = 2,961 possible single nucleotide mutated codons, that in turn yield 1,957 possible single nucleotide amino acid mutations (raw data provided as S2 File). However, 77 mutations at G0 were observed at lower than background frequencies. That a mutation is observed under what we defined as the background frequency is not necessarily an indication that it did not occur. The background frequency was derived from averaging the frequencies for relatively few positions compared to the measured ones (20 positions versus 329) and thus it is conceivable that a small fraction of the latter (<1%) will deviate from this average. Indeed, out of the 77 mutations, 35 were detected in the later, selected rounds, and some were even enriched. The remaining 42 mutations were also observed with under background frequencies in later rounds. We therefore assume that they were strongly purged and assigned them as eliminated mutations. Overall, our analysis related to the complete set of 1,957 single nucleotide missense mutations with >98% (1,915/1,957) of these being covered with complete confidence.

### The spectrum and rate of mutagenesis

The spectrum of mutations covered by our experiment was dictated by the genetic code, M.HaeIII’s DNA sequence, and by the nucleotide substitution matrix that underlined our mutagenesis protocol. Although we used an engineered, error-prone DNA polymerase, the obtained spectrum of mutations was similar to that naturally observed in *E*. *coli*. Specifically, a transition/transversion ratio of ~1.3 was observed in our naïve repertoire (G0) similar to what has been observed in the comparison of closely related *E*. *coli* genomes (0.91 or 1.3, [57, 58], **S1 Table**).

The variability in mutation frequencies along M.HaeIII positions in the unselected G0 library was relatively high (1.07 ± 0.24% mutations/position). Thus, mutation frequencies varied not only by the type of base substitution (*e*.*g*. transitions, transversion; **S2A Fig**), but also according to the position of the mutated base along M.HaeIII’s gene. To verify that this variability is not the outcome of limited sampling in G0 (the naïve repertoire that underwent only one round of mutagenesis) we compared the frequencies of synonymous mutations in the unselected library, G0, and in the selected one, G3. As expected, synonymous mutations were under relatively weak selection (detailed below) and thus their frequencies, certainly within the early rounds, largely reflect the rate of mutagenesis. Indeed, the frequencies of synonymous mutations in G0 and in G3 were highly correlated (R = 0.9, **S2B** and **S2E Fig**). By G17, the correlation was still significant although weaker indicating some degree of selection on synonymous mutations (R = 0.6; **S2C Fig**).

The observed frequencies in the unselected library, *f*(G_0_), therefore appear to provide a reliable measure for the positional rates of occurrence of mutations in all 17 mutagenesis steps of the drift. However, for the 77 mutations (out of 1,957) with lower than background frequencies in G0 (**Table 1**), the rate of the occurrence, *f*(G_0_), was based on the base substitution table derived from G0 (**S2A Fig**).

### The relative fitness effects of mutations

Mutations were retained, purged or enriched in each round of our experiment. The change in frequency along the drift therefore reflects the effects of selection per each mutation or, as defined here, their *relative fitness effects* (*W*_*rel*_). The frequency of a given mutation in a given round (*f*(G_n_)) is dictated by its relative fitness (*W*_*rel*_), and relates to the frequency of this mutation in the previous round, *f*(G_n-1_) plus the frequency of re-occurrence at round *n*. For example, the frequencies of neutral mutations (*W*_*rel*_ = 1) are essentially equal to their cumulative rate of occurrence (*f*(G_n_) ~ n *f*(G_0_)). Conversely, the frequencies of deleterious mutations (*W*_*rel*_ < 1) decrease from round to another, in an exponential manner, and their observed frequency is lower than expected from their rate of occurrence (*f*(G_n_) < n *f*(G_0_)). The opposite applies for beneficial mutations (*W*_*rel*_ > 1).

However, since the genes in our drifting ensembles contained multiple mutations, and the applied sequencing approach does not reveal the specific mutational composition of individual genes, the *W*_*rel*_ values measured here relate to the effect of a given mutation at the background of many different genetic compositions. For better and for worse, the measured *W*_*rel*_ values therefore represent an average that ignores epistatic interactions between mutations. This averaging has obvious drawbacks, and may cause biases due to hitchhiking and clonal interference (*e*.*g*. a highly deleterious mutation would result in every other mutation on the same gene having a low *W*_*rel*_) However, under our experimental setup, new mutations are reintroduced in each round of mutagenesis, allowing multiple resampling of the effects of each given mutation at the background of many different mutations, and thus reducing the probability of hitchhiking. Indeed, as indicated below, there are clear indications that hitchhiking and clonal interference did not bias the observed *W*_*rel*_ values. We also note that, in general, allele frequencies, and thereby fitness effects of mutations, are measured in populations comprising individuals with different genetic backgrounds with certain caveats [59–61]. Foremost, the number of sequenced alleles needs to be in the order of thousands [59]–a demand that is amply met in our experiment. Thus, if a mutation is on average purged (W_rel_ <<1), we can conclude that it has deleterious effects on M.HaeIII’s structure and/or function independently of the specific genetic background.

We used the following model to derive the *relative fitness effect*, *W*_*rel*_, from the mutational frequencies observed in the selected *versus* unselected libraries (see Methods for details).

Following the first round:

$$f(G_{1})=f(G_{0})\cdot W_{rel},$$

For the subsequent rounds, the observed mutational frequency, *f*(G_n_), is derived from the mutations inherited from the previous round *f*(G_n-1_) *plus* the mutations newly incorporated in this round. The latter corresponds to the frequency of this mutation in the naïve, unselected ensemble, *f*(G_0_), as discussed above:

$$f(G_{n})=\left[f\left(G_{n-1}\right)+f(G_{0})\right]\cdot W_{rel}$$ (1)

Eq (1) corresponds to a geometrical series that has no closed solution. Thus, to derive the *W*_*rel*_ values of each mutation, we calculated the expected frequency ratio ($\frac{f(G_{n})}{f(G_{0})}$) for a series of discrete *W*_*rel*_ values from absolutely deleterious (*W*_*rel*_ = 0) to highly beneficial (*W*_*rel*_ = 3.5; see Methods and **S3 Fig**). In this manner, each of the 1,957 amino acid mutations measured by the deep sequencing (each derived from the respective single nucleotide mutation; **Table 1**), were assigned a *W*_*rel*_ value.

**Fig 1:**
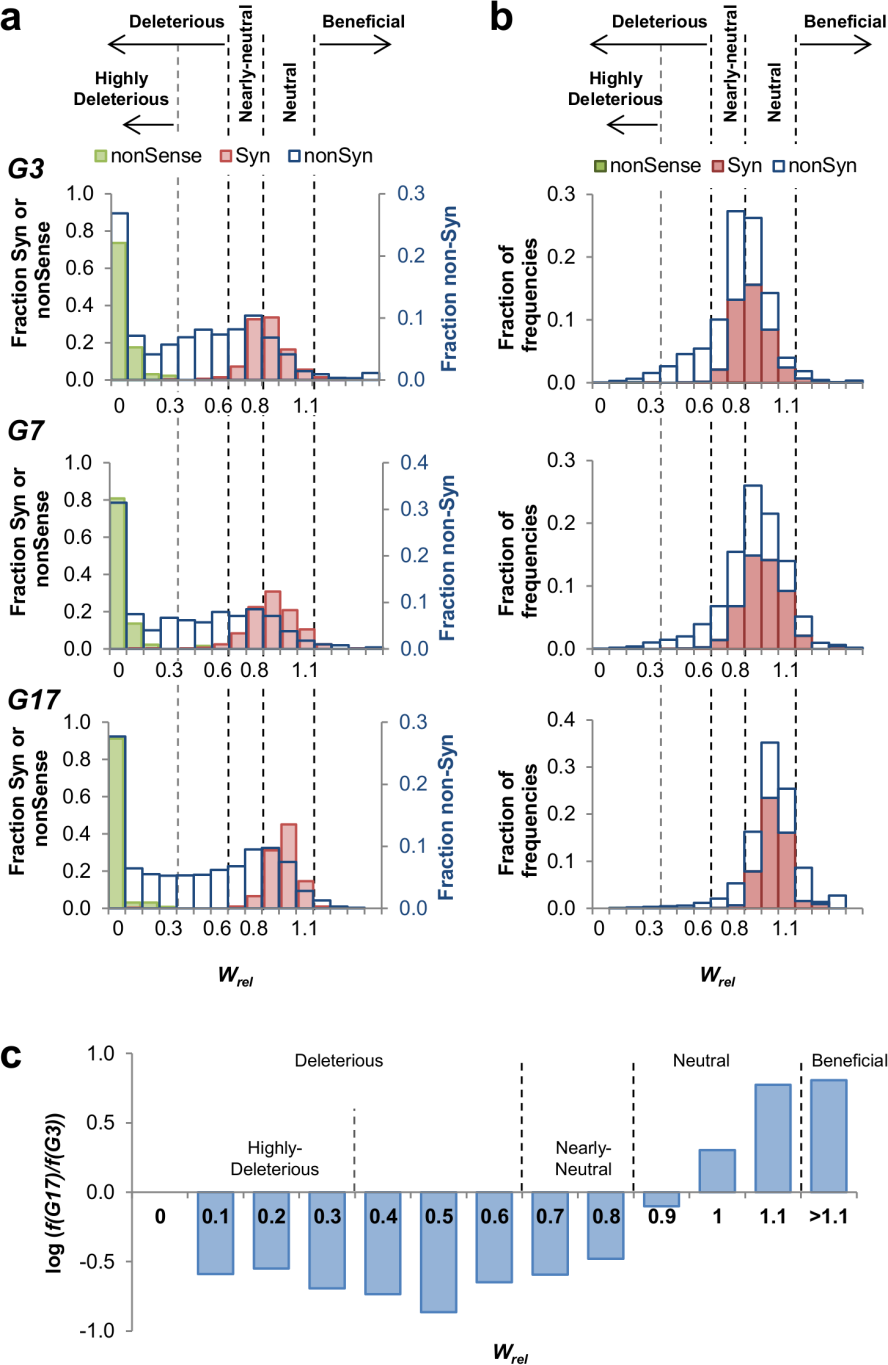
The distribution of fitness effects of mutations in M.HaeIII's drift. **a**. The distributions of fitness effects of all mutations observed in the sequenced rounds of the drift (G3, G7 and G17) by their relative fitness values, *W*_*rel*_. Mutations were binned by unit interval values of *W*_*rel*_ = 0.1, ranging from *W*_*rel*_ = 0 to > 1.4 (missense: n = 1,957; nonsense: n = 125; synonymous: n = 321). **b**. Distribution of the frequencies of mutations within each given range of *W*_*rel*_ values. The frequencies of all mutations within a given *W*_*rel*_ range were summed up and divided by the sum of frequencies for all mutations within the same round. **c**. Log-values of the fold changes in the frequencies per each *W*_*rel*_ range in G17 relative to G3.

We used the variability in the relative fitness effects of synonymous mutations and nonsense mutations to categorize the effects of nonsynonymous mutations [36]. The distribution of synonymous mutations was consistent with their low impact on fitness relative to nonsynonymous mutations (**Fig 1A, ‘Syn’**). The average *W*_*rel*_ value, and standard deviation, for synonymous mutations were found to be 0.82±0.12 for G3, 0.84±0.15 for G7, and 0.91±0.1 for G17 (**Table 2**). Given our hypothesis that other works overestimated the tolerance of mutations, we preferred to under- rather than over-estimate the fraction of deleterious mutations. Accordingly, for the assignment of a deleterious fitness effect, we chose a conservative threshold of two standard deviations under the mean of the relative fitness effect of synonymous mutations ($\bar{X}-2SD$). The $\bar{X}-2SD$ values obtained were 0.58, 0.55 and 0.72 for G3, G7 and G17, respectively (**Table 2**) yielding an average of 0.62 for all 3 ensembles. We therefore used *W*_*rel*_ ≤ 0.6 as the threshold for indicating purging and consequently a deleterious fitness effect of a mutation. However, since, as detailed below, mutations with *W*_*rel*_ values of 0.6–0.8 were found to be systematically purged as the drift progressed, suggesting that in effect they are not neutral. This *W*_*rel*_ range was therefore classified as ‘nearly-neutral’ (**Fig 1A**).

**Table 2:** Distributions of the relative fitness effect values (*W*_*rel*_) for all possible single nucleotide mutations along M.HaeIII gene.

	*G3*			*G7*			*G17*		
	*nonSense*	*Syn*	*nonSyn*	*nonSense*	*Syn*	*nonSyn*	*nonSense*	*Syn*	*nonSyn*
counts (n)	*125*	*321*	*1*,*957*	*125*	*321*	*1*,*957*	*125*	*321*	*1*,*957*
$\bar{X}$ (*W*_*rel*_)	0.042	0.82	0.40	0.028	0.84	0.36	0.020	0.91	0.41
Standard Deviation	0.15	0.12	0.38	0.12	0.15	0.36	0.11	0.10	0.38
$\bar{X}-2SD$		0.58			0.55			0.72	
$\bar{X}-SD$		0.70			0.70			0.82	
$\bar{X}+2SD$	0.34	1.06		0.27	1.13		0.24	1.11	
Fraction of mutations (as in **Fig 1A**)									
Deleterious (*W*_*rel*_ ≤0.6)	96.8%	2.5%	67.5%	98.4%	4.0%	70.3%	98.4%	0.3%	63.1%
Neutral (0.6<*W*_*rel*_≤1.1)	3.2%	95.6%	29.8%	1.6%	93.1%	27.6%	1.6%	98.4%	35.3%
Beneficial (*W*_*rel*_>1.1)	0.0%	1.9%	2.7%	0.0%	2.8%	2.1%	0.0%	1.2%	1.7%
Fraction of the mutations by their frequencies (as in **Fig 1B**)									
Deleterious (*W*_*rel*_≤0.6)	0.10%	0.2%	15.0%	0.05%	0.3%	8.7%	0.01%	0.0%	2.9%
Neutral (0.6<*W*_*rel*_≤1.1)	0.13%	41.7%	40.1%	0.04%	46.4%	37.5%	0.03%	48.0%	36.3%
Beneficial (*W*_*rel*_>1.1)	0.00%	0.8%	2.0%	0.00%	2.6%	4.4%	0.00%	2.5%	10.3%
Total fraction	0.23%	42.7%	57.0%	0.09%	49.3%	50.6%	0.04%	50.5%	49.4%
*N* per position	0.003%	0.53%	0.71%	0.002%	1.11%	1.14%	0.002%	2.97%	2.91%

‘nonSense’—refers to the all possible stop codons that can be derived by single nucleotide mutations from the reference gene.

‘Syn’–refers to all the possible synonymous mutations giving the same amino acid as found in the reference gene and can be derived by single nucleotide mutations. Note that 8 positions in the reference gene with Met and Trp that are encoded by one codon only were excluded.

‘nonSyn’–refers to all the possible nonsynonymous, missense mutations that can be derived by single nucleotide mutations from the reference gene.

‘$\bar{X}$ (*W*_*rel*_)’–refers to the relative average *W*_*rel*_ value for all possible single nucleotide mutations.

‘$\bar{X}-2SD$ ‘ for the *synonymous mutations* (*W*_*rel*_ ≈ 0.6, on average) was set as the upper threshold for ‘deleterious’ mutations.

‘$\bar{X}-SD$ ‘ for the *synonymous mutations* was used as sub-category of ‘neutral’ mutations, categorizing mutation with *W*_*rel*_ values in the range of 0.6–0.8 as ‘nearly-neutral’.

The ‘$\bar{X}+2SD$ ‘ of the *synonymous mutations* (~1.1 on average) was set as the upper threshold of neutral mutations, thus categorizing mutations with *W*_*rel*_ >1.1 as ‘beneficial’.

The ‘$\bar{X}+2SD$ ‘ of the *nonsense mutations* (*W*_*rel*_ ≈ 0.3, on average) was set as the upper threshold defining ‘*highly-deleterious*’ mutations.

‘Neutral’, ‘Deleterious’ and ‘Beneficial’ show the fractions of mutations found within the defined thresholds of *W*_*rel*_ values for each category.

‘*N* per position’ is the average mutational frequency per position observed in each library for the cited type of mutation (nonsense, synonymous or nonsynonymous).

The ‘Fraction’ is the fraction of the cited type of mutation out of all mutations observed in a given round.

The $\bar{X}+2SD$ threshold was similarly applied for categorizing beneficial mutations, thus stetting the threshold for beneficial mutations as *W*_*rel*_ > 1.1. Within this threshold, only 4 out of 321 synonymous mutations were defined as beneficial relative to 33 nonsynonymous mutations. Indeed, within the 0.6–1.1 *W*_*rel*_ range defined here as neutral, >93% of the synonymous mutations observed in the three selected ensembles (G3, G7, G17) were assigned as neutral (**Table 2**). The potential deleterious or beneficial effects of the remaining 7% were not analyzed here. The selection acting on synonymous mutations may, amongst other factors, relate to different codon usage in *E*. *coli*. Overall, given the applied thresholds, the likelihood of misassignment of neutral mutations as deleterious or beneficial was < 4% (**Table 2**).

The *W*_*rel*_ threshold for defining ‘highly deleterious’ mutations was derived from the distributions of nonsense mutations that are, beyond doubt, deleterious (see also *‘Tolerance of nonsense mutations‘ below)*. The average *W*_*rel*_ value, and standard deviation, for nonsense mutations were found to be 0.042±0.15 for G3, 0.028±0.13 for G7, and 0.020±0.11 for G17 (**Table 2**). Thus, a threshold of $\bar{X}+2SD$, *i*.*e*., *W*_*rel*_ ≤ 0.3, was chosen for categorizing highly deleterious mutations.

In summary, nonsynonymous mutations were categorized as ‘Deleterious’ if their *W*_*rel*_ values were ≤ 0.6, and ‘Highly deleterious’ if *W*_*rel*_ ≤ 0.3 (including eliminated mutations, *W*_*rel*_ = 0, *i*.*e*., when the net frequency of a mutation was zero). Mutations were assigned as ‘Nearly-neutral’ if their frequencies in the selected populations were in the range of *W*_*rel*_ = 0.6–0.8 ($\bar{X}-2SD$ of the distribution of synonymous mutations) and ‘Neutral’ in the range of *W*_*rel*_ = 0.8–1.1. Finally, enrichment in the selected repertoires (*W*_*rel*_ >1.1, $\bar{X}+2SD$ of the distribution of synonymous mutations) indicated a ‘Beneficial’ fitness effect.

### The distribution of fitness effects of mutations

As can be seen in **Fig 1A**, the distribution of relative fitness effects of synonymous mutations centered near neutrality (*W*_*rel*_ ~1; see **Table 2**for mean and standard deviation). In contrast, the distribution of the nonsynonymous mutations encompasses primarily deleterious mutations. Overall, ~67% out of all the possible nonsynonymous single nucleotide mutations (~1,310/1,957) were found to be deleterious, even within the conservative threshold of *W*_*rel*_ of ≤ 0.6 (**Table 2**, ‘**nonSyn**’). Removal of the 42 mutations that were observed below background frequency in all repertoires, and assigned as eliminated, has almost no impact on the fraction (~1,268/1,915 = ~66%). Note that **Fig 1A** shows the derived fitness effects of all possible single nucleotide mutations regardless of their frequencies in the drifting populations. Indeed, a similar fraction was assigned as deleterious in all the three selected libraries (**Fig 1A,** ‘**nonSyn**’). The effect that selection had on purging deleterious mutations is clearly seen in the distribution of their frequencies (**Fig 1B**). This distribution shifted during the drift: from ~15% of all mutations denoted as deleterious in G3, to only ~3% in G17 (**Fig 1C**). Further, by our conservatively chosen threshold, mutations with assigned *W*_*rel*_ from 0.6 to 0.8 were considered as ‘Nearly-neutral’. However, mutations within this *W*_*rel*_ range were systematically purged throughout the drift, from ~22% in G3 to ~7% in G17 (**Fig 1C**), indicating small yet consistent deleterious effects. Further, as discussed below, these mutations were accepted at the background of beneficial mutations, most likely owing to their compensatory effect (as discussed in the section below). If all mutations with *W*_*rel*_ ≤ 0.8 are considered, then ~83% of all possible mutations in M.HaeIII have a deleterious effect. In agreement with the reduction in the frequency of deleterious mutations, the total frequency of beneficial mutations (*W*_*rel*_ > 1.1) increased consistently, from ~2% in G3 to ~10% in G17.

Consistent with the distribution of *W*_*rel*_ values being the same along the drift (**Fig 1A**), we also observed that the *W*_*rel*_ values per given mutation remain largely the same along the drift, *i*.*e*., when derived from the sequenced frequencies in G3, G7 or G17 (**S3B** and **S3C Fig**). This was despite the fact that the average number of mutations per gene increased form 2.4 in G3 to 9.6 in G17. It therefore seems that hitchhiking and/or clonal interference did not significantly bias our data, and that the derived *W*_*rel*_ values that average the effect of a given mutation over different genetic background are relevant.

### Dynamics of the laboratory drift

The discrepancy between our results and the results of other experimental mappings is likely due to differences between measuring the effect of a single, or at most a few mutations, to measuring the effect of accumulated mutations over a long mutational drift. To support this hypothesis, we examined the rate of accumulation of mutations along the various rounds of the drift. To this end, in addition to deep-sequencing of the gene ensembles of 3 rounds (G3, G17 and G17), we also performed conventional Sanger sequencing of the full length ORFs of randomly picked variants from each round.

**Fig 2:**
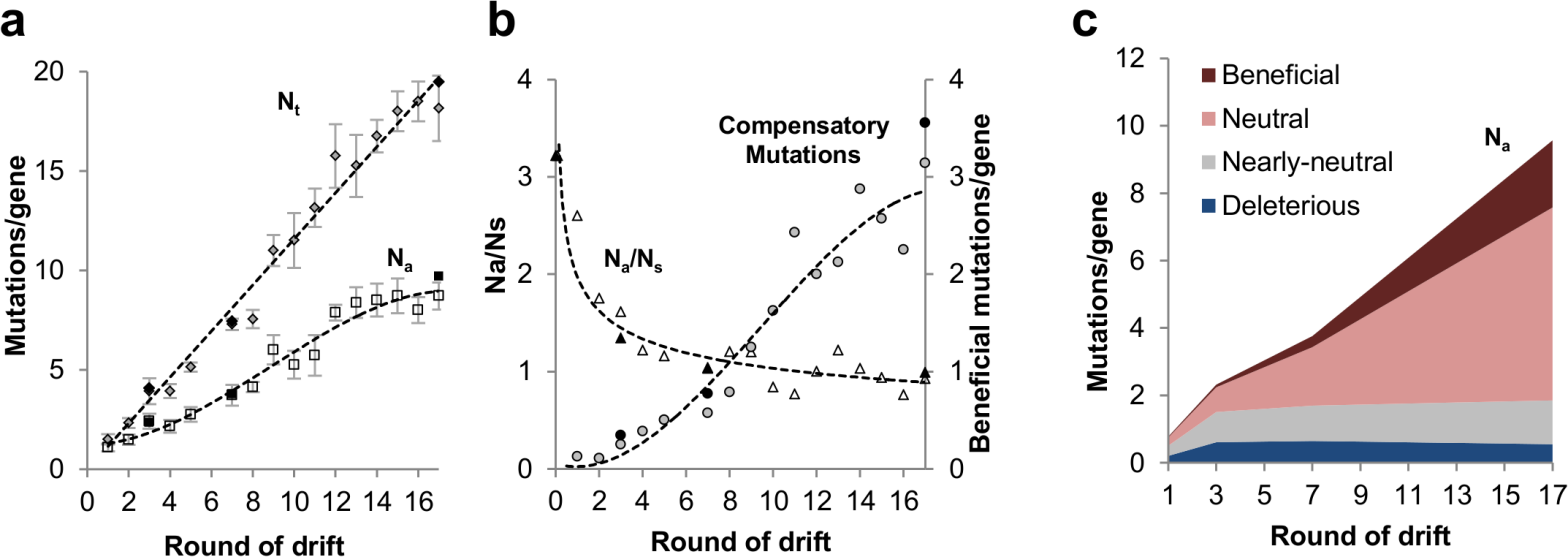
Dynamics of the laboratory drift. **a**. Cumulative mutational loads (average number of mutations per gene) along the 17 rounds of the laboratory neutral drift. *N*_*t*_ is the average number of total mutations per gene (shown as ‘diamonds), *N*_*a*_ is the average number of nonsynonymous mutations per gene (shown as ‘squares’). Mutational loads were derived from deep-sequencing of G0, G3, G7 and G17 repertoires (full points) as well as by Sanger sequencing—standard, full-length sequencing of randomly selected variants from each round (empty points). Error bars show the standard error for the calculated averages. The lines illustrate the observed trends (not a fit for a specific equation). **b.** *N*_*a*_ */N*_*s*_ is ratios of nonsynonymous to synonymous mutations (shown as ‘triangles); and the average number of compensatory mutations per gene (*W*_*rel*_ >1.1, shown as ‘circles’). Compensatory mutations are listed in **S2 Table** and were defined as enriched mutations, either by assigned beneficial fitness effect for individual mutations by (*W*_*rel*_ >1.1) or high positional fitness effect (the averaged *W*_*rel*_ per position as calculated in **Fig 3A**, *W*_rel (Positional)_ >1.1). The effect of compensatory mutations discussed in the section of “*Dynamics of the laboratory drift*”. **c.** The cumulative mutational load for mutations with different fitness effects: ‘deleterious’ (*W*_*rel*_ ≤0.6), ‘Nearly-neutral’ (*W*_*rel*_ 0.61–0.8), ‘Neutral’ (*W*_*rel*_ 0.81–1.1) and ‘Beneficial’ (*W*_*rel*_ >1.1).

As expected, the total number of mutations per gene (*N*_*t*_) increased linearly throughout the drift, certainly up to the 15^th^ round, at the average of 1.16 mutations/gene/round (**Fig 2A**). However, as the drift progressed, the intensity of purged nonsynonymous mutations increased as indicated by the ratio of nonsynonymous to synonymous mutations (*N*_*a*_ */N*_*s*_; whereby *N*_*a*_ denotes the average frequency of nonsynonymous mutations and *N*_*s*_ the average frequency of synonymous mutations; **Fig 2B**). Specifically, the first round (G1) exhibited a *N*_*a*_ */N*_*s*_ ratio of 2.6, only mildly lower than 3.2 –the ratio in G0, the unselected repertoire. However, by the 5^th^ round, the *N*_*a*_ */N*_*s*_ ratio dropped to a value of ~1. By the 14^th^ round, the accumulation of nonsynonymous mutations (*N*_*a*_) had slowed down, in addition to a slowdown in the total accumulation of mutations (*N*_*t*_; **Fig 2A**).

That the tolerance to mutations decreased as the drift progressed is also reflected in the continuous decline in frequency of deleterious mutations along the drift. About a third of all possible mutations were eliminated by the 3^rd^ round (*W*_*rel*_ = 0, ‘Eliminated’) whereas deleterious mutations (*W*_*rel*_ = 0.01–0.6) were observed in the drifting ensembles throughout the 17 rounds. However, their frequency was small and remained constant throughout (~0.6 deleterious mutations/gene; **Fig 2C**). Thus, as mutations gradually accumulated, the relative frequency of deleterious mutations became increasingly lower–in G17 their fraction became 0.06 (0.6 out of the ~9.6 nonsynonymous mutations/gene) relative to 0.26 in G3 (0.6 out of the ~2.4 nonsynonymous mutations/gene). In effect, the majority of mutations that did accumulate beyond G3 were neutral (**Figs 1B** and **2C**). This finding also indicates that hitchhiking and/or clonal interference does not significantly bias our data.

In addition to the accumulation of the neutral mutations (*W*_*rel*_ = 0.8–1.1) beyond G3, the later rounds were accompanied by the enrichment of beneficial mutations (**Fig 1C**). The beneficial mutations (*W*_*rel*_ > 1.1) are likely to be compensatory mutations, increasing the global stability of M.HaeIII, or locally interacting with a specific deleterious mutation. The applied sequencing method does not reveal the specific mutational composition of individual genes, and thus, there is no way of detecting enriched, beneficial mutations that have a specific, local compensatory effect. However, as previously shown [53], mutations that were enriched in a prolonged neutral drift were experimentally confirmed to have global, stabilizing effects that compensate for a wide range of deleterious destabilizing mutations. The global compensatory effect can also be deduced from the identification of most enriched mutations as consensus mutations (**S2 Table**; see also Ref. [53]). Further, under selection for the acquisition of five different new DNA target specificities [51], the same mutations were rapidly fixed in all the evolved lines irrespective of which new specificity was selected (**S2 Table**). Compensatory mutations are essential for the acquisition of new functions because mutations that confer new functions tend to severely undermine protein stability [62–64].

By G17, each gene carried, on average, 1.99 beneficial mutations relative to 0.08 in G3 (**Fig 2C**). Conversely, the fraction of enriched, beneficial mutations in the drifting genes (out of all nonsynonymous mutations) became 0.21 in G17 relative to 0.03 in G3 (**Fig 1B and 1C**). Thus, not only was the mutational tolerance limited beyond G3, but also, the acceptance of nearly-neutral mutations (*W*_*rel*_ = 0.61–0.8, **Fig 1**) was dependent on the co-accumulation of compensatory mutations.

### Tolerance of nonsense mutations

The ability to tolerate highly deleterious mutations at the onset of the drift, but not once mutations further accumulate, is also vividly exemplified by the tolerance of nonsense mutations–mutations leading to stop codons (**S4 Fig**) or frameshifing insertions/deletions (InDels). The occurrence and tolerance of InDels in the selected G17 M.HaeIII library has been described [52], indicating that certain nonsense mutations were tolerated to some degree due to translational slippage that results in a correctly translated protein despite a frame-shifted gene. However, the levels of full length, functional proteins translated from frame-shifted genes is obviously much lower than for wild-type, in some cases as little as 1% [52].

The second form of nonsense mutations are stop codons. At the onset of the drift, at least one stop codon mutation, in position 176, was moderately tolerated (*W*_*rel*_ in G3 = 0.74). Stop codons in other positions were also found, although with lower *W*_*rel*_ values (**S4A Fig**). However, once other mutations that reduce protein dose and/or function accumulated, nonsense mutations were almost entirely purged (*e*.*g*. *W*_*rel*_ for position 176 in G7 = 0.47, and in G17 = 0.24). By the later rounds, stop codons were found almost only after position 324– a region that is not under functional selection (**S4 Fig**). The stronger purging effect of nonsense mutations in later rounds was also manifested in an increasing fraction of nonsense mutations being assigned a *W*_*rel*_ value of 0 (**Figs 1, ‘nonSense’**, and **S4B**).

### Correlation with the diversity in natural orthologs

Several laboratory mutational tolerance experiments indicated the acceptance of mutations in positions that are highly conserved in natural orthologs [21, 25–27]. We therefore examined to what degree M.HaeIII's orthologs predict acceptance in our experiment; namely, do the measured relative fitness effects of mutations (*W*_*rel*_) correlate with the degree of divergence of the corresponding position in M.HaeIII’s natural orthologs?

**Fig 3:**
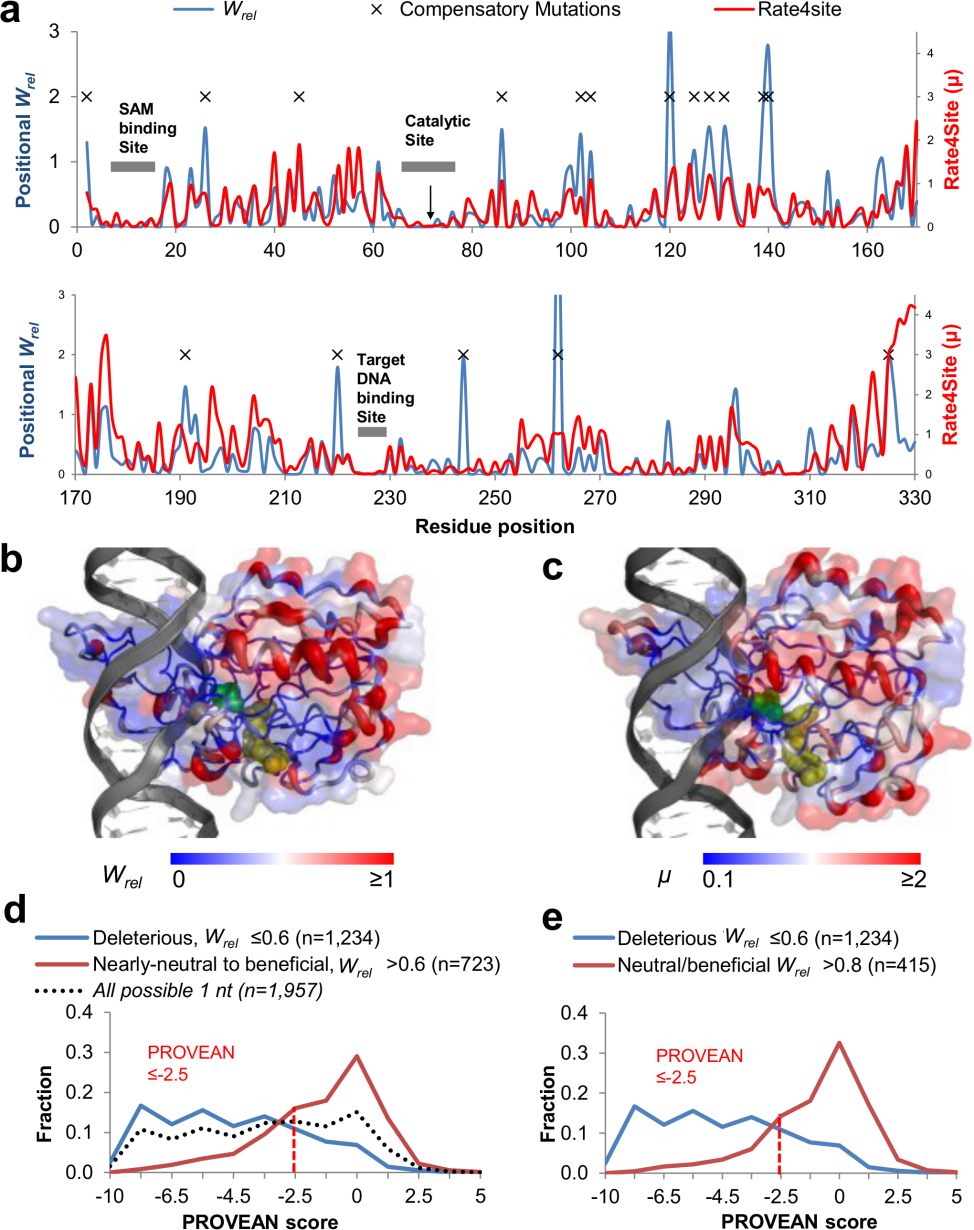
The mutational effects in the laboratory drift correlate with sequence exchanges in M.HaeIII orthologs. **a**. The positional rates of evolution in M.HaeIII’s natural orthologs (‘Rate4site’, μ; red line) were plotted alongside the positional *W*_*rel*_ values in M.HaeIII (blue line). The positional *W*_*rel*_ values correspond to the average *W*_*rel*_ values for all mutations in this position ($\sum_{i}\left\{W_{rel}^{i}\cdot {\text{log}}_{2}\left[1+10\cdot f(G_{17}^{i})\right]\right\}$, Where *i* refers to all the possible single nucleotide mutations at a given residue position. **Upper panel**–positions 2 to 175; **Lower panel**–positions 176 to 330. Noted are M.HaeIII’s key functional residues, those of the cofactor binding site, the catalytic residues including the enzyme’s reaction center (Cys71, black arrow), and the target DNA binding residues. Also noted are positions of compensatory mutations that were enriched in the drift *W*_*rel*_ > 1.1, listed in **S2 Table**). **b.** M.HaeIII’s three-dimensional structure illustrated as a cartoon (PDB id 1dct). Residues are colored from blue to red according to their averaged *W*_*rel*_ values (as in **c**). The cofactor, SAM, is in yellow, and the enzyme’s catalytic residue (Cys71) is in green. **c.** The same as **b** for the positional diversity calculated by Rate4site (μ, as in **c**) [65]. **d.** The distribution of PROVEAN scores for all possible single nucleotide missense mutations (n = 1,957). Shown are the distribution of mutations categorized as ‘deleterious’ (*W*_*rel*_ ≤0.6), and of mutations categorized as ‘nearly-neutral’, ‘neutral’ and ‘beneficial’ (*W*_*rel*_ >0.6). **e.** The same distribution while excluding ‘nearly-neutral’ mutations.

To address this question we first compared the experimental *W*_*rel*_ values to the natural evolutionary rates of the respective positions (**Fig 3A**). We used Rate4Site whereby the calculated rates relate to the degree of physico-chemical change exerted by sequence exchanges, and to the phylogenetic distances [65]. We found that positions that exhibit slow evolutionary rates (*i*.*e*., are highly conserved in nature; log_2_μ ≤ −2, or μ ≤ 0.25 [44]) show no, or low acceptance to mutations in the laboratory drift. Conversely, positions with high evolutionary rates tend to show high experimental tolerance to mutations. This trend is seen along the primary sequence (**Fig 3A**) as well as in the 3-dimensional structure (**Fig 3B and 3C**).

Given that we have mapped the effects of all possible single nucleotide mutations in M.HaeIII, we further examined how well their effects could be predicted from an alignment of orthologous sequences. There are many ways of predicting the effects of mutations from multiple sequence alignments. Certain biases are inevitable; foremost, prediction is highly dependent on sequence sampling–the number and the phylogenetic distribution of available sequences that are evolutionary related to the protein in question. Other biases relate to phylogenetic relatedness of the orthologs to the reference sequence and the manner by which the degree of divergence is calculated. A meaningful measure uses profile scores (position-specific scoring matrices) that take into account not only the frequency of sequences in which a given position varies, but also the physico-chemical nature of exchanges (reviewed in [5, 66]).

Given the epistatic nature of sequence evolution, tolerance of mutations is largely not a matter of 'if' but of 'when'–namely, given enough drift, exchanges in even the most conserved sites may be tolerated [2, 45, 67]. PROVEAN (Protein Variation Effect Analyzer, http://provean.jcvi.org) is a predictor that takes into account phylogenetic distances [68]. Thus, the PROVEAN score function considers the physiochemical impact of amino acid exchanges alongside the evolutionary distance between the reference protein and the homolog(s) in which a given exchange is observed. We submitted to PROVEAN the reference sequence (the wild-type M.HaeIII gene) and a multiple sequence alignment of 105 orthologs (**S5 Fig**). The program computed a score predicting how deleterious each possible amino acid exchange in M.HaeIII might be. The algorithm’s default thresholds are: scores ≤ -2.5 are predicted as deleterious, and scores > -2.5 as neutral [68]. We then compared the predicted PROVEAN score to the measured *W*_*rel*_ values for the 1,957 single nucleotide nonsynonymous mutations.

Overall, a clear-cut trend is seen (**Fig 3D**)–mutations found to be deleterious in the laboratory drift (*W*_*rel*_ ≤ 0.6) tend to show low PROVEAN scores (≤ -2.5), whereas the accepted ones show high scores (> -2.5). From PROVEAN’s point of view as a predictor of deleterious mutations, true positives occurred at a rate of 83.3% (**S3 Table**). Namely, out of the 1,234 mutations that were evidently deleterious in the laboratory drift (*W*_*rel*_ ≤ 0.6), 1,028 were correctly categorized by PROVEAN as deleterious (score ≤ -2.5). True negatives–mutations predicted by PROVEAN as neutral and found to be so in the drift, occurred at a rate of 63.5% (459 out of the 723 accepted mutations in the laboratory drift, *W*_*rel*_ > 0.6, were scored with PROVEAN values of > -2.5).

When excluding mutations with borderline effects (mutations categorized as nearly-neutral, with *W*_*rel*_ values 0.61–0.8), the effects of the remaining set of mutations (1,649 out of 1,957; *W*_*rel*_ ≤0.6, or > 0.8) were, as expected, better predicted by PROVEAN (**Fig 3E**). Specifically, the ability to predict the effect of neutral mutations (*W*_*rel*_ > 0.8) increased to 72%, (accuracy of 80.5%, **S3 Table**). Notably, SIFT, a predictor similar to PROVEAN but that with no phylogenetic correction, showed lower prediction accuracy than PROVEAN (75.3% accuracy, with 73% true positives for deleterious mutations, and 82.2% true negatives for neutral mutations, *W*_*rel*_ > 0.8; **S6 Fig** and **S3 Table**). Furthermore, the effects of mutations are best described on a continuum scale rather than a binary classification of deleterious versus neutral. The inclusion of phylogenetic distances, as in PROVEAN, also generates a continuous score that in turn seems to correlate well with the experimental *W*_*rel*_ values (**S6C** and **S6D Fig**).

Further support to the conclusion that phylogenetic distance is a crucial factor in prediction is provided by the fact that neutral/beneficial drift mutations (*W*_*rel*_ > 0.8) are decreasingly observed in orthologs as their sequences further diverge from M.HaeIII’s. In total, 39% of the single nucleotide exchanges observed in orthologs were found as neutral/beneficial in the background of M.HaeIII (*W*_*rel*_ > 0.8, **S7A Fig**). Of these, 54% (*i*.*e*., 21% of all single nucleotide ortholog-observed exchanges) appear in close orthologs (≤35% divergence relative to M.HaeIII, **S7B Fig**). Thus, sequence exchanges observed in orthologs with higher sequence identity are more indicative of a neutral fitness effect in the reference sequence (M.HaeIII in our case) than when the very same exchanges appear in diverged orthologs (>50% divergence).

### Biophysical constraints

Finally, we examined how the experimentally measured fitness effects correlate with predicted structural and functional constrains, and to what degree these constraints apply to the sequence diversity seen in orthologs of M.HaeIII. To this end, a multiple sequence alignment was derived for M.HaeIII (**S5 Fig**). The alignment gave a set of 2,000 exchanges that are observed in at least one ortholog–dubbed ‘*ortholog-observed*’ (845, single nucleotide exchanges, and the remaining 1,155 being double and triple exchanges). The complementary set of ‘*ortholog-unobserved*’ was accordingly derived, and included 4,251 exchanges that are not observed in any known ortholog (of which 1,112 are single nucleotide exchanges). We then compared the experimental set of ‘neutral mutations’ (*W*_*rel*_ ≥ 0.8, *i*.*e*., excluding ‘nearly-neutral’) to *‘ortholog-observed’*, and the set of ‘deleterious mutation’ *W*_*rel*_ ≤ 0.6) to *‘ortholog-unobserved’*. This comparison is *a priori* problematic. The background at which these two sets of mutations occurred differs fundamentally: the maximal divergence in the laboratory drifted G17 sequences was ~3% (an average of 9.6 mutations per a length of 329 amino acids gene). Accordingly, whereas our experimental set comprises only single nucleotide mutations, most natural amino acid exchanges relate to two or three nucleotide exchanges within the same codon (1,155 out of 2,000 orthologs-observed, and 3,139 out of 4,251 orthologs-unobserved).

**Fig 4:**
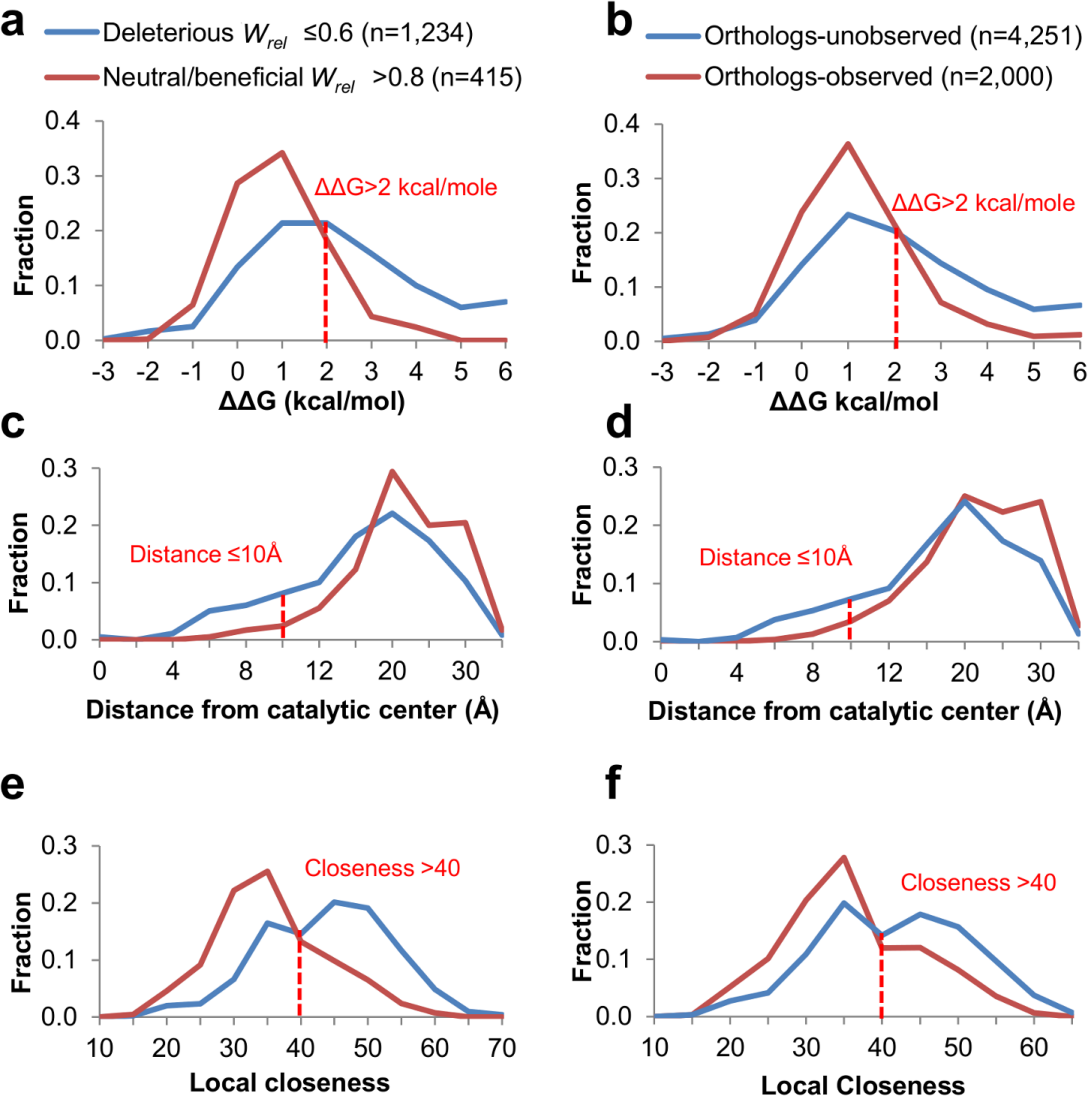
Structural and functional constraints dictate tolerance to mutations in M.HaeIII. The collections of ‘deleterious’ (*W*_*rel*_ ≤ 0.6, n = 1,234) and ‘neutral/beneficial’ (*W*_*rel*_ ≥ 0.8, n = 415) mutations were characterized by different biophysical and functional constraints (left column). The same analysis was performed for all the ‘ortholog-observed’ exchanges (n = 2,000) and ‘ortholog-unobserved exchanges’ (n = 4,251) (right column). **a** and **b**: The distribution of predicted ΔΔG values computed by FoldX [69, 70]. **c** and **d**: The distribution of distances of the residues in which the mutations occurred from M.HaeIII’s reaction center (the closest distance to either the sulfur atom of Cys71 or to the carbon of SAM’s methyl-group). **e** and **f**: The distribution mutations according to the residue predicted ‘local closeness’ [71, 72]. The distributions were analyzed using Kolmogorov–Smirnov test. The red dashed lines indicate the calculated thresholds for defining highly-deleterious according to the critical values with maximum difference between the two distributions, thus indicating *P-Values* <<0.001.

Despite the above caveat, we found that drift mutations with ‘neutral/beneficial’ effect (*W*_*rel*_ > 0.8), and accordingly, *orthologs-observed* exchanges, share the same biophysical constraints with respect to M.HaeIII’s configurational stability and enzymatic function (**Fig 4**). Specifically, mutations predicted using the FoldX force field to be highly destabilizing (ΔΔG ≥ 2 kcal/mol; [69, 70]) were purged in the laboratory drift (‘deleterious’ *W*_*rel*_ ≤0.6) and also in the natural diversity (*‘orthologs-unobserved’*, **Fig 4A and 4B**). Mutations in positions close to M.HaeIII’s active-site followed the same trend (**Fig 4C and 4D**). The overlap between the biophysical constrains acting in nature and in the laboratory constraints was also indicated by ‘local closeness’–a structural measure of the degree of structural connectivity of a residue to other residues [71, 72] (**Fig 4E and 4F**). Furthermore, exchanges found in close orthologs appear to obey the above biophysical constraints to a larger extent than those in more diverged ones [41] (**S7B** and **S7C Fig**).

## Discussion

Our results illustrate the limitations inherent to the experimental methodologies used for measuring the fitness effects of mutations in the laboratory, and in deducing from these experiments how proteins evolve in nature. In general, the current state-of-the-art experimental mappings artificially widen the threshold for acceptance of mutations, such that the early accumulating mutations have no apparent effect on the protein’s fitness [73]. This wider experimental threshold is driven by various factors, including: (*i*) higher protein stability (*e*.*g*., the stabilizing mutations we included in, or fusion tags known to increase solubility (*e*.*g*. [26]); (*ii*) Gene and protein copy numbers that are typically orders-of-magnitude higher than the natural ones; (*iii*) Growth environments that are less demanding than the natural ones; (*iv*) Proteins being under selection for one task out of several tasks they perform in nature under variable conditions. Such wider thresholds result in a higher, if not unrealistic tolerance of mutations relative to nature [74, 75].

Once this threshold is exhausted, the loading of additional mutations results in a rapid collapse [47]. Indeed, the dynamics of our neutral drift experiment indicate that the very same deleterious mutations, including nonsense mutations, which are tolerated in the early rounds, are completely purged as the drift progressed (**Figs 1C and S4**; see also [76]).

The continuous loading of mutations, as applied in our study, appears to portray a more realistic picture with respect to the fraction of deleterious mutations. The distribution of fitness effects of mutations (DFE) derived from this experiment is different from the distributions derived from previous experiments, namely that ~30% of mutations are deleterious, and the remaining largely neutral [25–27, 33, 46]. In contrast, this experiment indicates the anticipated continuum, rather than the generally assumed bimodal distribution [49, 74, 77] (**Fig 1A**). Further, even under the most conservative threshold, 67% of the mutations have evident deleterious effects (*W*_*rel*_ ≤ 0.6). However, purging is also consistently seen for mutations we categorized as ‘nearly-neutral’ (*W*_*rel*_ = 0.61–0.8, **Fig 1B**). Individually these mutations may be close to neutrality, but collectively they impact fitness, analogously to a population’s drift load [50, 78]. This is apparent by the acceptance of ‘nearly-neutral’ mutations being accompanied by the enrichment of compensatory mutations (**Fig 1**) [79]. If what we categorized as nearly-neutral mutations are included, ~81% of all possible amino acid mutations that derived from single nucleotide mutations are potentially deleterious.

The much higher fraction of nonsynonymous mutations with deleterious effects observed in our experiment as compared to other experiments may relate to variations in the mutational tolerance of one protein *vs*. another. However, M.HaeIII does not seem to be a particularly slow evolving protein–the distribution of the positional evolutionary rates, and specifically the relative histogram areas of the fast versus slow evolving positions, are in agreement with a fast evolving protein [44]. Regardless, it is clear that, at present, comparing the DFEs obtained for different proteins is problematic because the experimental methodologies used to obtain these DFEs vary so much.

The sensitivity of detection of fitness effects is also limited in laboratory setups by high noise levels as well as by the limited number of generations along which fitness is examined [74]. We also note that in reality, protein sequences drift in a gradual manner and via single nucleotide exchanges. Thus, the fitness effects measured for all 19 possible amino acids per position often reflect leaps in sequence space that are not taken by natural evolution.

The results of our laboratory drift also support the hypothesis that natural protein drift is punctuated by deleterious and compensatory mutations. The order of their accumulation may differ, also in relation to the mutational rates. At high mutational rates, as applied here, compensatory substitutions may follow the deleterious ones [80–83]. At low mutation rates, however, mutations that initially accumulated as neutral may enable the fixation of deleterious ones [44, 83]. In any case, the DFE obtained here suggests that, whereas upon drifting in nature, exchanges may be fixed by chance (the neutral theory), their fitness effects are rarely neutral–they are nearly always deleterious or compensatory [41, 84] (**Fig 1A**).

Compared to previous reports (for example see [25–27, 33, 36]), tolerance *vs*. purging of mutations in our prolonged drift shows much better correlation to the positional evolutionary rates, and to specific exchanges observed in the natural diversity, *i*.*e*., in M.HaeIII’s orthologs (**Fig 4**). Such a correlation is *a priori* problematic. The representation of natural sequences is sporadic, especially with horizontally transferred genes that encode specialized functions such as M.HaeIII. Thus, that a certain exchange is not observed, or rarely observed in the currently known sequence does not necessarily mean it is deleterious. Nonetheless, our data seems to coincide with what had been deduced from other analyses of orthologous sequences, namely that at a given background, the vast majority of mutations are deleterious [39–41, 43] (**Fig 1**). Our data also support the notion that the exchanges found in close orthologs are more likely to be neutral than those in more diverged ones [41] (**S7 Fig**). Exchanges in highly diverged orthologs are tolerated by virtue of being compensated by exchanges at other positions [41, 45, 67] and therefore tend to be context-specific. However, despite the above caveats, it seems that the effects of mutations can be predicted from the natural diversity of orthologs with relatively high accuracy, particularly when ‘nearly-neutral’ mutations with borderline effects are excluded (**Fig 3E**). A systematic exploration of the performances of various predictors and prediction parameters is beyond the scope of this work. Nonetheless, it appears that the prediction seems improved when the phylogenetic distance of orthologs is taken into account [85, 86] (**S6**and **S7 Figs**). Likewise, comparing the results of different experimental mappings of mutational effects is inherently problematic. These mapping experiments used different proteins, different mutagenesis and screening, or selection, strategies, and different ways of assigning the ‘fitness’ values to mutations. As experimental approaches of systematic mapping develop further, standard experimental and data analysis procedures may develop that will enable more meaningful comparisons.

Further, the biophysical constraints acting to limit drift both in the laboratory and in nature overlap, indicating universal constraints that dictate purging of sequence exchanges [87] (**Fig 4**). Thus, including structural considerations, possibly ‘local closeness’ as an integrated parameter [71], may greatly improve prediction of the effect of mutations [88, 89], as already shown for certain predictors [8, 89–93].

Overall, the application of the experimental setup described here provides a better understanding of how protein sequences diverge in nature, as well as a new dataset that can be used for improving the prediction accuracy of the effects of protein mutations, and specifically of single nucleotide polymorphisms.

## Methods

### Plasmids and strains

A modified M.HaeIII wild-type gene, carrying four stabilizing mutations [51], and no GGCC sites in its open reading frame, was cloned with an N-terminal His-tag into pASK-IBA3+ vector (IBA, using NcoI and NotI; the vector also carried 14 GGCC sites, 3 of which were located within the ampicillin resistance gene (See supplementary Fig 3 in [51]). Plasmids were transformed into *E*. *coli* ER2267 (*Eco*K r- m- McrA- McrBC-Mrr-) in which GGCC DNA methylation is not toxic [94]. Transformants were selected by growth on ampicillin.

### Mutagenesis and selection

Random mutagenesis was performed as described previously [52]. Briefly, M.HaeIII's ORF (open reading frame) was amplified by PCR with an error-prone polymerase (GeneMorphII Mutazyme, Stratagene). The mutagenic PCR was optimized to an average of 2.2 mutations per gene. Each round of evolution, or generation (noted as 'G'), included the following steps (**S1 Fig**): (*i*) The pool of M.HaeIII genes from the previous round was randomly mutated, recloned using the NcoI and NotI sites, transformed into *E*. *coli* and plated on agar plates containing ampicillin. About 10^6^ individual transformants were obtained in each round. (*ii*) Colonies grown at 37°C overnight were combined, plasmid DNA was extracted and digested with HaeIII (10–20 units, in 50 μl of NEB buffer 2, for 2 hours at 37°C), and re-purified (PCR purification kit, QIAGEN). (*iii*) The recovered plasmid DNA was re-transformed for another round of enrichment. Each round of drift included one cycle of mutagenesis and three cycles of enrichment (transformation, growth, plasmid extraction and digestion). The naive library, G0, relates to the transformed plasmid DNA derived from cloning of a repertoire of ~10^5^ individual M.HaeIII genes after the first round of mutagenesis and prior to selection by HaeIII digestion.

### High-throughput sequencing

The samples of the naive (G0) and the selected libraries from Rounds 3, 7 and 17 (assigned as G3, G7 and G17) were prepared as described previously [52]. Briefly, the pools of M.HaeIII's open reading frame were PCR-amplified, purified, and concatenated by self-ligation (using XhoI restriction sites at both ends of the PCR [51]). Sequencing libraries were prepared and sequenced according to manufacturer's protocol at the Weizmann Institute's high throughput-sequencing core facility. The obtained sequencing reads (~40 nts) were mapped to the reference sequence of wild-type M.HaeIII with two methods: (*i*) Using NCBI blastn v2.2.20 [95] with parameters: e-value cutoff 0.0001, word size 7, and allowing up to 6 mismatches and requiring a minimal alignment length of 24 consecutive nts, as previously described [96, 97]; and (*ii*) Using Novoalign v2.07.00 with parameters: c 4 Hash step-size 6 [96]. Point mutations, insertions and deletions were assigned based on the mapping of the sequencing reads to the reference sequence as previously described [97, 98]. Every mismatch or gap in the reads alignment relative to the wild-type reference was recorded per each nucleotide position, and further analyzed using custom Perl scripts (available at: https://github.com/tawfiklab/HTS_codon_analyzer). Only codons that were intact within the 40 nt reads were included.

### Primary data analysis and relative fitness values

Processing of the observed mutation counts per codon was done primarily with Excel (see **S1 File**). All possible single nucleotide mutations were detected in the raw data of the unselected G0 library (329X9 = 2,961 possible single nucleotide mutated codons that in turn comprise the 1,957 possible single nucleotide amino acid mutations; **S2 File** and **S8 Fig**). However, Illumina sequencing exhibits a considerable background level of mutagenesis due to PCR amplifications as well as sequencing errors. Potential sequencing artifacts, specifically mutations that were observed at the edges of reads (where sequencing errors are more frequent), were filtered out (**S1 File**). The background rate was determined using the region upstream of the randomly mutated open reading frame of M.HaeIII (the N-terminal fused His tag that was not subjected to mutagenesis, **S2 File**, **S8 Fig** and **S4 Table**). Thus, the average background frequency was subtracted from the mutational frequencies to give the net positional frequencies that were used to calculate the *W*_*rel*_ value of each amino acid mutation (the final analyzed data can be found in **S3 File**, including for double and triple mutations that were not analyzed here).

Mutational frequencies were determined for every possible codon mutation (63 including single, double and triple nucleotide mutations) as the number of reads with a given mutation(s) divided by the total number of reads that mapped the corresponding position. The frequencies of all mutational events that led to the same amino acid were combined.

#### *W*_*rel*_ calculations

Eq (1) (see Results section) can be written as:

$$\begin{array}{l} f(G_{n})=\left[f(G_{n-1})+f(G_{0})\right]\cdot W_{rel}=\\ f(G_{0})\cdot \left\{W_{rel}^{n}+W_{rel}^{n-1}+W_{rel}^{n-2}\ldots +W_{rel}\right\}=f(G_{0})\cdot \sum_{n=1}^{n}W_{rel}^{n} \end{array}$$

Thus, per given mutation, at a given round, *G*_*n*_, the ratio of frequency of this mutation relative to its frequency of occurrence (*f*(*G*_0_)) is given by:

$$\frac{f(G_{n})}{f(G_{0})}=\sum_{n=1}^{n}W_{rel}^{n}$$ (2)

The sum of a geometrical series with n>5 has no closed solution (*i*.*e*., a finite number of *W*_*rel*_ values, let alone one value). We therefore derived numerical solutions for Eq (2), using a series of *W*_*rel*_ values from absolutely deleterious (*W*_*rel*_ = 0) to highly enriched (*W*_*rel*_ = 3.5), thus deriving the expected ratios of mutational frequencies per each round ($\frac{f(G_{n})}{f(G_{0})}$) as a function of *W*_*rel*_ (**S3 Fig**).

### Phylogenetic analysis and evolutionary rates

Orthologous sequences to M.HaeIII were collected using BLASTP search within the REBASE database [99]. Within the range of 25–75% identity, 105 non-redundant family members were aligned using MUSCLE [100] (**S5 Fig**). The maximum likelihood phylogenetic tree was calculated using PhyML with the LG matrix [101]. The position-specific evolutionary rates (μ) were calculated by Rate4Site [65]. The positional rate is calculated that indicates how fast this site evolves relative to the average rate across all sites in the input alignment.

Mutation-specific scores were calculated using PROVEAN and SIFT programs using the default parameters and M.HaeIII sequence as a reference. Both software are available on the homepage of the J. Craig Venter Institute: the SIFT tool is at http://sift.jcvi.org [102], and the PROVEAN tool is at http://provean.jcvi.org [68]. To ensure the consistency of this analysis, we provided the set of orthologs sequences in FASTA format (**S5 Fig**) rather than using the BLAST search in the PROVEAN webserver. The PROVEAN calculated scores were subsequently provided by Dr. Yongwook Choi.

### Stability calculations and structure based measures

FoldX was used to predict the stability effects of mutations relative to wild-type M.HaeIII. The crystal structure of M.HaeIII (PDB id 1dct) [103] was first optimized using the FoldX RepairPDB function. Subsequently, all possible single mutants (19 different amino acids, at each position) were calculated by the BuildModel mutation engine, and relative stability of mutants was obtained (ΔΔG = ΔG_WT_-ΔG_MUT_). Distances of residues from the reaction center were defined as the shortest distance between the closest residue atom and either the sulfur of the catalytic cysteine or the methyl group of the SAM cofactor. These were calculated based on M.HaeIII in complex with the DNA (PDB id 1dct) [103]. The coenzyme distances were derived from a homology model based on M.*Hha*I in complex with SAM (PDB id 2hr1). Local closeness was calculated SPACER web server (available at http://allostery.bii.a-star.edu.sg/) [71] using default parameters and M.HaeIII structure as a reference (PDB id 1dct) [103].

## Supporting Information

S1 File Data processing to obtain the mutational frequencies. (DOCX)

S2 File The raw data frequencies from deep sequencing of the naïve (G0) and selected libraries (G3, G7 and G17). (XLSX)

S3 File Processed data and the net frequencies of mutations in G0 to G17. (XLSX)

S1 Fig A schematic description of the laboratory genetic drift (taken from [52]). M.HaeIII's open reading frame was randomly mutated by error-prone PCR. The mutated genes were cloned into the pASK vector, and the resulting plasmid library was transformed to *E*. *coli*. Following the first round of mutagenesis and cloning, high-throughput sequencing was performed to map the occurrence of mutations irrespective of selection (G0, or the naive repertoire). Subsequently, the plasmid library was subjected to a purifying selection. Within each transformed cell, the expressed methyltransferase variant, if active, methylated its encoding plasmid at GGCC sites and thereby protected it from digestion by the cognate, HaeIII restriction enzyme. Following digestion with HaeIII, the surviving plasmids were retransformed, and subjected again to restriction for further enrichment of plasmids encoding functional methylase variants. After two cycles of enrichment (digestion and transformation), the plasmid DNA was extracted, and the surviving M.HaeIII genes were amplified and randomly mutagenized (as a pool) for the next round. The plasmid library derived from the 3rd, 7th and 17th round of mutagenesis and purifying selection was also subjected to high-throughput sequencing, thus mapping the repertoire of tolerated mutations (G3, G7 and G17).
(PDF)

S2 Fig The mutational patterns in the naïve, G0, and selected, G17, gene libraries. **a.** The pattern of mutation types in the unselected library (G0). The distribution of mutational frequencies in the G0 library was plotted for each type of transition or transversion mutation. The "central box" represents the ranges for 50% of the frequencies, and its lower and upper boundary lines are at the 25^th^ and 75^th^ percentiles of the data. The horizontal central line indicates the median of the data. The two vertical lines extending from the central box indicate the remaining frequencies outside the central, 50% box, except those frequencies regarded as outliers (shown as circles). **b.** The observed mutation frequencies of synonymous mutations in G3 (a selected library) is strongly correlated with the observed frequencies in G0, the unselected library. **c.** In G17, the correlation with G3 frequencies of nonsynonymous mutations is much weaker, probably due to selection **d**. The expected rate of synonymous mutations as calculated from the G0 substitution matrix (shown in panel **a**) shows a weak correlation with the observed mutation frequencies in G3. **e.** The observed mutation frequencies of synonymous mutations in G0 (left axis) and G3 (right axis) along the M.HaeIII amino acid residues.
(PDF)

S3 FigDetermination of the relative fitness values (*W*_*rel*_) of mutations.
**a**. The calculated ratios of mutational frequencies in the selected libraries (G3, G7, G17) relative to their frequency of occurrence in G0 ($\frac{f(G_{n})}{f(G_{0})}$) as a function of their relative fitness effect (*W*_*rel*_) using Eq (1) (see main text). **b-d.** The *W*_*rel*_ values of mutations measured for G3, G7 or G17 are correlated (Slopes: 0.93, 0.86 and 0.82; R^2^ = 0.46, 0.5 and 0.57 for the correlations measured in **b-d**, respectively).(PDF)

S4 Fig The relative fitness effects of nonsense mutations. **a**. The *W*_*rel*_ values for nonsense, stop codon, mutations observed in M.HaeIII along the 3 rounds of the drift (G3 –Green; G7 –Red; G17 –Blue). The ‘Red arrows’ show positions 176-permissive position only at the onset of the drift; and 324—after which, stop codon mutations seem not to be purged as indicated by *W*_*rel*_ values close to 1. **b.** The total frequency of nonsense mutations along the drift. The purging is stronger when positions after 324 are not included (red bars) relative to the entire gene including positions 325–329 (blue bars).
(PDF)

S5 Fig Multiple sequence alignment of M.HaeIII and its 105 orthologs. Orthologous sequences to M.HaeIII were collected using BLASTP search within the REBASE database [99]. Within the range of 25–75% identity, 105 non-redundant family members were identified and subsequently aligned using MUSCLE [100].
(PDF)

S6 Fig The observed fitness effects of mutations in the laboratory drift compared to the predicted effect by SIFT and PROVEAN. **a.** The distribution of SIFT scores for the single nucleotide mutations observed in the selected ensembles of the laboratory drift, G17 (n = 1,957). The drift mutations were categorized according to their relative fitness effects (*W*_*rel*_; as in **Fig 1**). **b.** The same distribution after ‘nearly-neutral’ mutations were excluded: for the ‘deleterious’ mutations (*W*_*rel*_ ≤0.6) and ‘neutral/beneficial’ (*W*_*rel*_ >0.8). **c.** The correlation of *W*_*rel*_ values with the SIFT scores. **d.** The correlation of *W*_*rel*_ values with PROVEAN scores.
(PDF)

S7 Fig Acceptance in the laboratory drift correlates with the phylogenetic distance. **a.** The single nucleotide mutational space (n = 1,957) was categorized according to whether the same mutation is seen in M.HaeIII orthologs (observed; n = 845), or not (unobserved; n = 1,112). Within each category, the exchanges were assigned as ‘beneficial’ (*W*_*rel*_ > 1.1, oxblood), ‘Neutral’ (*W*_*rel*_ > 0.8, ≤1.1, red), ‘Nearly-neutral’ (*W*_*rel*_ > 0.6, ≤0.8, grey) or ‘Deleterious’ (*W*_*rel*_ ≤0.6, blue) according to their relative fitness effects in the laboratory drift (*W*_*rel*_ values in G17, as in **Fig 1**). **b.** The single nucleotide mutations were further divided according to their appearance in orthologs with different levels of sequence divergence relative to M.HaeIII (fraction of amino acids divergence of the closest ortholog in which a given mutation/exchange was found). **c.** The distributions of biophysical and functional constraints (as in **Fig 4**) and PROVEAN score (as in **Fig 3**) for the fractions of all the single nucleotide mutations according to their relative fitness effects in the laboratory drift (*W*_*rel*_ values in G17, as in **Fig 1**). **d.** The distributions of biophysical and functional constraints (as in **Fig 4**) and PROVEAN score (as in **Fig 3**) for the fractions of all the ‘orthologs-observed’ exchanges (2,000 exchanges in total) with varying degrees of divergence, and for ‘ortholog-unobserved’ exchanges (4,251 exchanges).
(PDF)

S8 Fig Distribution of the observed mutational frequencies for M.HaeIII’s ORF and the non-mutated region (background frequencies). **‘**Data’ relates to the distributions of the measured, raw mutation frequencies (*i*.*e*. prior to background subtraction) in each library within the coding region of M.HaeIII's (329 resides, in blue color, derived from **S2 File**). ‘Background’ relates to the distributions of the raw mutational frequencies in the region located upstream of the cloning sites, a region that was not subjected to mutagenesis (20 residues including His-tag and Thrombin cleavage site, residues -20 to -1, in red; **S2 File**). The average background frequency was subtracted from all measured frequencies, thus eliminating the effect of mutations that accumulated in the Illumina sequencing (**S3 File** and **S4 Table**).
(PDF)

S1 Table Average mutational frequencies per types of base exchanges. (PDF)

S2 Table Compensatory mutations observed in the laboratory drift. Compensatory mutations were defined as enriched mutations, either by assigned beneficial fitness effect for individual mutations by (*W*_*rel*_ >1.1) or high positional fitness effect (the averaged *W*_*rel*_ per position as calculated in **Fig 3A,** *W*_rel (Positional)_ >1.1). Shown are the *W*_*rel*_ of mutations that were enriched in the selected G17 library. Also noted are the sequence divergence and the frequency of these exchanges in the natural diversity relative to M.HaeIII, and the frequency of the mutation under the selection for new functions [51].
(PDF)

S3 Table Prediction accuracy of PROVEAN and SIFT. ‘Sensitivity (TPR)’–True positives rate; correctly identified as deleterious mutations, TP, out of the total deleterious mutations. ‘Specificity (TNR)’- True negatives rate; correctly identified as neutral mutations, TN, out of the total neutral mutations. ‘FPR’–false positive rate; incorrectly identified as deleterious mutations, FP, and are in fact neutral out of the total neural mutations. ‘FNR’—false negative rate; incorrectly identified as neutral mutations, FN, and are in fact deleterious out of the total neural mutations. Accuracy = (TP + TN)/(TP + TN + FP + FN)
(PDF)

S4 Table The raw data and above frequencies mutated codons in the different libraries. 'Threshold'—refers to the background frequencies observed in each library at the unmutated region. "≤ Threshold"—refers to the number of codons in the mutated M.HaeIII's ORF in each library with frequencies below the threshold that were excluded from the analysis. "> Threshold"—refers to the number of codons in the mutated M.HaeIII's ORF in each library with frequencies above the threshold, and thus were included in the analysis.
(PDF)
