# Supplementary material for: Systematic Mapping of Protein Mutational Space by Prolonged Drift Reveals the Deleterious Effects of Seemingly Neutral Mutations
Source: PLoS Comput Biol. 2015 Aug 14;11(8):e1004421. doi: 10.1371/journal.pcbi.1004421 (PMC4537296; doi:10.1371/journal.pcbi.1004421)
Supplement: S1 File — (DOCX) [file pcbi.1004421.s001.docx]

**Systematic mapping of protein mutational space by prolonged drift reveals the deleterious effects of seemingly neutral mutations**

Liat Rockah-Shmuel, Ágnes Tóth-Petróczy & Dan S. Tawfik

**File S1: Data processing to obtain the mutational frequencies**

- All reads from the naïve (G0) and the selected libraries (G3, G7 and G17), were aligned. The various codons at each position were counted using a Perl, provided that all 3 bases had a quality score ≥ 20; otherwise the read was ignored (available at: [https://github.com/tawfiklab/HTS_codon_analyzer](https://xmail.weizmann.ac.il/owa/redir.aspx?C=orD9-qE-4U-kBgL_DvrZkdz07-osYNIIncq4IA2UDoV6oHjXsjqxtY9M2dNbd5OtCTB22MWZeKw.&URL=https%3a%2f%2fgithub.com%2ftawfiklab%2fHTS_codon_analyzer)).
- The counts for each codon, at a given residue of the reference sequence, were distributed according to their position along the reads identified in the aligned data (*i.e.*, counts were binned from 1 to 40 for each codon at a given residue).
- For each codon at a given residue of the reference sequence, a weighted average score reflecting its distribution of positions along the reads was computed in the following way:

$${\sum_{i=1}^{40} ({counts}_{i}\cdot i)}/{\sum_{i=1}^{40} {counts}_{i}}$$

Where *i* is the position of the observed counts (1 to 40)

- Only mutations who were identified with no severe biases with respect to their location in the 40 bp reads were included (*i.e.*, the score was higher than 2 and smaller than 38).
- The frequencies were derived by summing of all the counts for a given codon, and dividing by the total counts for the corresponding residue (raw data can be found at **File S2**).
- The sequence stretch included the coding region of M.HaeIII's that was repetitively mutated and re-cloned into the selection plasmid (329 resides, residues 2 to 330, blue color in **File S2**), as well as a region located 3’ upstream of the cloning site that was not subjected to mutagenesis (20 residues including His-tag and a thrombin cleavage site, residues -20 to -1, red color in **File S2**). The latter was used to determine the background frequency, namely the frequency of mutations due to the Illumina processing. The background frequencies were derived for all sequenced repertoires, G0, G3, G7 and G17, and the average background frequency was accordingly derived per each repertoire (see **Fig. S8**). The average background frequencies were subtracted from the mutational frequencies measured at the 329 residues subjected to drift (**Table S4**).
- The presence of double and triple mutations at the background region was extremely low. As the scope of this paper was focused analysis of single nucleotide mutations, we used arbitrary threshold for the background frequencies of double and triple mutations, that were set as 1/4^th^ and 1/16^th^, respectively, of the background frequencies for single nucleotide mutations ($\bar{X}$/4 and $\bar{X}$/16 in **Fig. S8**, see also **Table S4**). However, future analysis can be done on the raw data represented in **File S2**.
- Overall, within the naïve (G0) and the three selected libraries (G3, G7, G17), 2,961 single nucleotide mutations were initially identified along all 329 codons of M.HaeIII’s ORF. Following the filtering (for the distribution with the reads and background reduction), 2,907 identified with a frequency above background (described in **Table S4**) and these comprised the data set described here (**File S3**).
