# Supplementary figures and images for: Systematic Mapping of Protein Mutational Space by Prolonged Drift Reveals the Deleterious Effects of Seemingly Neutral Mutations
Source: PLoS Comput Biol. 2015 Aug 14;11(8):e1004421. doi: 10.1371/journal.pcbi.1004421 (PMC4537296; doi:10.1371/journal.pcbi.1004421)

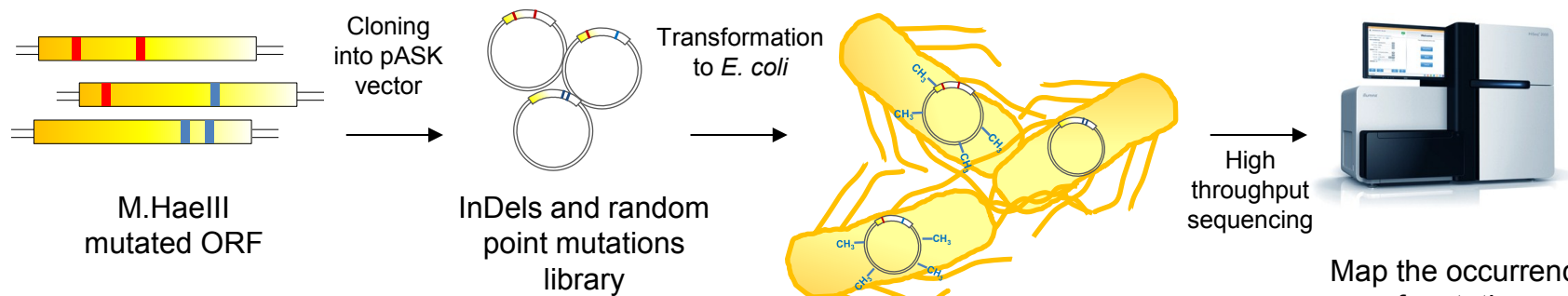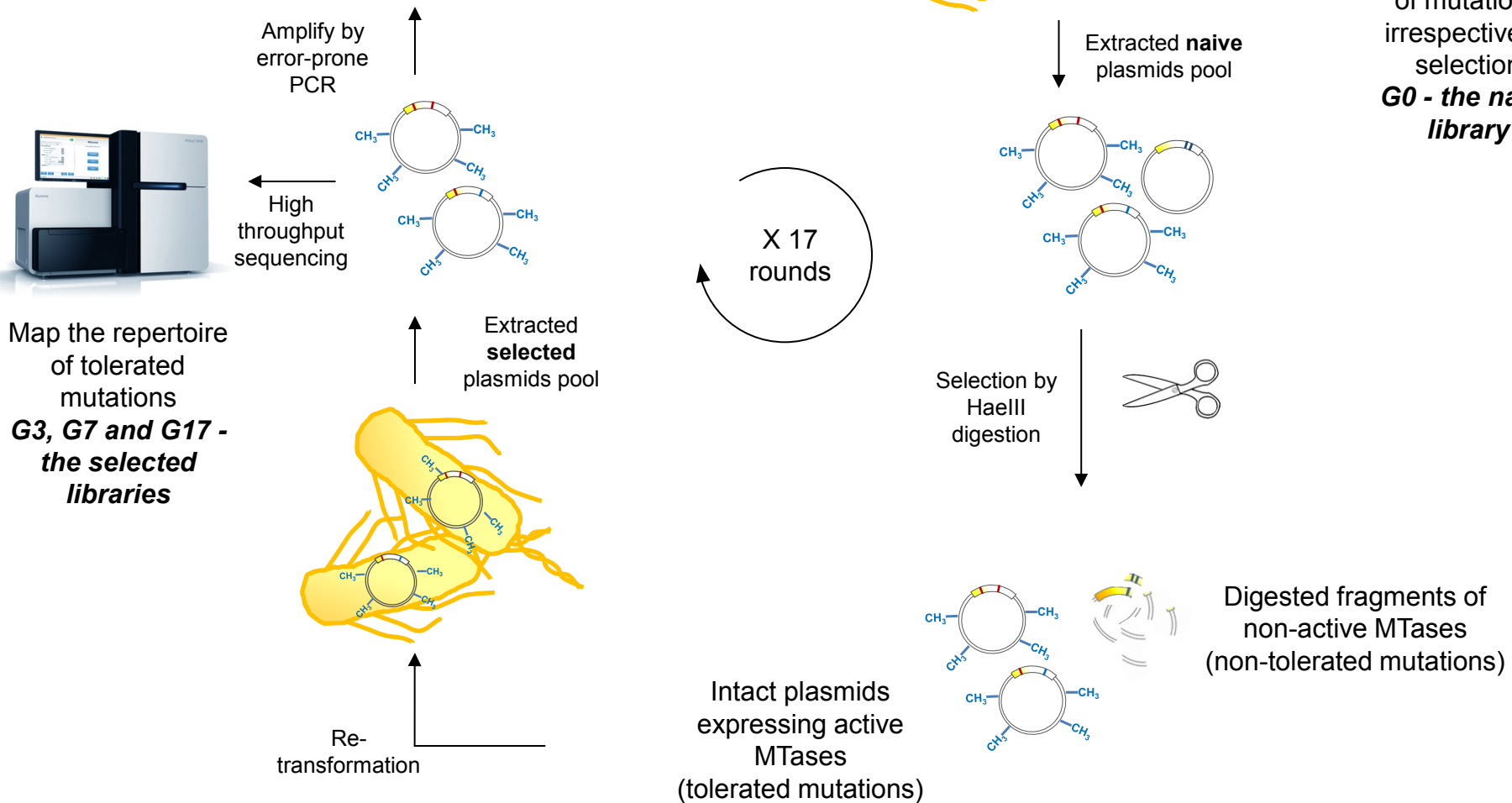

Supplement: S1 Fig — M.HaeIII's open reading frame was randomly mutated by error-prone PCR. The mutated genes were cloned into the pASK vector, and the resulting plasmid library was transformed to E. coli. Following the first round of mutagenesis and cloning, high-throughput sequencing was performed to map the occurrence of mutations irrespective of selection (G0, or the naive repertoire). Subsequently, the plasmid library was subjected to a purifying selection. Within each transformed cell, the expressed methyltransferase variant, if active, methylated its encoding plasmid at GGCC sites and thereby protected it from digestion by the cognate, HaeIII restriction enzyme. Following digestion with HaeIII, the surviving plasmids were retransformed, and subjected again to restriction for further enrichment of plasmids encoding functional methylase variants. After two cycles of enrichment (digestion and transformation), the plasmid DNA was extracted, and the surviving M.HaeIII genes were amplified and randomly mutagenized (as a pool) for the next round. The plasmid library derived from the 3rd, 7th and 17th round of mutagenesis and purifying selection was also subjected to high-throughput sequencing, thus mapping the repertoire of tolerated mutations (G3, G7 and G17). (PDF) [file pcbi.1004421.s004.pdf]

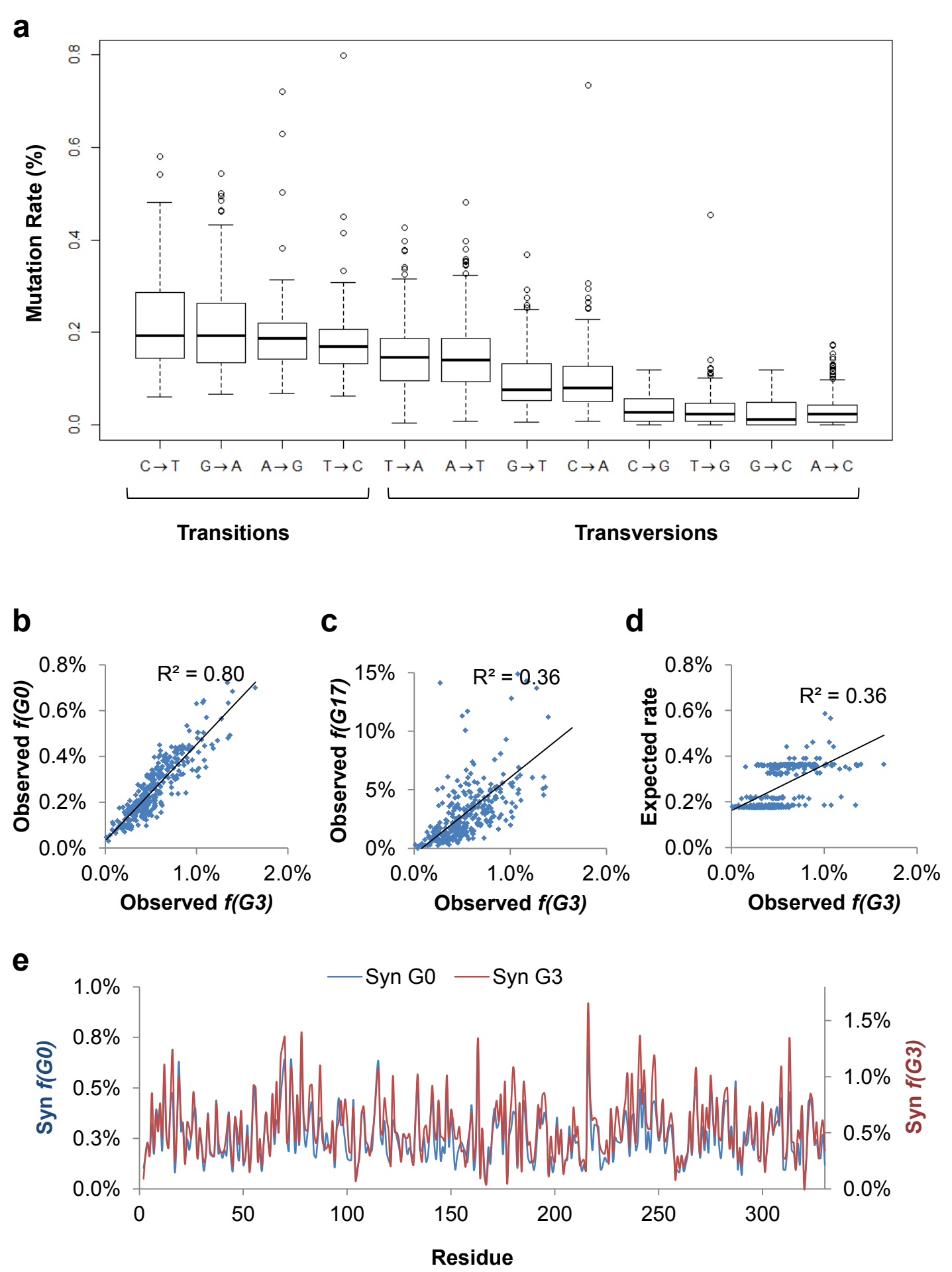

Supplement: S2 Fig — a. The pattern of mutation types in the unselected library (G0). The distribution of mutational frequencies in the G0 library was plotted for each type of transition or transversion mutation. The "central box" represents the ranges for 50% of the frequencies, and its lower and upper boundary lines are at the 25th and 75th percentiles of the data. The horizontal central line indicates the median of the data. The two vertical lines extending from the central box indicate the remaining frequencies outside the central, 50% box, except those frequencies regarded as outliers (shown as circles). b. The observed mutation frequencies of synonymous mutations in G3 (a selected library) is strongly correlated with the observed frequencies in G0, the unselected library. c. In G17, the correlation with G3 frequencies of nonsynonymous mutations is much weaker, probably due to selection d. The expected rate of synonymous mutations as calculated from the G0 substitution matrix (shown in panel a) shows a weak correlation with the observed mutation frequencies in G3. e. The observed mutation frequencies of synonymous mutations in G0 (left axis) and G3 (right axis) along the M.HaeIII amino acid residues. (PDF) [file pcbi.1004421.s005.pdf]

**a**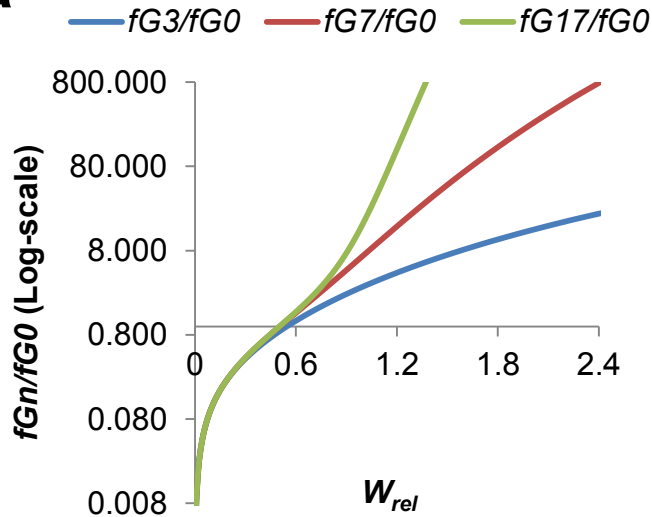**b**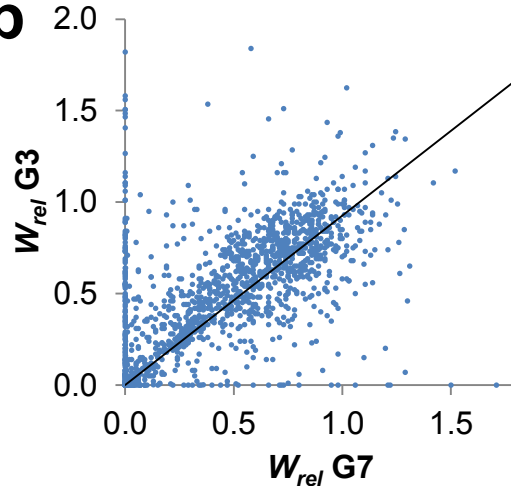**c**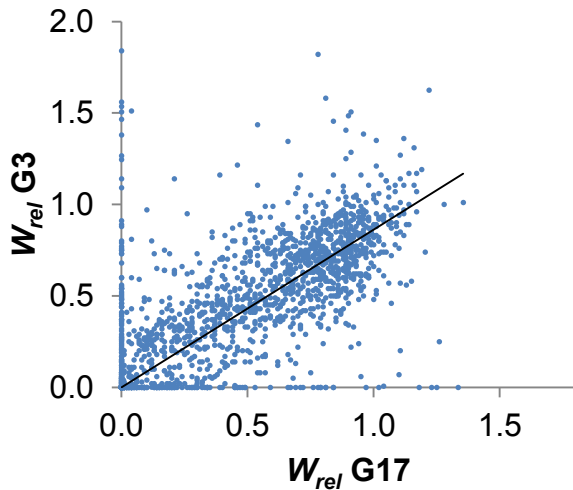**d**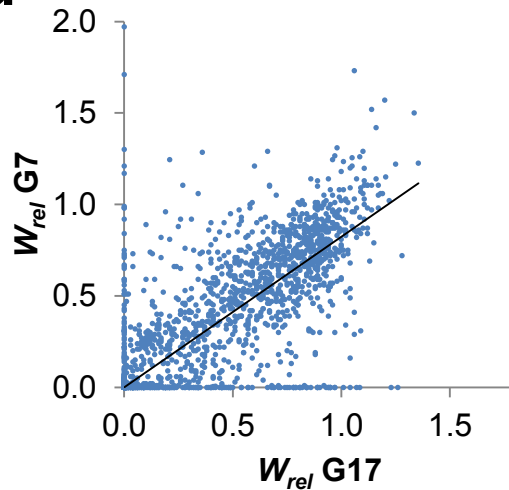

Supplement: S3 Fig — a. The calculated ratios of mutational frequencies in the selected libraries (G3, G7, G17) relative to their frequency of occurrence in G0 (f(Gn)f(G0)) as a function of their relative fitness effect (W rel) using Eq (1) (see main text). b-d. The W rel values of mutations measured for G3, G7 or G17 are correlated (Slopes: 0.93, 0.86 and 0.82; R2 = 0.46, 0.5 and 0.57 for the correlations measured in b-d, respectively). (PDF) [file pcbi.1004421.s006.pdf]

**a**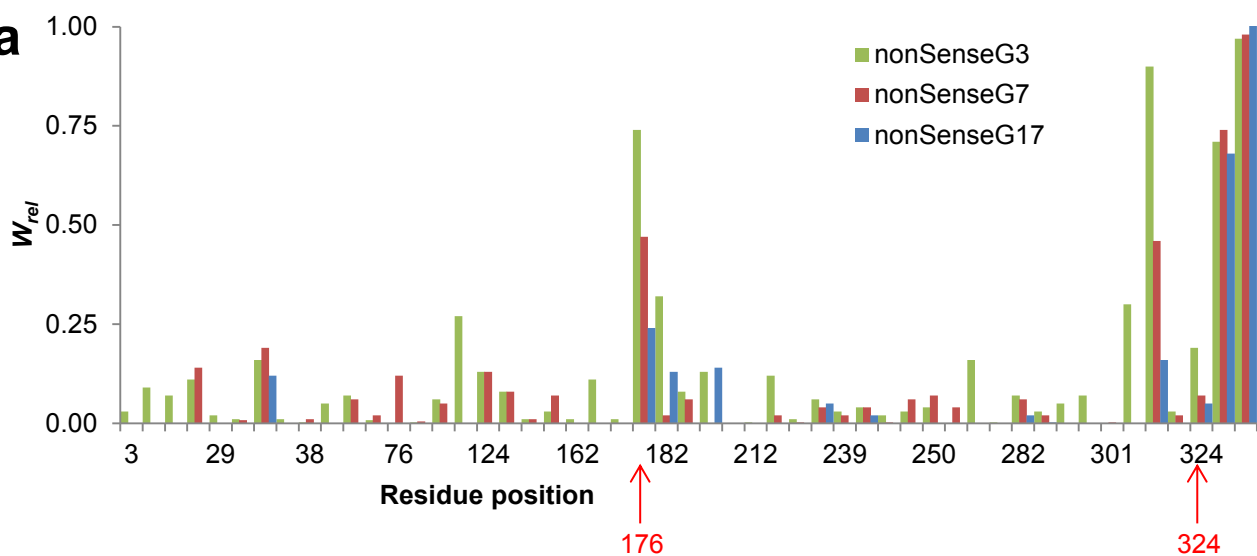**b**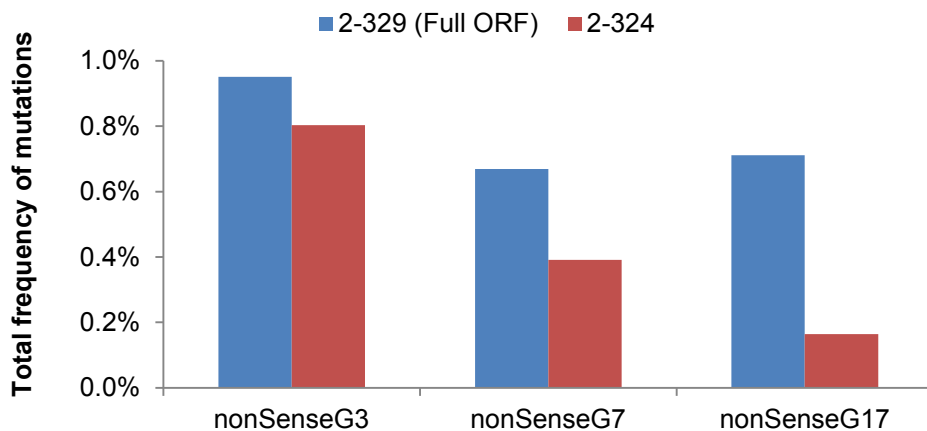

Supplement: S4 Fig — a. The W rel values for nonsense, stop codon, mutations observed in M.HaeIII along the 3 rounds of the drift (G3 –Green; G7 –Red; G17 –Blue). The ‘Red arrows’ show positions 176-permissive position only at the onset of the drift; and 324—after which, stop codon mutations seem not to be purged as indicated by W rel values close to 1. b. The total frequency of nonsense mutations along the drift. The purging is stronger when positions after 324 are not included (red bars) relative to the entire gene including positions 325–329 (blue bars). (PDF) [file pcbi.1004421.s007.pdf]

**a**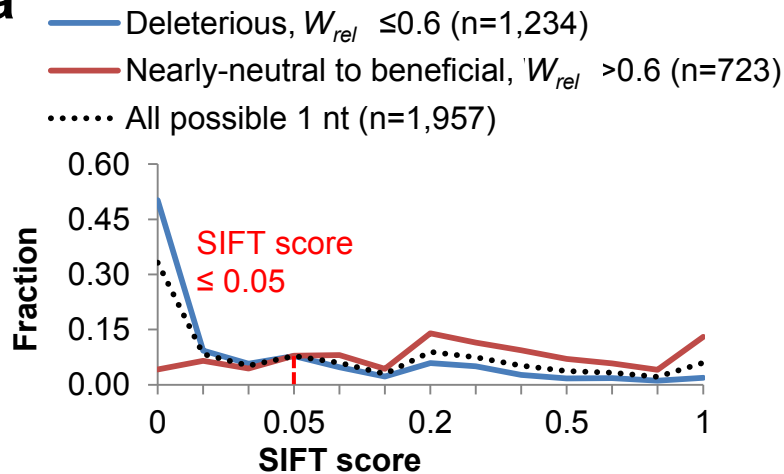**b**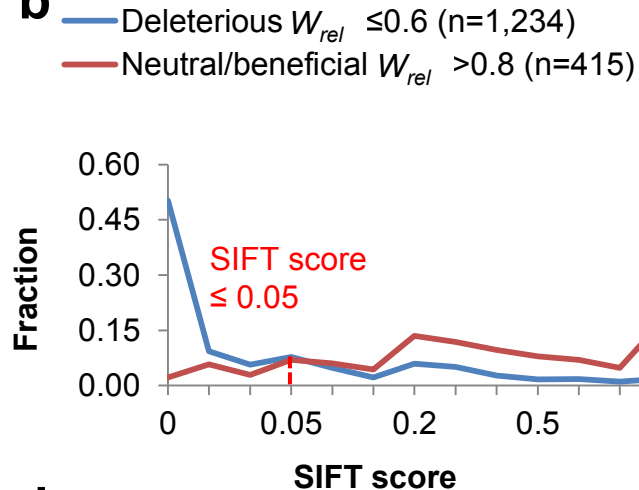**c**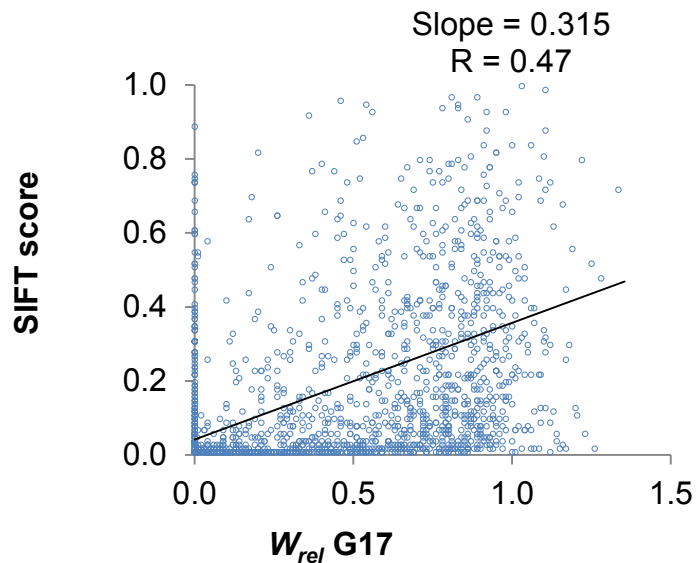**d**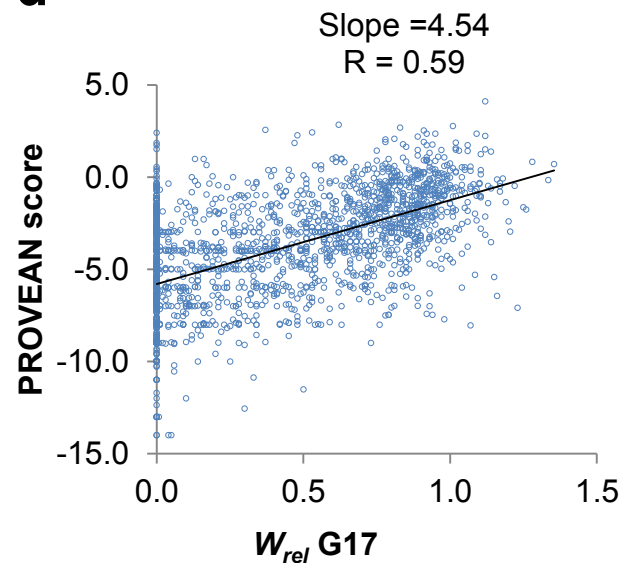

Supplement: S6 Fig — a. The distribution of SIFT scores for the single nucleotide mutations observed in the selected ensembles of the laboratory drift, G17 (n = 1,957). The drift mutations were categorized according to their relative fitness effects (W rel; as in Fig 1). b. The same distribution after ‘nearly-neutral’ mutations were excluded: for the ‘deleterious’ mutations (W rel ≤0.6) and ‘neutral/beneficial’ (W rel >0.8). c. The correlation of W rel values with the SIFT scores. d. The correlation of W rel values with PROVEAN scores. (PDF) [file pcbi.1004421.s009.pdf]

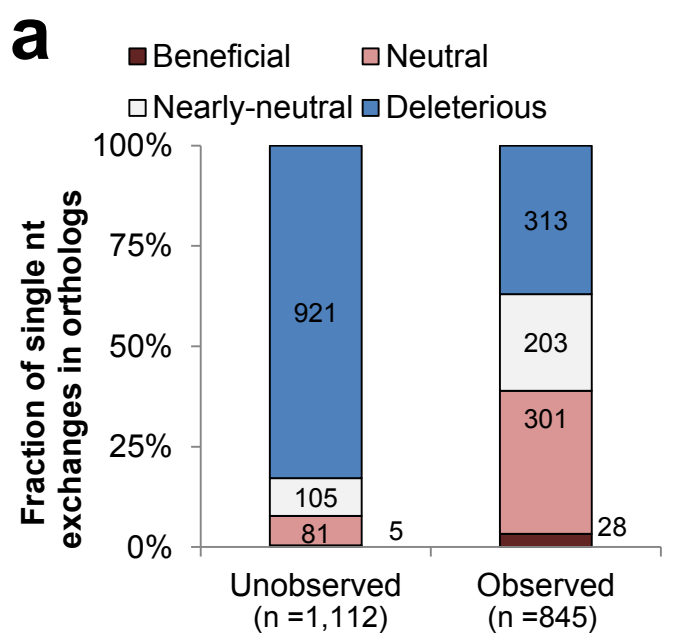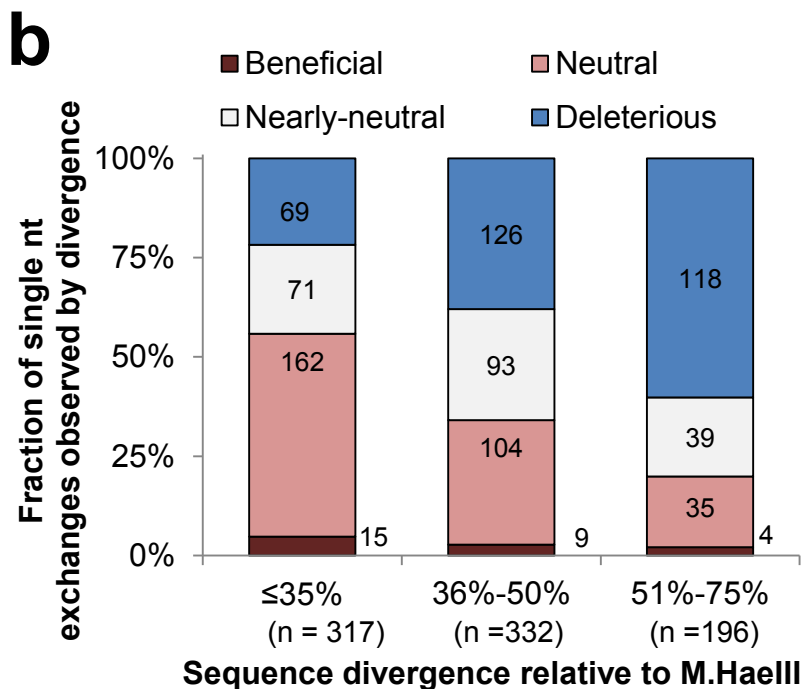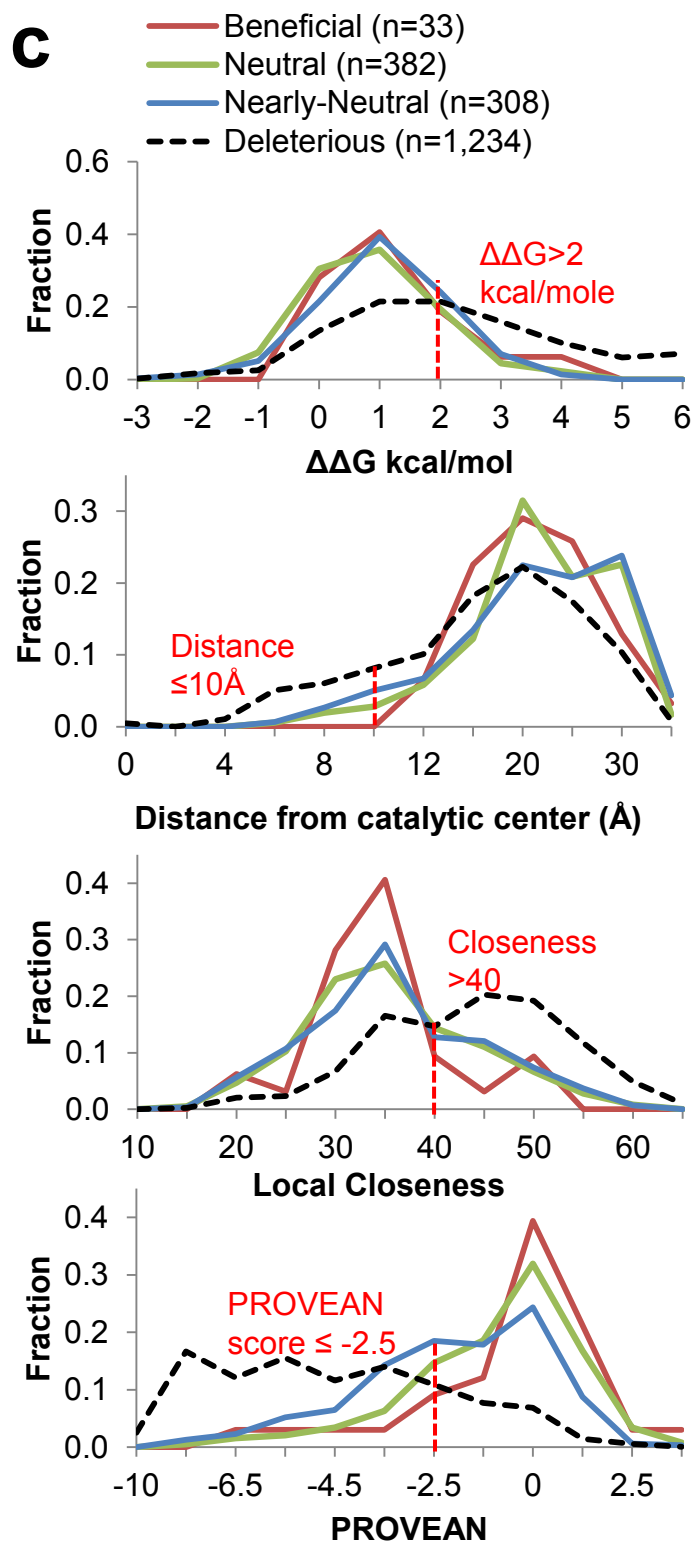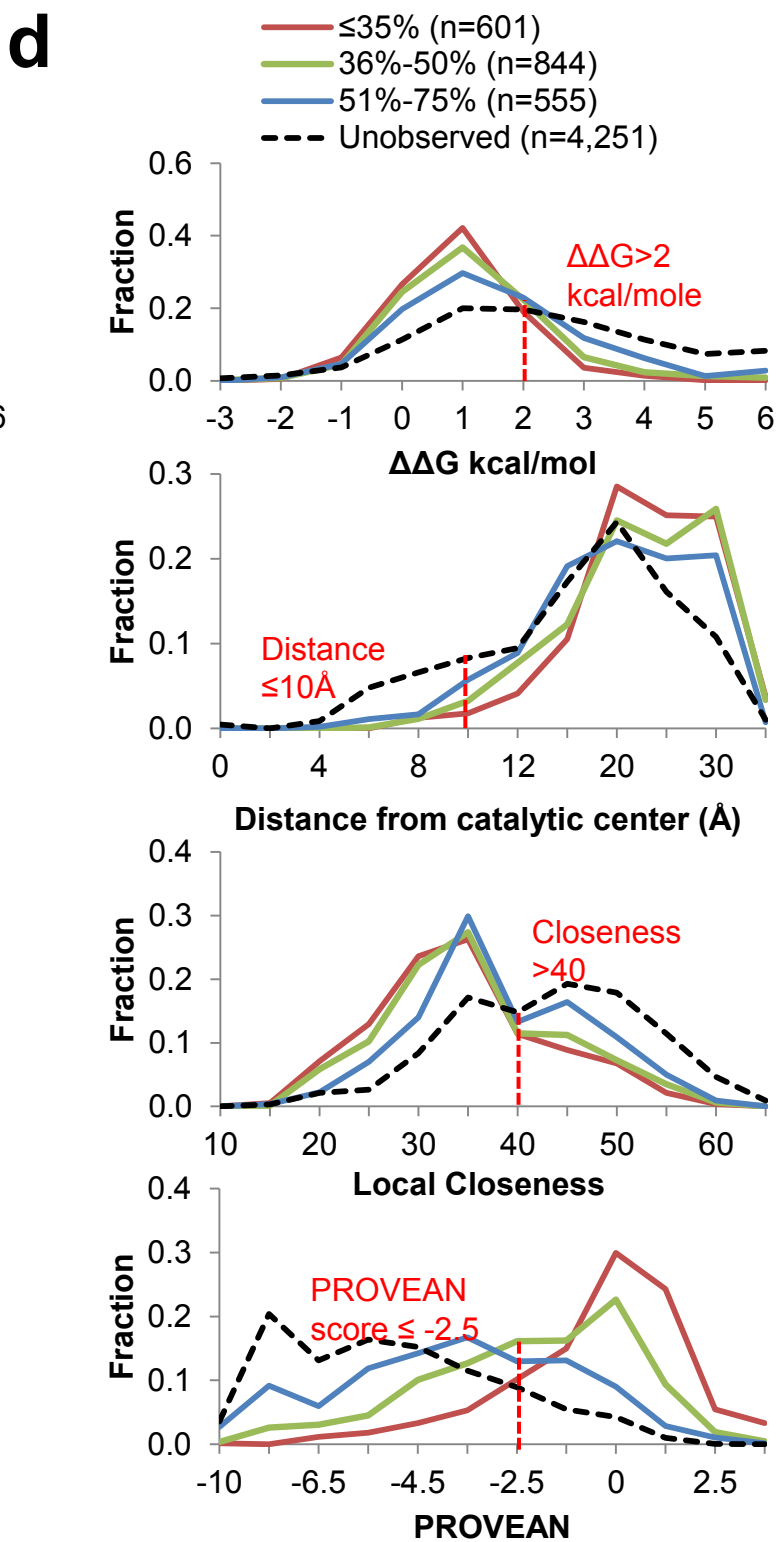

Supplement: S7 Fig — a. The single nucleotide mutational space (n = 1,957) was categorized according to whether the same mutation is seen in M.HaeIII orthologs (observed; n = 845), or not (unobserved; n = 1,112). Within each category, the exchanges were assigned as ‘beneficial’ (W rel > 1.1, oxblood), ‘Neutral’ (W rel > 0.8, ≤1.1, red), ‘Nearly-neutral’ (W rel > 0.6, ≤0.8, grey) or ‘Deleterious’ (W rel ≤0.6, blue) according to their relative fitness effects in the laboratory drift (W rel values in G17, as in Fig 1). b. The single nucleotide mutations were further divided according to their appearance in orthologs with different levels of sequence divergence relative to M.HaeIII (fraction of amino acids divergence of the closest ortholog in which a given mutation/exchange was found). c. The distributions of biophysical and functional constraints (as in Fig 4) and PROVEAN score (as in Fig 3) for the fractions of all the single nucleotide mutations according to their relative fitness effects in the laboratory drift (W rel values in G17, as in Fig 1). d. The distributions of biophysical and functional constraints (as in Fig 4) and PROVEAN score (as in Fig 3) for the fractions of all the ‘orthologs-observed’ exchanges (2,000 exchanges in total) with varying degrees of divergence, and for ‘ortholog-unobserved’ exchanges (4,251 exchanges). (PDF) [file pcbi.1004421.s010.pdf]

## G0 Single nt

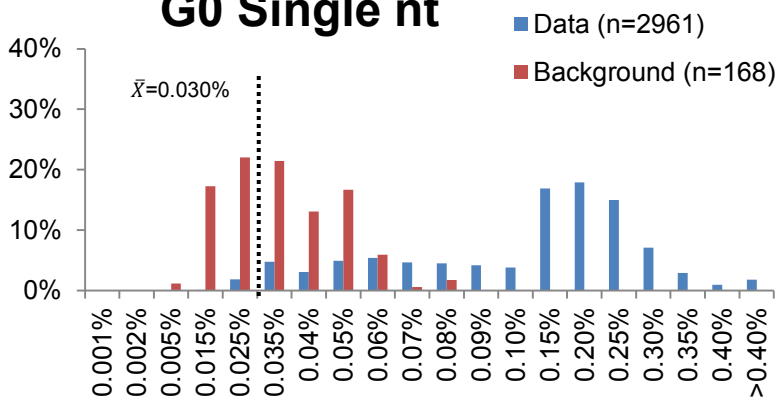

## G3 Single nt

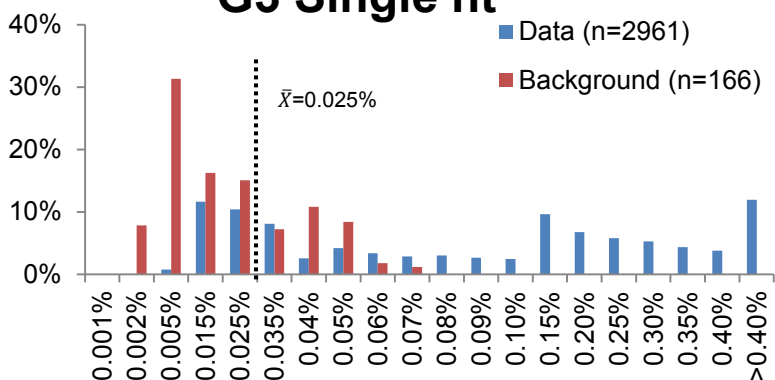

## G7 Single nt

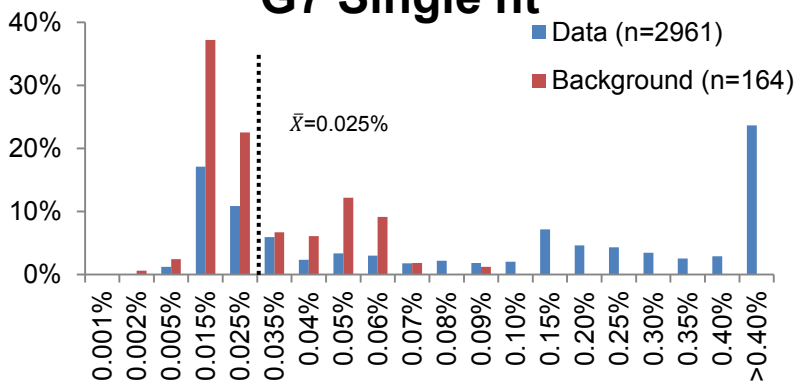

## G17 Single nt

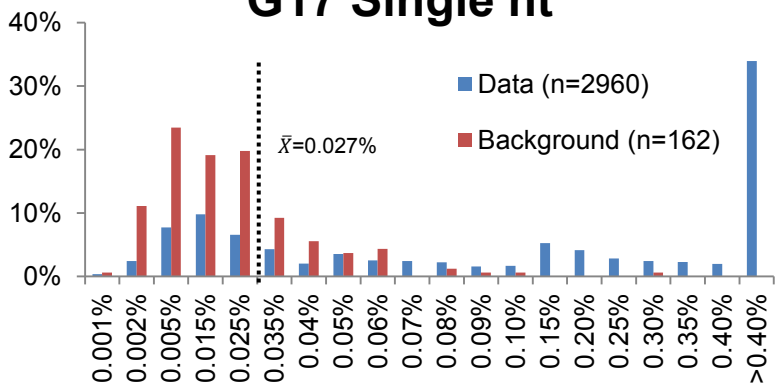

Supplement: S8 Fig — ‘Data’ relates to the distributions of the measured, raw mutation frequencies (i.e. prior to background subtraction) in each library within the coding region of M.HaeIII's (329 resides, in blue color, derived from S2 File). ‘Background’ relates to the distributions of the raw mutational frequencies in the region located upstream of the cloning sites, a region that was not subjected to mutagenesis (20 residues including His-tag and Thrombin cleavage site, residues -20 to -1, in red; S2 File). The average background frequency was subtracted from all measured frequencies, thus eliminating the effect of mutations that accumulated in the Illumina sequencing (S3 File and S4 Table). (PDF) [file pcbi.1004421.s011.pdf]
