# Supplementary material for: Systematic Mapping of Protein Mutational Space by Prolonged Drift Reveals the Deleterious Effects of Seemingly Neutral Mutations
Source: PLoS Comput Biol. 2015 Aug 14;11(8):e1004421. doi: 10.1371/journal.pcbi.1004421 (PMC4537296; doi:10.1371/journal.pcbi.1004421)
Supplement: S5 Fig — Orthologous sequences to M.HaeIII were collected using BLASTP search within the REBASE database [99]. Within the range of 25–75% identity, 105 non-redundant family members were identified and subsequently aligned using MUSCLE [100]. (PDF) [file pcbi.1004421.s008.pdf]

```
                                         10        20        30        40        50        60        70        80
                                ....|....|....|....|....|....|....|....|....|....|....|....|....|....|....|....|
MHaeIII stabilized reference    --------------------------------------------------------------------------------
tr|A8SMR4|A8SMR4_9FIRM Cytosin  --------------------------------------------------------------------------------
tr|G9QUQ3|G9QUQ3_9PROT Cytosin  --------------------------------------------------------------------------------
tr|C7HVB2|C7HVB2_9FIRM Cytosin  --------------------------------------------------------------------------------
tr|D1AWH4|D1AWH4_STRM9 Cytosin  --------------------------------------------------------------------------------
sp|P34906|MTF1_FUSNU Modificat  --------------------------------------------------------------------------------
tr|R7P745|R7P745_9CLOT Cytosin  --------------------------------------------------------------------------------
tr|T4PMD0|T4PMD0_CLODI Modific  --------------------------------------------------------------------------------
tr|I7IJB3|I7IJB3_9BURK Cytosin  --------------------------------------------------------------------------------
tr|F8KQQ2|F8KQQ2_HELBC Cytosin  --------------------------------------------------------------------------------
tr|G4QBU1|G4QBU1_TAYAM Cytosin  --------------------------------------------------------------------------------
tr|B0S4D0|B0S4D0_FINM2 Cytosin  --------------------------------------------------------------------------------
tr|R5X970|R5X970_9FUSO Cytosin  --------------------------------------------------------------------------------
UPI000372CBB5 status=active     --------------------------------------------------------------------------------
tr|U2TAM1|U2TAM1_9FUSO Unchara  --------------------------------------------------------------------------------
UPI00016C0771 status=active     --------------------------------------------------------------------------------
tr|A8MHW3|A8MHW3_ALKOO Cytosin  --------------------------------------------------------------------------------
tr|G8X6Y8|G8X6Y8_FLACA Cytosin  --------------------------------------------------------------------------------
tr|I4AHT3|I4AHT3_FLELS Cytosin  --------------------------------------------------------------------------------
tr|K2Q409|K2Q409_9FLAO Cytosin  --------------------------------------------------------------------------------
UPI0003695DD5 status=active     --------------------------------------------------------------------------------
UPI0002E32502 status=active     --------------------------------------------------------------------------------
tr|A6EU94|A6EU94_9BACT Cytosin  --------------------------------------------------------------------------------
tr|E8KGI3|E8KGI3_9PAST Cytosin  --------------------------------------------------------------------------------
tr|N9DBX7|N9DBX7_9GAMM Cytosin  --------------------------------------------------------------------------------
tr|K0C3B4|K0C3B4_CYCSP Cytosin  --------------------------------------------------------------------------------
tr|F9GXX4|F9GXX4_HAEHA Cytosin  --------------------------------------------------------------------------------
tr|D1K024|D1K024_9BACE Modific  --------------------------------------------------------------------------------
UPI0002E6F434 status=active     --------------------------------------------------------------------------------
tr|D5ERM7|D5ERM7_PRER2 Cytosin  --------------------------------------------------------------------------------
UPI0003ABCEDB status=active     --------------------------------------------------------------------------------
tr|L2F549|L2F549_9GAMM Cytosin  --------------------------------------------------------------------------------
tr|R6WUU4|R6WUU4_9CLOT Cytosin  --------------------------------------------------------------------------------
tr|C9PPY5|C9PPY5_9PAST Cytosin  --------------------------------------------------------------------------------
tr|K5TXT5|K5TXT5_9VIBR Cytosin  --------------------------------------------------------------------------------
tr|R5VXV7|R5VXV7_9FIRM Cytosin  --------------------------------------------------------------------------------
tr|C2MAC7|C2MAC7_9PORP Cytosin  --------------------------------------------------------------------------------
tr|R7G2J8|R7G2J8_9FIRM Cytosin  --------------------------------------------------------------------------------
tr|F0QZK5|F0QZK5_BACSH DNAcyto  --------------------------------------------------------------------------------
sp|P08455|MTP2_NEIGO Modificat  --------------------------------------------------------------------------------
tr|A5FP46|A5FP46_DEHSB Cytosin  --------------------------------------------------------------------------------
tr|D4WI15|D4WI15_BACOV DNA (Cy  --------------------------------------------------------------------------------
tr|E2PDE9|E2PDE9_NEIPO Cytosin  --------------------------------------------------------------------------------
tr|D2EGA8|D2EGA8_9EURY DNAcyto  --------------------------------------------------------------------------------
tr|D7FEK9|D7FEK9_HELP3 Cytosin  --------------------------------------------------------------------------------
tr|D1B3R7|D1B3R7_SULD5 DNAcyto  --------------------------------------------------------------------------------
tr|D6ZKT7|D6ZKT7_MOBCV Cytosin  --------------------------------------------------------------------------------
tr|K2EZH0|K2EZH0_9BACT Cytosin  --------------------------------------------------------------------------------
tr|F0S2I5|F0S2I5_DESTD DNAcyto  --------------------------------------------------------------------------------
tr|R7K6Q8|R7K6Q8_9FIRM Cytosin  --------------------------------------------------------------------------------
tr|R5R432|R5R432_9FIRM Cytosin  --------------------------------------------------------------------------------
tr|D8G3S4|D8G3S4_9CYAN Modific  --------------------------------------------------------------------------------
tr|D3I3G0|D3I3G0_9BACT Modific  --------------------------------------------------------------------------------
tr|E3D818|E3D818_GARV3 Cytosin  --------------------------------------------------------------------------------
UPI0001D09BA1 status=active     --------------------------------------------------------------------------------
UPI00030E071A status=active     --------------------------------------------------------------------------------
tr|F1XAT6|F1XAT6_MORCA Cytosin  --------------------------------------------------------------------------------
tr|I3CCM6|I3CCM6_9GAMM DNAmeth  --------------------------------------------------------------------------------
tr|B6XDR0|B6XDR0_9ENTR Cytosin  --------------------------------------------------------------------------------
tr|K9YXG2|K9YXG2_DACSA DNAmeth  --------------------------------------------------------------------------------
tr|D4XUU8|D4XUU8_ACIHA Cytosin  --------------------------------------------------------------------------------
tr|N8YEA4|N8YEA4_ACIBZ Cytosin  --------------------------------------------------------------------------------
tr|B4VSA7|B4VSA7_9CYAN C5 cyto  --------------------------------------------------------------------------------
sp|P29567|MTHT_METTF Modificat  --------------------------------------------------------------------------------
tr|L8N028|L8N028_9CYAN DNAcyto  --------------------------------------------------------------------------------
tr|K0JMW8|K0JMW8_BRAPL Cytosin  --------------------------------------------------------------------------------
tr|K9XTJ8|K9XTJ8_STAC7 DNAcyto  --------------------------------------------------------------------------------
UPI00036A774A status=active     --------------------------------------------------------------------------------
UPI0003826E4D status=active     --------------------------------------------------------------------------------
UPI00037126EF status=active     --------------------------------------------------------------------------------
tr|K9TTW7|K9TTW7_9CYAN DNAcyto  --------------------------------------------------------------------------------
tr|K9RM80|K9RM80_9CYAN DNAmeth  --------------------------------------------------------------------------------
tr|R7CTJ4|R7CTJ4_9BACE Cytosin  --------------------------------------------------------------------------------
tr|D4WI14|D4WI14_BACOV Cytosin  --------------------------------------------------------------------------------
tr|F0QZK6|F0QZK6_BACSH Cytosin  --------------------------------------------------------------------------------
tr|D1K023|D1K023_9BACE Cytosin  --------------------------------------------------------------------------------
UPI00037FAC0D status=active     --------------------------------------------------------------------------------
tr|R9HQV7|R9HQV7_BACVU Cytosin  --------------------------------------------------------------------------------
UPI0003717B77 status=active     --------------------------------------------------------------------------------
tr|K9VHM5|K9VHM5_9CYAN DNAcyto  --------------------------------------------------------------------------------
tr|T4JUF1|T4JUF1_CLODI Modific  --------------------------------------------------------------------------------
tr|S8B9I9|S8B9I9_CLOBO Cytosin  --------------------------------------------------------------------------------
tr|J0QZQ6|J0QZQ6_9RHIZ DNA (Cy  --------------------------------MMTCKINKIAERSSFDSSHELRSAHLLKPSTAITNALKSTMEDAADAL
tr|R5PBE9|R5PBE9_9BACT Cytosin  --------------------------------------------------------------------------------
tr|G6A2H7|G6A2H7_STRIT Cytosin  --------------------------------------------------------------------------------
tr|Q9K765|Q9K765_BACHD Cytosin  --------------------------------------------------------------------------------
tr|H1B1E9|H1B1E9_9FIRM Cytosin  --------------------------------------------------------------------------------
tr|A3DHU3|A3DHU3_CLOTH Cytosin  --------------------------------------------------------------------------------
tr|K4ZRS7|K4ZRS7_PAEAL Cytosin  --------------------------------------------------------------------------------
tr|F4PEX7|F4PEX7_BATDJ Cytosin  --------------------------------------------------------------------------------
tr|E5YPJ2|E5YPJ2_9BACL Cytosin  ----------------------------------------MKLNERGRGKFKPA-PNLSREEVQQVLFEKIQEEMSLIIE
```

```
sp|P13906|MTB1_LYSSH Modificat  --------------------------------------MAIKINEKGRGKFKPA-PTYEKEEVRQLLMEKINEEMEAVAT
tr|J7X9T9|J7X9T9_BACCE Cytosin  ---------------------------------------MVKIDERGRGKFKPA-PDYTQEQVRETLLQKIEEERQNKAD
tr|C7G547|C7G547_9FIRM Cytosin  --------------------------------------MAVILEERGRGKFKPA-PDYKVDEVKELLNAKIEEERQAFAD
sp|P06530|MTBR_BACIU Modificat  --------------------------------------MTLKIDIKGRGKYKPA-SDYSIDDVKNVLMEKIFEESSRIIN
tr|F0TJ68|F0TJ68_LACA3 Cytosin  ----------------------------------------MKKFESSLTFSAEKVKKILFAKIEEEKEKIENNKTEENVK
tr|B8DET5|B8DET5_LISMH Modific  -----------------------------------------MKNMITESKFTSS-STLSTQEIKKVLFSKIEDENKKIKN
tr|B1HWG5|B1HWG5_LYSSC Modific  --------------------------------------MEFKGKEKGRGKFKPA-PDLDRETVRNVLLNKISEEKALIID
tr|Q8GC38|Q8GC38_LEUCI Putativ  -------------------------------------------------MAFKSSSNLSKEKVKDVLFQKIKEEQSHIYD
tr|K4Z5B6|K4Z5B6_ENTFL DNAcyto  -------------------------MINMTQLVDESKVIHKSIEQRSRGKFKPEIRKLTNNQIKSVLLEKIAEEEVLINE
tr|S3P180|S3P180_9GAMM Cytosin  --------------------------------------------------------------------------------
tr|N9NU94|N9NU94_9GAMM Cytosin  --------------------------------------------------------------------------------
tr|E7GW62|E7GW62_STRAP Modific  ----------------------------------MKTTVIKDETKRSRGKFKPA-PNLSADEVKNVLYHKIEEETNKIIE
tr|I0QBF4|I0QBF4_STROR Modific  ----------------------------------MEEIIIKDDQKRSRGKFKPA-PNLTSDEVKNVLMQKIEEETNKIIE
tr|S4B3K9|S4B3K9_ENTCA Modific  MLKCFILVRTALIKRKEVSYMNNIKESKITPEVTDIITDIEIEKKFISSLTLDEATVQSVLKEKIDEENSIVLKNTTNEN
tr|S9C1L1|S9C1L1_STRAG Restric  ----------------------------------MEEIIIKDEQQRSRGKFKPA-PNLTTDEVKNVLLKKIEEETNKIIE

                                         90        100       110       120       130       140       150       160
                                ....|....|....|....|....|....|....|....|....|....|....|....|....|....|....|....|
MHaeIII stabilized reference    -----------------MNLISLFSGAGGLDLGFQKAG------------------------------FRIIAANEYDKS
tr|A8SMR4|A8SMR4_9FIRM Cytosin  ---------MILRRKYKMNLVSLFSGAGGLDLGFERAG------------------------------FNIVVANEYDKT
tr|G9QUQ3|G9QUQ3_9PROT Cytosin  -----------------MNLISLFSGAGGLDLGFEKAG------------------------------FKVVTANEYDKT
tr|C7HVB2|C7HVB2_9FIRM Cytosin  -----------------MKLISLFSGAGGLDLGFQKAG------------------------------YEIVAANEFDKT
tr|D1AWH4|D1AWH4_STRM9 Cytosin  -----------------MKIISLFSGAGGLDLGFKKAG------------------------------FDIVAANEFDKT
sp|P34906|MTF1_FUSNU Modificat  -----------------MKLLSLFSGAGGLDLGFERAG------------------------------FEIIVANEYDKT
tr|R7P745|R7P745_9CLOT Cytosin  ------------METKKFKILSLFAGAGGLDLGFEKAG------------------------------FDIVYANEYDKS
tr|T4PMD0|T4PMD0_CLODI Modific  -----------------MKLVSLFSGAGGLDLGFEKAG------------------------------FEIIMANEIDKT
tr|I7IJB3|I7IJB3_9BURK Cytosin  -----------------MKLISLFSGAGGLDLGFEQAG------------------------------YQIIAANEFDKS
tr|F8KQQ2|F8KQQ2_HELBC Cytosin  -----------------MNLISLFCGAGGLDLGFERAG------------------------------FKVVEANEIDSS
tr|G4QBU1|G4QBU1_TAYAM Cytosin  -----------------MKLISLFSGAGGLDLGFEQAG------------------------------YQIITANEHDKS
tr|B0S4D0|B0S4D0_FINM2 Cytosin  -----------------MDLISLFSGAGGLDLGFIKAG------------------------------FNVLVANEYDKR
tr|R5X970|R5X970_9FUSO Cytosin  -----------------MKLLSLFSGAGGLDLGFEKAG------------------------------FEIVAANEYDKT
UPI000372CBB5 status=active     -----------------MNLISLFSGAGGLDLGFEKAG------------------------------FDILAANEYDKT
tr|U2TAM1|U2TAM1_9FUSO Unchara  -----------------MNIISLFSGAGGMDLGFEKAG------------------------------FKILMANEIDKT
UPI00016C0771 status=active     -----------------MRLISLFAGAGGLDLGFEKAG------------------------------YEIAAANEYDKT
tr|A8MHW3|A8MHW3_ALKOO Cytosin  -----------------MKLISLFSGAGGMDLGFEKAG------------------------------FKVIAANEYDKT
tr|G8X6Y8|G8X6Y8_FLACA Cytosin  -----------------MKIVSFFAGAGGLDLGFQKAG------------------------------FNVIWANEYDKE
tr|I4AHT3|I4AHT3_FLELS Cytosin  -----------------MKIVSFFAGAGGLDLGFKKAG------------------------------FDIIWANEYDKE
tr|K2Q409|K2Q409_9FLAO Cytosin  -----------------MNLVSFFSGAGGLDLGFSKAG------------------------------FNVVWANEYDKD
UPI0003695DD5 status=active     -----------------MNVISLFAGAGGMDLGFQKAG------------------------------FDIVIANEYDKS
UPI0002E32502 status=active     ------------MTFSKPTIVALFAGCGGLDLGFRQAG------------------------------FNIIWANEYDKE
tr|A6EU94|A6EU94_9BACT Cytosin  -----------------MKIVSFFAGAGGLDLGFQQAG------------------------------FNVIWANEYDKE
tr|E8KGI3|E8KGI3_9PAST Cytosin  -----------------MKVISLFCGCGGLDLGFEKAG------------------------------FEVPVANEYDKT
tr|N9DBX7|N9DBX7_9GAMM Cytosin  -----------------MKVVSFFAGAGGLDLGFEQAG------------------------------FDVVWANEYDKE
tr|K0C3B4|K0C3B4_CYCSP Cytosin  -----------------MKIVSLFSGAGGLDLGFEKAG------------------------------FNVTWANEFDKD
tr|F9GXX4|F9GXX4_HAEHA Cytosin  -----------------MKVISLFCGCGGLDLGFERAG------------------------------FQVLVANEYDKT
tr|D1K024|D1K024_9BACE Modific  -----------------MELISLFSGAGGLDLGFHKAG------------------------------FRTVTANEFDAK
UPI0002E6F434 status=active     -----------------MKVVSFFAGAGGLDLGFENAG------------------------------FDVIWANEYDKE
tr|D5ERM7|D5ERM7_PRER2 Cytosin  -----------------MKVISLFSGCGGLDLGFERAG------------------------------FEVPVANEFDPS
UPI0003ABCEDB status=active     -----------------MKVISLFCGCGGLDLGFEKAG------------------------------FKIPVANEYDKT
tr|L2F549|L2F549_9GAMM Cytosin  ------------MSNYKPTLISLFAGAGGMDLGFMQAG------------------------------FEVILANEYDKK
tr|R6WUU4|R6WUU4_9CLOT Cytosin  -----------------MNLISLFSGCGGLDLGFEKAG------------------------------FNVAVANEFDPT
tr|C9PPY5|C9PPY5_9PAST Cytosin  -----------------MKVISLFSGCGGLDLGFEKAG------------------------------FEIPVANEYDKT
tr|K5TXT5|K5TXT5_9VIBR Cytosin  ---------------MTQRIVSFFAGAGGLDLGFEQAG------------------------------FNVTWANEYDKD
tr|R5VXV7|R5VXV7_9FIRM Cytosin  -----------------MNVISLFSGCGGLDLGFERAG------------------------------FNIPVANEFDKT
tr|C2MAC7|C2MAC7_9PORP Cytosin  ---------------MSMTLISLFSGAGGMDLGFHKAG------------------------------FTTILANEYDKT
tr|R7G2J8|R7G2J8_9FIRM Cytosin  -----------------MTILSLFSGCGGLDLGFEKAG------------------------------FEIPIANEYDKK
tr|F0QZK5|F0QZK5_BACSH DNAcyto  -----------------MTLISLFSGAGGLDLGFHYAG------------------------------FKTIIANELDAK
sp|P08455|MTP2_NEIGO Modificat  -----------------MKIISLFSGCGGLDLGFEKAG------------------------------FEIPAANEYDKT
tr|A5FP46|A5FP46_DEHSB Cytosin  -----------------MQIISLFSGAGGLDLGFEKAG------------------------------FNVVWANEYDKT
tr|D4WI15|D4WI15_BACOV DNA (Cy  -----------------MNLISLFSGAGGLDLGFHKAG------------------------------FKVVTANEFDAK
tr|E2PDE9|E2PDE9_NEIPO Cytosin  -----------------MKIISLFSGCGGLDLGFEKAG------------------------------FEVPVANEYDKT
tr|D2EGA8|D2EGA8_9EURY DNAcyto  ---------------MKCSLISLFSGCGGLDLGFRNAG------------------------------FGIVFANDNDKA
tr|D7FEK9|D7FEK9_HELP3 Cytosin  -----------------MNLLSLFAGAGGLDLGFEKAG------------------------------FKIVVANEYDKN
tr|D1B3R7|D1B3R7_SULD5 DNAcyto  -----------------MNIISLFSGAGGLDLGFEKAG------------------------------FKTVWANEYDKE
tr|D6ZKT7|D6ZKT7_MOBCV Cytosin  -----------------MNVISLFSGCGGLDLGFEQAG------------------------------FNIPVANEFDPT
tr|K2EZH0|K2EZH0_9BACT Cytosin  -----------------MKIASLFTGAGGLDLGFEKAG------------------------------FHVVWANEYDPT
tr|F0S2I5|F0S2I5_DESTD DNAcyto  -----------------MEIVSLFSGCGGLDLGFELAG------------------------------FSIVWANDNDKD
tr|R7K6Q8|R7K6Q8_9FIRM Cytosin  -----------------MKLISLFSGCGGLDLGFERAG------------------------------FDIPVANEYDPT
tr|R5R432|R5R432_9FIRM Cytosin  -----------------MNVISLFSGCGGLDLGFEKAG------------------------------FRIPVANEFDKS
tr|D8G3S4|D8G3S4_9CYAN Modific  -----------MFSDKDKSIVALFSGCGGLDLGFSQAG------------------------------FNVIWASEYDKD
tr|D3I3G0|D3I3G0_9BACT Modific  -----------------MRLISLFSGAGGLDKGFHNAG------------------------------FRTIVANEFDKK
tr|E3D818|E3D818_GARV3 Cytosin  -----------------MKVLSLFSGCGGLDLGFEKAG------------------------------FEIPVANEFDKT
UPI0001D09BA1 status=active     -----------------MKVISLFSGCGGLDLGFERAG------------------------------FEIPVANEYDKT
UPI00030E071A status=active     ---------------MEKAVVALFAGCGGLDLGFRKAG------------------------------FNVVWANEYDKD
tr|F1XAT6|F1XAT6_MORCA Cytosin  -----------------MKIISLFSGCGGLDLGFKKAG------------------------------FKIAVANEYDKS
tr|I3CCM6|I3CCM6_9GAMM DNAmeth  -----------------MKIISLFSGAGGLDLGFHQAG------------------------------FNTIWANEFDSS
tr|B6XDR0|B6XDR0_9ENTR Cytosin  ---------------MSNRIVSFFAGAGGLDLGFEQAG------------------------------FNVIWANEFDKD
tr|K9YXG2|K9YXG2_DACSA DNAmeth  -----------------MQIVSLFSGCGGLDLGFRQAG------------------------------FQVVWANEYDRT
tr|D4XUU8|D4XUU8_ACIHA Cytosin  ---------------MSLKIVSLFSGAGGLDLGFQKAG------------------------------FNITFANEFDKD
tr|N8YEA4|N8YEA4_ACIBZ Cytosin  ---------------MALKIASFFSGAGGLDLGFKQAG------------------------------FDIIFANEFDKD
tr|B4VSA7|B4VSA7_9CYAN C5 cyto  ---------MEQSNLKEKAIVSLFSGCGGLDLGFRQAG------------------------------FNVVWANEYDKD
sp|P29567|MTHT_METTF Modificat  ---------------MNMDIASFFSGAGGLDLGFTKAG------------------------------FNIVFANDNWKG
tr|L8N028|L8N028_9CYAN DNAcyto  ---------------MSKSVIALFSGCGGLDLGFRRAG------------------------------FNVTWANEYDKD
tr|K0JMW8|K0JMW8_BRAPL Cytosin  -------------MIKNLSIISLFTGAGGLDLGFKNAG------------------------------FKTIWANEYDKT
tr|K9XTJ8|K9XTJ8_STAC7 DNAcyto  --------MLSLNSINNLNIVSLFSGCGGLDLGFSKAG------------------------------FKIIWANDCDKE
UPI00036A774A status=active     -----------------MNIISLFSGCGGLDLGFKKAG------------------------------FNIPVANEFDKS
UPI0003826E4D status=active     -----------------MKIASFFSGAGGLDLGFSNAG------------------------------FELAYANDNWGG
UPI00037126EF status=active     ------MSSSLSNRKSSLNIVSLFAGCGGLDLGFTKAG------------------------------FTVNWANEWDKD
tr|K9TTW7|K9TTW7_9CYAN DNAcyto  -----------------MNVVSLFSGCGGLDLGFHQAG------------------------------FNIIWANEYDRS
tr|K9RM80|K9RM80_9CYAN DNAmeth  -----------------MNIVSLFSGCGGLDLGFEEAG------------------------------FNVIWANEYDKS
tr|R7CTJ4|R7CTJ4_9BACE Cytosin  -----------------MKVASFFAGCGGLDLGFEQAG------------------------------YEVIWADEFDET
tr|D4WI14|D4WI14_BACOV Cytosin  -----------------MKVASFFAGCGGLDLGFEQAG------------------------------YEVIWANEFDEG
tr|F0QZK6|F0QZK6_BACSH Cytosin  -----------------MKVASFFSGCGGLDLGFEQAG------------------------------IEVIWANDIEAS
tr|D1K023|D1K023_9BACE Cytosin  -----------------MKVASFFAGCGGLDLGFEQAG------------------------------YEVVWANEFDEA
UPI00037FAC0D status=active     -------------MLKDMKVASFFAGCGGLDLGFRQAG------------------------------YEVIWANEFDEA
```

```
tr|R9HQV7|R9HQV7_BACVU Cytosin  -------MKKGLSLLDDMKVASFFAGCGGLDLGFRQAG------------------------------YEVIWANEFDEA
UPI0003717B77 status=active     -----------MKYNNNFEIISLFAGCGGLDLGFEQAG------------------------------FNVIWANEYNKK
tr|K9VHM5|K9VHM5_9CYAN DNAcyto  ----MIYCIYCGLKKVALDIISLFCGCGGLDLGFRLAG------------------------------FNAVWANEYDKS
tr|T4JUF1|T4JUF1_CLODI Modific  ----------------MIRTVSLFAGCGGLDLGFKRAG------------------------------FNIIWANDNNKK
tr|S8B9I9|S8B9I9_CLOBO Cytosin  ----------------MLKTVALFAGCGGLDLGFENAG------------------------------FDIIWANDNNNK
tr|J0QZQ6|J0QZQ6_9RHIZ DNA (Cy  GKKKATVNPIKKNIKKRLSLVSLFAGAGGMDIGFEKAG------------------------------FKTVWANEYDKT
tr|R5PBE9|R5PBE9_9BACT Cytosin  -----------------MDVVSLFAGCGGLDLGFERAG------------------------------FNVKWANENDSS
tr|G6A2H7|G6A2H7_STRIT Cytosin  -----------------MNLISLFSGAGGLDLGFEKAG------------------------------FNVVAANEYDKT
tr|Q9K765|Q9K765_BACHD Cytosin  ------------MVKTNFTAVSLFSGAGGLDMGFERIG------------------------------FKIIWANDIDED
tr|H1B1E9|H1B1E9_9FIRM Cytosin  --------------MKHMTQASLFCGAGGLDIGFERAG------------------------------FKTVWANDFNKD
tr|A3DHU3|A3DHU3_CLOTH Cytosin  ---MKIIDLNKIRNIRKYTVASLFAGAGGLDMGLELAG------------------------------FKTVWANDIDKD
tr|K4ZRS7|K4ZRS7_PAEAL Cytosin  ------------MLDNKFTVASLFCGAGGLDLGFEVAG------------------------------FKTIWANDINKD
tr|F4PEX7|F4PEX7_BATDJ Cytosin  ----MLGYKFDMYSTVSLTVLGLFSGCGGLDLGLELAG--LAAAIGEEKALEAFKNKEKFDAIRGESIFHTIYSNDLFKE
tr|E5YPJ2|E5YPJ2_9BACL Cytosin  STDDEELLNNLDYKKDKFNLVSLFSGCGGLDMGFELAG--LAAVIGEEAAMEAFKDKDKFNEVRHKSIFHTIYTNDLFTE
sp|P13906|MTB1_LYSSH Modificat  ATSDISN-DEIQYKSDKFNVLSLFCGAGGLDLGFELAG--LEQSLGTDKALEAFKDRDVYNAIRHESVFHTVYANDIFSE
tr|J7X9T9|J7X9T9_BACCE Cytosin  THSDFTD-NELIYAKDKYNVTSLFCGAGGLDLGVELAG--LEAVVGTGQAIEAFQNKDAYNAIRHKSIFHKTYSNDFFKE
tr|C7G547|C7G547_9FIRM Cytosin  CSEEIEF-DKLNYDPNKWNLLSLFSGCGGLDLGFELAG--LKAVMGEEVMEAAFADKKVFDENINNNVFNTIYVNDIFDE
sp|P06530|MTBR_BACIU Modificat  SDDDLEIIEKVDFRTDKINVLSLFSGCGGLDLGFELAG--LAAVIGEQAAMEAFKDKDRFNELRNKSIFHTIYTNDLFKE
tr|F0TJ68|F0TJ68_LACA3 Cytosin  LDKELYDKASALYDDSKINVLSLFSGAGGLDLGVEIAA----ASAKHGVDKTYTAFQNRRELSKLLDSTNVIYSNDLFAS
tr|B8DET5|B8DET5_LISMH Modific  LSVPEIDYSNINYDNKKVNVVSLFSGAGGLDLGLELAG-VYAKKEQKEQPLELLNNYPRYKEIRKESLFNIIYSNDMFKE
tr|B1HWG5|B1HWG5_LYSSC Modific  DPKDLEVLKQVQYKSKKYNVVGLFSGCGGLDLGFELAG--LIAEIGEENAMTAFKNKEKFDEIRHKSVFNTIYTNDMFKE
tr|Q8GC38|Q8GC38_LEUCI Putativ  TNEDAEVISLVKQEDNKANVVSLFSGAGGLDLGLEMAG--IDAVMGSKFTDNILNDKTEYNKNRKKSIFNIVYSNDNFKE
tr|K4Z5B6|K4Z5B6_ENTFL DNAcyto  DPEDKLLLSDIALKRNKFNVVSLFSGAGGLDLGLELAG-LSSVIGESKAMESFFSSKQRYDKVREKSIFHTIYTNDMFKE
tr|S3P180|S3P180_9GAMM Cytosin  -----------------MKAVSLFSGCGGSDAGLINAG------------------------------FDVIMANDILPY
tr|N9NU94|N9NU94_9GAMM Cytosin  -----------------MKAVSLFSGCGGSDAGLIKAG------------------------------FEIIMANDILPY
tr|E7GW62|E7GW62_STRAP Modific  DSSDHELIQNADYQGDKINVVSLFSGAGGLDLGTELAG--LASVVGLQKALEIFEDKKEFSKKRHKSIFHTIYTNDMFVE
tr|I0QBF4|I0QBF4_STROR Modific  DSADYNLIQNADFRDDKINVVSLFSGAGGLDLGAEVAG--LSTKIGVVEALEALRDNKKFQAIRENGLFHTIYTNDMFVE
tr|S4B3K9|S4B3K9_ENTCA Modific  IKFEKELFSNINHINGAINVVSLFSGAGGLDLGVELANLVVEYGEKKAYQIFKDKRKFEKSLLKQNTRLNVIYTNDIFVE
tr|S9C1L1|S9C1L1_STRAG Restric  DSSDLKLVGECDFREDKINVVSLFSGAGGLDLGTELAG--LVSRIGITESFRAFEQREDFQDIREESLFHTIYTNDMFVE

                                         170       180       190       200       210       220       230       240
                                ....|....|....|....|....|....|....|....|....|....|....|....|....|....|....|....|
MHaeIII stabilized reference    IWKTYESNH-SAKLI-KGDISKISSDEFPK------CDGIIGGPPCQSWSEGGSLRGIDDPRGKLFYEYIRILKQKKPKF
tr|A8SMR4|A8SMR4_9FIRM Cytosin  IWETYEKNH-KTKLI-KGDICGIPSDMFPK------CDGIIGGPPCQSWSEAGSLKGIEDPRGQLFYQYIRILKDKQPRF
tr|G9QUQ3|G9QUQ3_9PROT Cytosin  IWETYEKNH-NTNLI-KGDICTIPSSKFPD------CDGIIGGPPCQSWSEAGSLKGIDDPRGQLFYQYIRILKDKQPKF
tr|C7HVB2|C7HVB2_9FIRM Cytosin  IWETYEKNH-EAKLI-KGDIFKIPSDEFPE------CDGIIGGPPCQSWSEAGSLKGINDPRGQLFYQYIRILKDKKPKF
tr|D1AWH4|D1AWH4_STRM9 Cytosin  IWETYEKNH-KTHLI-KGDICNIHSSMFPE------CDGIIGGPPCQSWSEAGSLKGIEDPRGQLFYQYIRILKEKQPKF
sp|P34906|MTF1_FUSNU Modificat  IWETYEKNH-KAKLI-KKDIREILSEELPK------SDGIIGGPPCQSWSEAGSLRGINDPRGKLFYEYIRILKDIQPKF
tr|R7P745|R7P745_9CLOT Cytosin  IWETYEKNH-KTPLD-HRDIRKISSDELPD------CDGIIGGPPCQSWSEAGSLRGINDSRGQLFYDFIRILKDKKPKF
tr|T4PMD0|T4PMD0_CLODI Modific  IWDTYEKNH-TAKLL-KGDISKIASREFPE------CDGIIGGPPCQSWSEAGSLRGINDPRGQLFYEYIRILKDKQPKF
tr|I7IJB3|I7IJB3_9BURK Cytosin  IWKTYEDNH-NAPLL-KGDISKILSSEFPD------CDGIIGGPPCQSWSEAGSLKGIDDPRGMLFYEYIRILKDKQPLF
tr|F8KQQ2|F8KQQ2_HELBC Cytosin  IWQTYENNH-RAKLI-RGDICKLGSKAFAP------CDGIIGGPPCQSWSEAGTLRGINDPRGQLFYEYIRVLKDKKPLF
tr|G4QBU1|G4QBU1_TAYAM Cytosin  IWKTYEQNH-DVPLL-KGDISTIPSSEFPN------CDGIIGGPPCQSWSEAGSLKGIDDPRGLLFYEYIRILKDKQPMF
tr|B0S4D0|B0S4D0_FINM2 Cytosin  IWETYEKNY-ETRLI-KDDITNVRSNELPD------CDGLIGGPPCQSWSEAGSLKGIEDPRGQLFYEYIRILRDKSPKF
tr|R5X970|R5X970_9FUSO Cytosin  IWETYEKNH-KAKLI-KKDIREIVSNELPK------TDGIIGGPPCQSWSEAGSLRGIDDPRGKLFYEYIRILKDKQPKF
UPI000372CBB5 status=active     IWETYEKNH-KAHLI-KKDIAKVSLDEFPN-----NIEGIIGGPPCQSWSVGGSLRGIDDPRGKLFYEYIRILKDKKPLF
tr|U2TAM1|U2TAM1_9FUSO Unchara  IWSTYEKNH-KIPII-KKDIRKVDSKEFPD------CDGIIGGPPCQSWSEAGALRGIEDDRGKLFFDYIRILREKKPKF
UPI00016C0771 status=active     IWATYEKNH-KGPLI-KGDISKIPSADFPD-----GIDGIIGGPPCQSWSAAGRLRGIEDPRGRLFYEYIRILKDKKPKF
tr|A8MHW3|A8MHW3_ALKOO Cytosin  IWETYEKNH-NAPLI-KGDIREIASGDFPD-----DCDGIIGGPPCQSWSEAGALRGIEDSRGQLFYEYIRLLRDKQPKF
tr|G8X6Y8|G8X6Y8_FLACA Cytosin  IWETYEKNHPHTKLD-RRSIVNIPADEVPD------CDGIIGGPPCQSWSEAGALKGINDKRGQLFFDFIRILEAKQPKF
tr|I4AHT3|I4AHT3_FLELS Cytosin  IWETYQKNHSQTILD-KRSIVDIATNEVPD------CDGIIGGPPCQSWSEAGKARGIKDKRGQLFYDFIRILEEKQPKF
tr|K2Q409|K2Q409_9FLAO Cytosin  IWKTYEENHLDTFLD-KRSIVDILSEEVPQ------CDGIIGGPPCQSWSEAGSLRGIQDKRGQLFFDFIRILADKQPKF
UPI0003695DD5 status=active     IWATYEKNH-QNPLL-KGDIRKFSADDFPD------CDGIIGGPPCQSWSEAGSLRGIDDERGQLFFDYIRVLKSKQPKF
UPI0002E32502 status=active     IWSTYEKNHPQNFLD-RRDIRKIPSAEIPN------CTGIIGGPPCQSWSEGGCQRGIEDERGQLFLEYIRILQDKQPQF
tr|A6EU94|A6EU94_9BACT Cytosin  IWETYEKNHPNTILD-KRSIVNIPADEVPE------CDGIIGGPPCQSWSEAGAARGIKDKRGQLFYDFIRILEAKQPKF
tr|E8KGI3|E8KGI3_9PAST Cytosin  IWATFKANHPKTRLI-EGDIRNIKEEDFPD-----EIDGIIGGPPCQSWSEAGALRGIDDARGQLFFDYIRILKSKQPKF
tr|N9DBX7|N9DBX7_9GAMM Cytosin  IWSTYELNHKNTILD-KRSIVDISSDEVPD------CDGIIGGPPCQSWSEAGSKRGITDKRGQLFYEFMRILADKKPKF
tr|K0C3B4|K0C3B4_CYCSP Cytosin  IWETYEKNFPHTELN-KKSIRDVELDEIPE------CDGIIGGPPCQSWSEAGKLRGIKDKRGQLFFEFIRILRDKKPKF
tr|F9GXX4|F9GXX4_HAEHA Cytosin  IWETFKANHPKTRLI-EGDIRNIKEDDFPD-----EVDGIIGGPPCQSWSEAGALRGINDARGQLFFDYIRILKSKQPKF
tr|D1K024|D1K024_9BACE Modific  ICPTFRANFPEVNLI-EGDIRDIPSCEFPD-----NITGIIGGPPCQSWSEAGSLKGIEDARGQLFYEYIRILRDKQPLF
UPI0002E6F434 status=active     IWETFEKNHKNTVLD-RRSIVDIPSNEVPD------CDGIIGGPPCQSWSEAGSKRGIADKRGQLFYEFMRILADKKPKF
tr|D5ERM7|D5ERM7_PRER2 Cytosin  IWETFEANHPKTKLI-RGDIRNIKESDFPK-----DVDGIIGGPPCQSWSEAGSLRGIEDARGQLFFDYIRILQEVKPKF
UPI0003ABCEDB status=active     IWATFKANHPNTRLI-EGDIRDIKEEDFPD-----NIDGIIGGPPCQSWSEAGSLRGIDDARGQLFFDYIRILKSKQPKF
tr|L2F549|L2F549_9GAMM Cytosin  IWATYEYNH-QTPLL-KGDIRHFNADDFPN------CDGIIGGPPCQSWSEAGALRGIDDARGQLFFDYIRILTAKQPKF
tr|R6WUU4|R6WUU4_9CLOT Cytosin  IWATFKVNHPNTKLI-EGDIRKIKDTDFPD-----NIDGIIGGPPCQSWSEAGAQRGIDDLRGQLFYEYIRILKSKQPKF
tr|C9PPY5|C9PPY5_9PAST Cytosin  IWATFKANHPKTKLI-EGDIRSIKEDDFPN-----EIDGIIGGPPCQSWSEAGSLRGINDARGQLFFDYIRILKSKQPKF
tr|K5TXT5|K5TXT5_9VIBR Cytosin  IWATYEKNHPHTTLD-RRSITNIDADEVPE------CDGIIGGPPCQSWSEAGAQRGIEDKRGQLFFDFIRILEAKKPKF
tr|R5VXV7|R5VXV7_9FIRM Cytosin  IWATFKANHPKTKLI-EGDVRKVTKEDIAQ-YIDGEVDGIIGGPPCQSWSEAGALRGIEDERGQLFFDYIRILKEFKPKF
tr|C2MAC7|C2MAC7_9PORP Cytosin  ICPTFKHNFPDVPLL-EGDIRKIPERLFPR-----HIDGIIGGPPCQSWSEAGALKGIEDARGQLFFDYIRILKYTQPKF
tr|R7G2J8|R7G2J8_9FIRM Cytosin  IYETFKVNHPNTHLI-EGDIRNIAEDDFPD-----EIDGIIGGPPCQSWSEAGSLRGIDDARGQLFYEYIRILRKKQPKF
tr|F0QZK5|F0QZK5_BACSH DNAcyto  ICPTYRINFPDVKLI-EGDIRNIPSSEFPD-----GITGIIGGPPCQSWSEAGSLKGIEDARGQLFYEYIRILKDKQPLF
sp|P08455|MTP2_NEIGO Modificat  IWATFKANHPKTHLI-EGDIRKIKEEDFPE-----EIDGIIGGPPCQSWSEAGALRGIDDARGQLFFDYIRILKSKQPKF
tr|A5FP46|A5FP46_DEHSB Cytosin  IWDTYQHNFPNTNLD-TRSITEIPSTEIPT------SDGIIGGPPCQSWSEAGALRGINDHRGQLFFEYIRILKDKQPSF
tr|D4WI15|D4WI15_BACOV DNA (Cy  ICPTFRANFSDTNLI-EGDIRDIPSSEFPD-----NIAGIIGGPPCQSWSEAGSLKGIEDARGQLFYEYIRILHDKQPLF
tr|E2PDE9|E2PDE9_NEIPO Cytosin  IWSTFKANHPKTHLI-EGDIRSIKEEDFPD-----EIDGIIGGPPCQSWSEAGALRGIDDERGQLFFDYIRILKSKQPKF
tr|D2EGA8|D2EGA8_9EURY DNAcyto  VWETYEKNF-GHKLD-GRSIIDIKSEDLPD------ADGIIGGPPCQSWSLAGAMRGINDERGQLFYEYIRVIRDKQPKF
tr|D7FEK9|D7FEK9_HELP3 Cytosin  ITPTYRLNHKNTQLL-EKDIKNLQTSEINF-----SVDGIIGGPPCQSWSEAGNLKGIDDARGQLFYEYLRLLKELKPKF
tr|D1B3R7|D1B3R7_SULD5 DNAcyto  IWETFEKNFPHTTLD-RRSIRNIPSCDIPE------AIGLIGGPPCQSWSEAGALRGIEDHRGQLFFEFIRVLRDKKPLF
tr|D6ZKT7|D6ZKT7_MOBCV Cytosin  IYETFKANHPHTHLI-EGDIRGVTIEDIAP-FVDGEVDGIIGGPPCQSWSEAGALRGIEDARGQLFYDYIRILREFRPKF
tr|K2EZH0|K2EZH0_9BACT Cytosin  IWETFEYNFPKTKLD-KRSIVDVPASEIPD------VDGIIGGPPCQSWSEAGAGRGINDKRGKLFYDYIRLLKEKQPKF
tr|F0S2I5|F0S2I5_DESTD DNAcyto  VWETYTRNFPGTYLD-KRDLRVIPVSDIPD------CVGIIGGPPCQSWSEAGNRKGIEDERGRLVLNYIDIISRKRPYF
tr|R7K6Q8|R7K6Q8_9FIRM Cytosin  IWETYRVNHPSTKLI-EGDIRNIKESDFPN-----DVDGIIGGPPCQSWSEAGSLRGINDSRGKLFYDYIRILKSKQPKF
tr|R5R432|R5R432_9FIRM Cytosin  IWETFKINHPKSHLI-EGDIRQVKKEDLAR-YIDGNVDGIIGGPPCQSWSEAGSLRGIEDARGQLFYDYIRILKEFTPRF
tr|D8G3S4|D8G3S4_9CYAN Modific  IWETYENNHPDTFLD-KRDIRAISSAEIPD------CTGIIGGSPCQSWSEAGMQRGIDDYRGKLFLDYVRILKEKQPLF
tr|D3I3G0|D3I3G0_9BACT Modific  ICPTFRANFPDTKLI-EGDIHDIPSDAFPM-----NPVGIIGGPPCQSWSEAGTLKGIEDARGQLFYEYIRILRDTQPLF
tr|E3D818|E3D818_GARV3 Cytosin  IWDTFKINHPKTHLI-EGDVRKVSKNDISE-YLSGNLDGIIGGPPCQSWSEAGSLRGIDDDRGKLFFDYIRILKDFKPKF
UPI0001D09BA1 status=active     IWATYKANHPKTQLI-EGDIRHIQEINFPD-----EIDGIIGGPPCQSWSEAGALRGIDDARGQLFFDYIRILKSKQPKF
UPI00030E071A status=active     IWETYEKNHLNTVID-RRDIREISSKEIPD------CVGIIGGPPCQSWSEAGSQRGINDERGKLFLEYVRILKDKQPLF
tr|F1XAT6|F1XAT6_MORCA Cytosin  IWATFKANHHNTKLI-EGDIRHILEEDFPN-----DIDGIIGGPPCQSWSEAGALRGINDARGQLFYDYIRILKNKQPKF
tr|I3CCM6|I3CCM6_9GAMM DNAmeth  IWETFEYNFPHVHLD-RRSITDILPTDIPE------CAGIIGGPPCQSWSEAGARRGINDKRGQLFFEYIRILKEKSPLF
tr|B6XDR0|B6XDR0_9ENTR Cytosin  IWATYKKNHPHTELD-QRSITNITSDEVPE------CDGIIGGPPCQSWSEAGAQRGINDKRGQLFFDFIRILEAKKPKF
tr|K9YXG2|K9YXG2_DACSA DNAmeth  IWSTYQRNHQNTILD-QRDIKEITSQDIPD------CVGIIGGPPCQSWSEGGKQRGINDDRGRLFLEYIRVLKDKQPDF
tr|D4XUU8|D4XUU8_ACIHA Cytosin  IWATYEYNHPNTHLD-KRSITEIKENEVPE------CDGIIGGPPCQSWSEGGARRGIDDKRGQLFFDFIRILKAKKPSF
tr|N8YEA4|N8YEA4_ACIBZ Cytosin  IWATYEHNHPETTLD-KRSITLIDEAEVPD------CDGIIGGPPCQSWSEAGSKRGIEDKRGQLFFDFIRILEAKKPAF
tr|B4VSA7|B4VSA7_9CYAN C5 cyto  IWQTYQSNHPNTFLD-RRDIRHVPSADIPD------CFGIVGGPPCQSWSEAGTQKGINDDRGRLFLEYIRILKDKQPRF
```

```
sp|P29567|MTHT_METTF Modificat  CWKTFEKNH-GIKIN-KKPIEWLKPSEIPD------VVGFIGGPPCQSWSLAGSMCGADDPRGKTFYAYVDLVKEKDPLF
tr|L8N028|L8N028_9CYAN DNAcyto  IWETYERNHVDTVLD-RRDIRKIASEEIPD------CLGIIGGPPCQSWSEGGTQRGIDDARGQLFLDYIRILRDKQPRF
tr|K0JMW8|K0JMW8_BRAPL Cytosin  IWDTFKYNFPETRLD-TRNITEIDSKDIPN------ASGLIGGPPCQSWSEAGAGRGINDDRGKLFYDYIRVLKDKQPNF
tr|K9XTJ8|K9XTJ8_STAC7 DNAcyto  IWQTYQYNHPKTYLD-RRDIRVIPSGDIPD------CFGIIGGPPCQSWSEAGSQRGIQDSRGQLFLEYIRILEDKQPLF
UPI00036A774A status=active     IFETFKVNHPDTHLI-EDDIRKITFEDILPFVAMDKVDGIIGGPPCQSWSEAGSLKGIEDVRGQLFFDYIRILKDFKPKF
UPI0003826E4D status=active     CWETFEKNH-RIKID-KRSIVDVKPEEIPN------VVGFVGGPPCQSWSLAGAMKGINDPRGKLFWNYVELIEKKQPLF
UPI00037126EF status=active     IWQTYELNHPNTYLD-RRDIRQILSLDIPD------CIGIVGGPPCQSWSEAGRQKGIQDDRGRLFLEYIRILRDKQPLF
tr|K9TTW7|K9TTW7_9CYAN DNAcyto  IWNTYEINHQNTKLD-KRDIRSIQSDEIPD------CIGIIGGPPCQSWSEAGAGRGINDSRGQVFYEYIRILKDKKPLF
tr|K9RM80|K9RM80_9CYAN DNAmeth  IWDTYEFNHPNTQLD-KRDIRKIVPDDIPD------CIGIIGGPPCQSWSEAGAQRGIDDSRGRLFFDYIRILKEKKPLF
tr|R7CTJ4|R7CTJ4_9BACE Cytosin  IHKTYQFNHPNTYLC-KADIRELKGEDIPE------CDGFIGGPPCQAWSEGGKQLGLEDERGLLFLEYIRLIKEKLPTF
tr|D4WI14|D4WI14_BACOV Cytosin  IHETYQFNHPNTYLC-KSDIRTLKAADIPD------CDGFIGGPPCQSWSEGGKQLGLNDDRGRLFLDYIQLIKQKRPKF
tr|F0QZK6|F0QZK6_BACSH Cytosin  IHETYQYNHPNTILC-KSDIRELHASDIPD------CDGFIGGPPCQSWSEGGKQLGLNDERGKLFLDYVRLIKEKQPKF
tr|D1K023|D1K023_9BACE Cytosin  IHKTYQFNHPNTYLC-KSDIRKLKGEDIPD------CDGFIGGPPCQSWSEGGRQLGLDDERGRLFFDYVRLIKEKHPKF
UPI00037FAC0D status=active     IHKTYQFNHPNTCLC-KSDIRTLKAADIPD------CDGFIGGPPCQSWSEGGKQLGLEDERGQLFFDYIRLIKEKCPKF
tr|R9HQV7|R9HQV7_BACVU Cytosin  IHKTYQFNHPNTFLC-KSDIRTLKATDIPD------CDGFIGGPPCQSWSEGGRQLGLEDERGKLFFDYIRLIKEKRPKF
UPI0003717B77 status=active     IWATYEENHKQTELD-KRSIVDIESEEIPK------ATGIIGGPPCQSWSLAGSMGGIDDNRGKLFYEYIRILRDKQPEF
tr|K9VHM5|K9VHM5_9CYAN DNAcyto  IWDTYNFNHPETILD-RRDIRKIKSDEIPN------CIGIIGGPPCQSWSEAGAKRGIDDSRGQLFWEYIRIVRDKQPLF
tr|T4JUF1|T4JUF1_CLODI Modific  IEETYKANHSNTKLV-IKSIVDLEEEEIPE------GDVIIGGPPCQSWSYAGSGKGKEDSRGKLFYEYINIIKHKKPKA
tr|S8B9I9|S8B9I9_CLOBO Cytosin  VADTYRYNHKNTKLI-IDSIVNINSDDIPD------CDIIIGGPPCQSWSLAGAMKGKEDSRGQLFYEYVRIIKDKRPKA
tr|J0QZQ6|J0QZQ6_9RHIZ DNA (Cy  ISPSYQKYFPKAKFD-SRSICDIPDTDLPE-----NITGVIGGPPCQSWSEAGARRGIDDPRGKLIFEYLRVIKRTKPIF
tr|R5PBE9|R5PBE9_9BACT Cytosin  VFETYRRNHLNTILN-TTDVRLLKDQDIPS------CDGVIGGPPCQSWSEGGKQLGLEDPRGKLLLEYVRIVKMKKPKF
tr|G6A2H7|G6A2H7_STRIT Cytosin  IWETYEKNH-DTKLI-KGDICGIPSEMFPE------CDGIIGGPPCQSWSEAGSLKGIDDPRGQLFYQYIRILKDKKPKF
tr|Q9K765|Q9K765_BACHD Cytosin  ACATHRLWS-DAEVV-QGDISKIDVSTIPS------ADILLGGWPCQGFSLAGP-RQLDDSRNTLYKYYVKYLEEKKPLA
tr|H1B1E9|H1B1E9_9FIRM Cytosin  ACDTFSRWSDATVVC--GDIGKVSEDDIPD------TDIILGGFPCQGFSLSGP-RKLDDSRNSLYKHYVRIVKAKRPLA
tr|A3DHU3|A3DHU3_CLOTH Cytosin  ACATYRLWS-QADVV-QGDIAKIDYSDVPD------TDVITGGFPCQGFSLAGP-RKINDERNKLYRYFVKLVELKQPYA
tr|K4ZRS7|K4ZRS7_PAEAL Cytosin  ACETHKQWS-DAEVI-SKDIKDISFSDVPS------VEVILGGFPCQGFSLAGP-RKIDDKRNSLYSFFAKLVGEKQPLA
tr|F4PEX7|F4PEX7_BATDJ Cytosin  ANESYKLNFPSTFQH-ELDIRKI--KEFPN------ADLVLGGFPCPGFSEAGP-RLIDDERNFLYIHFIRCLLQAKPFA
tr|E5YPJ2|E5YPJ2_9BACL Cytosin  ANESYKINFPQSVVQHRKDIRKV--KNFPK------ADVVVGGFPCPGFSEAGP-RLIDDERNFLYIHFIRCLIETQPYF
sp|P13906|MTB1_LYSSH Modificat  ALQTYEKNMPNHVFIHEKDIRKI--KEFPS------ANLVIGGFPCPGFSEAGP-RLVDDERNFLYIHFIRCLMQVQPEI
tr|J7X9T9|J7X9T9_BACCE Cytosin  ALQTYKKNSPQDVYLHNKDIRKI--HTFPN------ADLVLGGFPCPGFSEAGP-RLVDDERNFLYLHFIRCLMQTQPKF
tr|C7G547|C7G547_9FIRM Cytosin  ARETYAQNAGKYIYMDKSDIRKI--KEFPK------ADIVLGGFPCPGFSEAGP-RLVDDKRNFLYLHFIRCLMQSKPKI
sp|P06530|MTBR_BACIU Modificat  ANQTYKTNFPGHVIQHEKDIRQV--KYFPK------CNLILGGFPCPGFSEAGP-RLIDDDRNFLYLHFIRSLIQAQPEI
tr|F0TJ68|F0TJ68_LACA3 Cytosin  ANATYKDNFSGNYVKDTRDVRKV--ISFPK------ADLVLGGFPCPGFSVAGP-RLLDDPRNFLYIHYIRALMDSTPKI
tr|B8DET5|B8DET5_LISMH Modific  ANETYLANFQSNILKQELDIRKIP--NFPK------CELMIGGFPCPGFSAAGP-RLIDDERNFLYIHFIRALMQSQPDF
tr|B1HWG5|B1HWG5_LYSSC Modific  ANETYKLNFPDHIIQHEKDIRKV--ANFPM------CNLMLGGFPCPGFSEAGP-RLIDDERNFLYIHFIRALMQTQPEF
tr|Q8GC38|Q8GC38_LEUCI Putativ  ANQTYTDMFSENIVKHDKDIRKV--AIFPN------NDIMIGGFPCPGFSSAGP-RLIDDPRNFLYVHFIRALVETQPAF
tr|K4Z5B6|K4Z5B6_ENTFL DNAcyto  ANESYKLNFPSHVTQHKKDIRRV--AKFPN------CQIMIGGFPCPGFSDAGP-RLIDDPRNFLYIHFIRALTQTQPAF
tr|S3P180|S3P180_9GAMM Cytosin  AKEVYEANHPETDYI-LQSITEL--EHFPS------ADILAGCYPCQGFSQGGK-RQADNKLNYLYKEFARALNYIKPKG
tr|N9NU94|N9NU94_9GAMM Cytosin  AKEVYEANHPETDYL-LQNIETV--ENFPA------ADILAGCYPCQGFSQGGQ-RKADNKLNYLYLEFARALNLIRPKA
tr|E7GW62|E7GW62_STRAP Modific  ANETYKKNFPPNIIQHQKDIRKV--AHFPN------NELTVGGFPCPGFSEAGP-RLIDDERNFLYIHFIRELMQTQPAF
tr|I0QBF4|I0QBF4_STROR Modific  ANETYKNNFSSNIIQHQKDIRKV--AHFPK------NDLTIGGFPCPGFSEAGP-RLIDDERNFLYIHFIRELIQTQPLF
tr|S4B3K9|S4B3K9_ENTCA Modific  ANQSYRANFMNTVVSDNRDIRKV--ANFPK------SNLMLGGFPCPGFSAAGP-RLLDDPRNFLYIHYIRALMQSNPAF
tr|S9C1L1|S9C1L1_STRAG Restric  ANATYRRNFSPNIIQHQKDIRKV--AHFPK------NDLTIGGFPCPGFSEAGP-RLIDDDRNFLYIHFIRELIQTQPSF

                                         250       260       270       280       290       300       310       320
                                ....|....|....|....|....|....|....|....|....|....|....|....|....|....|....|....|
MHaeIII stabilized reference    FLAENVKGMLAQRHNKAVQEFIQEFDNA--GYDVHIILLNANDYGVAQDRKRVFYIGFRKE-----LNI-NYL-PPIP--
tr|A8SMR4|A8SMR4_9FIRM Cytosin  FLAENVKGMMAKRHNDAVENIVSQFEEA--GYDVFIHLLNASDYGVPQDRKRVFYVGFRKD-----LKV-KFE-PPKP--
tr|G9QUQ3|G9QUQ3_9PROT Cytosin  FLAENVKGMMAKRHNSAVKNIISQFEEA--GYDVFIHLLNASDYGVPQDRKRVFYVGFRKD-----LNI-DFKNPPKP--
tr|C7HVB2|C7HVB2_9FIRM Cytosin  FLAENVKGMMAKRHNSAVENIVSQFEEA--GYDVFIHLLNASDYGVPQDRKRVFYVGFRKD-----LNI-SFD-LP-P--
tr|D1AWH4|D1AWH4_STRM9 Cytosin  FLAENVKGMMSKRHNDAVKNIVSQFEEA--GYDVYIHLLNASDYGVPQDRKRVFYIGFRKD-----LNI-KFD-KP-P--
sp|P34906|MTF1_FUSNU Modificat  FLAENVKGMLSKRNTEAVKDIIKEFEEA--GYNVFIKLLNAFDYGVAQDRERVFYVGFRKD-----LNISNFE-FPYP--
tr|R7P745|R7P745_9CLOT Cytosin  FVAENVSGMLSKRHQEAVDRFIKLFEEA--GYEMHMKLLNANDYGVPQDRLRVFYVGFRKD-----LNV-DFD-FPEP--
tr|T4PMD0|T4PMD0_CLODI Modific  FLAENVKGMMAKRHNEAVTNIIQQFEEA--GYNVFIKLLNAVDYGVAQDRKRVFYIGFRKD-----LNI-NFE-FPPA--
tr|I7IJB3|I7IJB3_9BURK Cytosin  FLAENVKGMMAKRHDSAVQTIINQFGQA--GYNVFIKLLNASDYGVPQDRKRVFYIGFRKD-----LNIYDFE-FPKP--
tr|F8KQQ2|F8KQQ2_HELBC Cytosin  FLAENVSGMLAKRHKNAVDGIKNAFQDA--GYDLHFFMLNARDYGLAQDRKRVFYVGFLKD-----LRA-DFQ-PPRP--
tr|G4QBU1|G4QBU1_TAYAM Cytosin  FLAENVKGMMAKRHDSAVQAIITQFEQA--GYNVFINLLNASDYGVPQDRKRVFYIGFRKD-----LNIYDFK-FPKP--
tr|B0S4D0|B0S4D0_FINM2 Cytosin  FLAENVKGMMAKRHNKAVQSIISQFNKA--GYDVFIHLLNASDYGVAQDRKRIFYVGFRKD-----LNI-CFD-NP-P--
tr|R5X970|R5X970_9FUSO Cytosin  FLAENVKGMLSKRNTDAVKDIIKEFEKA--GYNVFIKLLNAFDYGVAQDRERVFYVGFRKD-----LNIDEFE-FPNP--
UPI000372CBB5 status=active     FLAENVSGMMAKRHNKAVKEIIGKFEKI--GYNVQIFQVNASDYGVAQDRKRVFYIGFKKE-----LEV-NFE-FPKP--
tr|U2TAM1|U2TAM1_9FUSO Unchara  FVAENVSGMLSKRHSVAVKNILNLFDES--GYDVYMDVLNAKDYGVAQDRKRVFFIGFRKD-----MKINNFQ-FPNP--
UPI00016C0771 status=active     FLAENVKGMMAQRHNEAVKGIVSQFEEV--GYDVFIKLLNANDYGVAQDRKRVFYIGFRKD-----LNI-KFE-FPKP--
tr|A8MHW3|A8MHW3_ALKOO Cytosin  FVAENVSGMMAKRHSEAVQNIISHFAGAGRGYNVFIQTLNANDFQVPQDRKRVFYIGFRKD-----LGIADFK-FPKP--
tr|G8X6Y8|G8X6Y8_FLACA Cytosin  FLAENVSGMLLGRHSEALKNIQELFRNAGIGYELSFEMLNASDYDVPQDRKRVFFIGIRKD-----LKF-KFI-FPKS--
tr|I4AHT3|I4AHT3_FLELS Cytosin  FLAENVSGMLISKHNQALEGIKELFRNAGLGYELSFKMLNASDYNVPQDRKRVFFIGIRKD-----LNF-KYE-FPNE--
tr|K2Q409|K2Q409_9FLAO Cytosin  FLAENVSGMLLERNHKALENIKALFNEC--GYNLSFTMLNASDYGVPQDRKRVFFVGYRKD-----LGI-KFK-FPEPTT
UPI0003695DD5 status=active     FVAENVSGMLAKRHNQAVQNIMACFENA--GYDVFIKMLDAYDYGVAQNRKRVFYVGFRKD-----LMVDDFK-FPLP--
UPI0002E32502 status=active     FLAENVSGILHKKHQVALKNILAAFEEA--GYVVSLKLLNAKDYQVPQDRKRVILVGYHIN-----LNK-KFI-FPEP--
tr|A6EU94|A6EU94_9BACT Cytosin  FLAENVSGMLISKHTEALEGIKELFRNAGIGYELSFQMLNASDYNVPQDRKRVFFIGIRKD-----LNF-KYQ-FPTE--
tr|E8KGI3|E8KGI3_9PAST Cytosin  FLAENVSGMLANRHSEAVQNLLKMFDEC--GYDVTLTMVNAKDYGVAQERKRVFYIGYRKD-----LGI-DFE-FPVGST
tr|N9DBX7|N9DBX7_9GAMM Cytosin  FLAENVSGMLLPAHKEALEHIKQMFQEI--GYKLSFELLNVADFGVPQDRKRVFFVGYRED-----LGK-RFE-FPKPIT
tr|K0C3B4|K0C3B4_CYCSP Cytosin  FLAENVSGMLAPRHRDAIKYIQELFKES--GYTLSFKLLNASDYWVAQDRKRVFFIGYRDD-----INM-EFQ-FPNP--
tr|F9GXX4|F9GXX4_HAEHA Cytosin  FLAENVSGMLANRHNDAVQNLLKMFDEC--GYDVTLTMVNAKDYGIAQERKRVFYIGFRKD-----LGI-NFE-FPIGST
tr|D1K024|D1K024_9BACE Modific  FVAENVSGMLAKRHSDAVSGFMKLFDEA--GYDVNLKMLNANDYDVPEDRDRVFYIGFRKD-----LDIHDFE-YPTP--
UPI0002E6F434 status=active     FLAENVSGMLLPAHKEALANIKQMFQEI--GYDLSFQLLNVSDFGVPQDRKRVFFIGYRKD-----LGL-KFS-FPEPMT
tr|D5ERM7|D5ERM7_PRER2 Cytosin  FLAENVSGMLADRHSGAVQNILNMFKES--GYNVSLTLVNAKDYGVAQERKRVFYIGFRND-----LNI-DFK-FPEGST
UPI0003ABCEDB status=active     FLAENVSGMLANRHNEAVQNLLKMFDES--GYDVTLTMVNAKDYGVAQERKRVFYIGFRKD-----LDI-NFQ-FPTGST
tr|L2F549|L2F549_9GAMM Cytosin  FVAENVSGMLAKRHSFAVQNILAQFEQA--GYQVFVKMLDAYDFGVAQNRKRVFYIGFRKD-----LGVNDFL-FPTP--
tr|R6WUU4|R6WUU4_9CLOT Cytosin  FLAENVSGMLLGRHSEAVKNIIELFKEC--GYNVTVTLVNAKDYGVPQERKRVFYIGFRND-----LNI-DFE-FPQGST
tr|C9PPY5|C9PPY5_9PAST Cytosin  FLAENVSGMLANRHSEAVQNILKMFEDC--GYDVTLTMVNAKDYGVAQERKRVFYIGFRKD-----LSI-SFQ-FPIGST
tr|K5TXT5|K5TXT5_9VIBR Cytosin  FLAENVSGMQHTRHANALNNIKQMFKDA--GYDLYYQMMNAVDYGAAQDRKRVIFIGFRSD-----LNI-KYT-FPEPME
tr|R5VXV7|R5VXV7_9FIRM Cytosin  FLAENVSGMLANRHSDAVQNILRLFDET--GYDVSLTLVNAKDYGVAQDRKRVFYIGFRKD-----LKI-KFE-FPKGST
tr|C2MAC7|C2MAC7_9PORP Cytosin  FVAENVSGMLSKRHQEAVQNFINLFDQA--GYNVFVELLNAYDYEVPEDRKRVFYIGFRKD-----LDIKEFE-FPVP--
tr|R7G2J8|R7G2J8_9FIRM Cytosin  FLAENVSGMLANRHSEAVKNILAMFEDC--GYDVTLTLVNAKNYGVAQERKRVFYIGFRKD-----LHI-DFR-FPEGST
tr|F0QZK5|F0QZK5_BACSH DNAcyto  FVAENVSGMLAKRHSSAVSGFMKLFDEA--GYDVNLKMLNANDYDVAEDRDRVFYIGFRKD-----LNIHDFK-YPTP--
sp|P08455|MTP2_NEIGO Modificat  FLAENVSGMLANRHNGAVQNLLKMFDGC--GYDVTLTMANAKDYGVAQERKRVFYIGFRKD-----LEI-KFS-FPKGST
tr|A5FP46|A5FP46_DEHSB Cytosin  FLAENVSGILFERHREAFSEIIKQFNEI--GYNVSYYLLNAHDYSVPQDRKRVIIVGYHVK-----TEL-YFT-PPKP--
tr|D4WI15|D4WI15_BACOV DNA (Cy  FVAENVSGMLAKRHTDAVRGFMELFDKA--GYDVNLKMLNANDYDVPEDRDRVFYIGFRKD-----LNIHNFE-YPTP--
tr|E2PDE9|E2PDE9_NEIPO Cytosin  FLAENVSGMLANRHNEAVQNLLKMFDEC--GYDVSLTMVNAKDYGVAQERKRVFYIGFRKD-----LKI-KFS-FPTGST
tr|D2EGA8|D2EGA8_9EURY DNAcyto  FVAENVPGMLSSTHKSEFNKIINQMERL--GYRVSYNIYDARNYGVPQERKRVIIVGYRKD-----IGF-SFL-PPEPTH
tr|D7FEK9|D7FEK9_HELP3 Cytosin  FLAENVRGMLAQRHKTSVKNILNAFKEC--GYEVNTHLVNAKDYGVAQERLRVFYIGFRRD-----LKV-NYI-FPKGST
tr|D1B3R7|D1B3R7_SULD5 DNAcyto  FLAENVSGMLASRHSDALENIKNHFIDS--GYNLSFKLLNAHDYKVPQDRQRLFFVGFRKD-----LNM-TFE-FPKA--
tr|D6ZKT7|D6ZKT7_MOBCV Cytosin  FLAENVSGMLANRHSEAVQNIITLFEEA--GYDVTLTMVNAKNYGVAEERKRVFYIGFRKD-----LNI-DFV-FPKGST
tr|K2EZH0|K2EZH0_9BACT Cytosin  FLAENVSGILHPKHKEAFSNIIKEFENA--GYEISCKLLNANDYDVPEDRLRVIIVGYNKK-----LKK-KFE-FPEP--
tr|F0S2I5|F0S2I5_DESTD DNAcyto  FLFENVAGILHRRHKKAFNLILTSLEKA--GYDIYYKLIDTYDYLVPQNRKRVFIIGFRKD-----LQI-SYS-FPKP--
```

```
tr|R7K6Q8|R7K6Q8_9FIRM Cytosin  FLAENVSGMLADRHSEAVRNIIEMFKEC--GYDLTVSLVNAKDYGVPQERRRVFYIGFRRD-----LGV-RFK-FPIGST
tr|R5R432|R5R432_9FIRM Cytosin  FLAENVSGMLANRHSEAVKNIISLFDEA--GYDVSLTLVNAKDYGVAEERKRVFYIGFRKD-----LKI-EFS-FPKGST
tr|D8G3S4|D8G3S4_9CYAN Modific  FVAENVSGMLHTKHKLAFGNILAAFEEA--GYVIAYKLLNAMYYNVPQDRKRVIFVGYHLS-----LKR-RFN-FDWV--
tr|D3I3G0|D3I3G0_9BACT Modific  FVAENVPGMLAKRHTEAVRGFMNLFNQA--GYDVNLKMLNANDFDVPEDRNRVFYIGFRKD-----LHINDYE-YPLP--
tr|E3D818|E3D818_GARV3 Cytosin  FLAENVSGMLANRHSSAVQNILHLFNDA--GYDVNLTLVNAKDYGVAQDRKRVFYIGFRKD-----LNI-KFS-FPKGST
UPI0001D09BA1 status=active     FLAENVSGMLANRHSQAVQNLLKMFDEC--GYDVSLTLVNAKNYGVAQERKRVFYIGFRKD-----LNI-KFQ-FPQGST
UPI00030E071A status=active     FLAENVSGMLHKKHQLALNNIITAFEEA--GYVISYKLLNAMRYNVPQDRKRVIFIGYHVD-----LKK-RFN-FALL--
tr|F1XAT6|F1XAT6_MORCA Cytosin  FLAENVSGMLANRHSDAVKSILNMFDDC--GYDVTVNMVNAKNYGVAQERKRVFYIGFRKD-----LAI-NFN-FPIGST
tr|I3CCM6|I3CCM6_9GAMM DNAmeth  FLAENVSGILLSRHQEAFQAILREFNSL--NYNVSYFLLNAHDYDVPQDRKRVIIVGYHKK-----IGK-SFT-PPSK--
tr|B6XDR0|B6XDR0_9ENTR Cytosin  FLAENVSGMEHARHKDALDNIKKMFKDA--GYDLYYKMLNAVDYGVAQDRKRVIFVGFRSD-----LNI-PYT-FPEPIE
tr|K9YXG2|K9YXG2_DACSA DNAmeth  FLAENVSGILHSKHQESFQQIFDSFRKA--GYNVAYKLLNAKFYNVPQDRIRVIFVGYHHR-----LGK-KFD-FGKL--
tr|D4XUU8|D4XUU8_ACIHA Cytosin  FLAENVSGMLAERHKSALDQIKKTFEDA--GYDLHFKLLNATDYGIAQDRKRVIFLGFRKD-----LNV-HYT-FPEP--
tr|N8YEA4|N8YEA4_ACIBZ Cytosin  FLAENVSGMLAPRHKDALENIKNTFLKA--GYDLHFKLLNAADFGIAQDRKRVIFIGFRTD-----LNI-QYS-FPEE--
tr|B4VSA7|B4VSA7_9CYAN C5 cyto  FLAENVSGMLHKKHENALRNIIAAFEEA--GYVISYKLLNARHYNVPQDRKRVIFIGYHID-----LNK-CFK-FNTL--
sp|P29567|MTHT_METTF Modificat  FLAENVPGIVSRTHLPEFKRLVNSFIDI--GYNVEYKVLNAKDYGVPQDRKRVFIVGYRED-----LNL-KFE-FPKP--
tr|L8N028|L8N028_9CYAN DNAcyto  FLAENVSGMMHKKHQQAFGNIIASLEEA--GYIVSFALLNAKNHNVPQERKRVIFIGYHID-----LNR-RFD-FSLV--
tr|K0JMW8|K0JMW8_BRAPL Cytosin  FLVENVVGILSKKHREVFENFLLEFKIS--NYNISCKLLNANDYGVPETRKRVIVVGYHHR-----LNK-TFI-FPQP--
tr|K9XTJ8|K9XTJ8_STAC7 DNAcyto  FLAENVSGMLHKQHGLALQQIINAFEEA--GFLVNYSLLNAVDYNVPQDRKRIIFVGFRAD-----LNL-KFD-FNWL--
UPI00036A774A status=active     FLAENVSGMLANRHNRAVQNIINLFEEA--GYDISLTMVNAKDYGVAEERKRVFYIGFRKD-----LSI-KFI-FPKGST
UPI0003826E4D status=active     FLAENVPGILSPQHKPEFMKLIKKFWNI--GYVVSFMLMDARYYGVPQERMRVIVVGYHKK-----LGK-KFE-FPRG--
UPI00037126EF status=active     FLAENVSGMLHKKHEVARNNIIAAFEEA--GYEVSFQLLNSVDFNVPQHRKRVIFVGFRQD-----LEK-VFD-FETI--
tr|K9TTW7|K9TTW7_9CYAN DNAcyto  FLAENVSGILADKHAQAFTNILYQFKDA--GYELAYKLLNAQNFEVPQDRKRVIIIGYRED-----IGG-GFD-FPKG--
tr|K9RM80|K9RM80_9CYAN DNAmeth  FLAENVGGILAKKHDRAFTNILSHFKDA--GYEVTYQLLNAADFLVPQDRKRVIIIGYRED-----MGG-TFE-FPRA--
tr|R7CTJ4|R7CTJ4_9BACE Cytosin  FLIENVQGIISDKHFSTFLSFLSTLEKA--GYVVNYSLLNAADYHIPQDRYRVFIIGFLKE-----LNC-TFD-FPRP--
tr|D4WI14|D4WI14_BACOV Cytosin  FIIENVQGMISDKHFKTFLSFLSSLECA--GYVVSYSLLNAADYCIPQDRFRVFIVGFIKE-----LNG-AFK-FPEPS-
tr|F0QZK6|F0QZK6_BACSH Cytosin  FVIENVKGIISDKHLQTFLSFLSILEKV--GYIVSYALLNAADFRIPQDRYRVFVVGFLKD-----LNC-NFH-FPHP--
tr|D1K023|D1K023_9BACE Cytosin  FLIENVQGIINDKHFSTFLSFLSTLEGA--GYVVNYSLLNAADYYIPQDRYRVFVVGFLKE-----LNC-TFN-FPKP--
UPI00037FAC0D status=active     FLIENVQGIINDKHFSTFLLFLSTLENA--GYVVSYSLLNAADYHIPQDRHRVFIVGFLKK-----LNC-TFD-FPKP--
tr|R9HQV7|R9HQV7_BACVU Cytosin  FLIENVQGIINDKHFSTFLSFLSTLEDA--GYVVSYSLLNAADYHIPQDRHRVFIVGFLKE-----LNC-TFY-FPKP--
UPI0003717B77 status=active     FLAENVPGIISKRHIKEFNKIISMLEET--GYNVTYKKLDSSDYGVPQSRKRVFIIGYRKD-----LNL-KFD-FNNV--
tr|K9VHM5|K9VHM5_9CYAN DNAcyto  FLAENVSGILAPRNKEAFTYILSQFEEI--GYRVLYKLLNAKNFDVPQDRQRVIVVGYRQN-----LGF-GFE-YPQP--
tr|T4JUF1|T4JUF1_CLODI Modific  FIAENVKGILSKKHIDAFKEIILLFDRA--GYKVTYEVLNAKNYGVPQDRERVFIVGILKE-----LDI-KYN-FPEKIN
tr|S8B9I9|S8B9I9_CLOBO Cytosin  FVAENVKGIVSKAHINSFNEIVDMFKEC--GYTVTYKVVNAKDYGVPQDRQRVFIVGIRND-----IDN-NYV-FPEP--
tr|J0QZQ6|J0QZQ6_9RHIZ DNA (Cy  FVAENVHGFIHSRNTESFKRIIKLFENE--GYAVSWKLLKASDYGVPQDRNRVFIVGYHHA-----LNK-TFK-FPEI--
tr|R5PBE9|R5PBE9_9BACT Cytosin  FVIENVQGTLDDKHKQSLAFIIESLQSI--GYSIKYKLINASDFKVPQDRYRVFFIGIRND-----IHN-TFT-FPLA--
tr|G6A2H7|G6A2H7_STRIT Cytosin  FLAENVKGMMAKRHNSAVENIVSQFEEA--GYDVFIHLLNASDYGVPQDRKRVFYVGFRKD-----LNI-KFE-PPKP--
tr|Q9K765|Q9K765_BACHD Cytosin  FVGENVKGMLTLGNGEIFKAIISDMDKK--GYTLFYKLLNAKDYSVPQNRERVIIVGFRND-----LGITEFT-FPEKH-
tr|H1B1E9|H1B1E9_9FIRM Cytosin  FIGENVKGLLTMGGGEIIDAIVEAFSDC--GYDVHYKLLNAKNYGVPEDRERVIIVGFRKD-----LGITEFD-YPES--
tr|A3DHU3|A3DHU3_CLOTH Cytosin  FIAENVKGILTLGDGEIIEAIIEDFASK--GYDVYPNLVNAADYGVPQDRWRVILYGFRKD-----LEVKDFK-FPEP--
tr|K4ZRS7|K4ZRS7_PAEAL Cytosin  FVAENVKGILTLGEGLILEAIIADFSEK--GYNVVPYTVNASDYGVPQDRDRVIFIGIRKD-----LNH-SFV-LPSP--
tr|F4PEX7|F4PEX7_BATDJ Cytosin  FIAENVKGMLTLGKGEVINQIIQDFSSA--GYNVQFKLVNARDYGVPQQRERVFIIGIRND-----IEF-EYD-FPAPTH
tr|E5YPJ2|E5YPJ2_9BACL Cytosin  FIAENVKGMMNIGGGEVLKQIVEDFASA--GYKMKYKLLNSRDYGVPQLRERVILVGVRND-----IDF-DYE-YPEPSH
sp|P13906|MTB1_LYSSH Modificat  FVAENVKGMMTLGGGEVFRQIVEDFGAA--GYRVEARLLNARDYGVPQIRERVIIVGVRND-----IDF-NYE-YPEITH
tr|J7X9T9|J7X9T9_BACCE Cytosin  FVAENVKGMLTLGKGEVFRQIVQDFEAA--GYRVYTKLVNSRDYGVPQLRERVFLVGVRND-----IDF-VYE-FPEPTH
tr|C7G547|C7G547_9FIRM Cytosin  FVAENVKGMMTLGKGEVFKQIVQDFAAA--GYTIYHKLLNSAEYGVPQIRERVILVGVRND-----IDF-EYV-HPEPTH
sp|P06530|MTBR_BACIU Modificat  FVAENVKGMMTLGKGEVLNQIIEDFASA--GYRVQFKLLNARDYGVPQLRERVIIEGVRKD-----ISF-NYK-YPSPTH
tr|F0TJ68|F0TJ68_LACA3 Cytosin  FIAENVKGLMTMAGGKVLSQMKEDFSAA--GYKVTANLVNAKDYGVPQSRERVFLVGVRND-----IAD-KFQ-YTVPEP
tr|B8DET5|B8DET5_LISMH Modific  FVAENVKGLMTLAKGEVFNQVVQDFSSA--GYKVKAFLVNSRDYGVPQLRERVFLIGTHET---KQPNF-EYI-LPPPTN
tr|B1HWG5|B1HWG5_LYSSC Modific  FVAENVKGMMTLGKGEVLRQIVEDFSSA--GYEVTPYLVNSRDFGVPQLRERVFLIGVHKEKIAEKYGF-KYP-KPQPTH
tr|Q8GC38|Q8GC38_LEUCI Putativ  FVAENVKGLLTLAKGEVFKQVVEDFASA--GYDVQAKLVNARDYGVPQSRERVFLVGTNIKKVKQKFDW-KYK-FPNPTH
tr|K4Z5B6|K4Z5B6_ENTFL DNAcyto  FIAENVKGMMTLGKGEVLNQIIEDFASA--GYTVTPHLVNARDYGVPQLRERVFLVGVHKEKIEKKYGY-HYI-KPEPTH
tr|S3P180|S3P180_9GAMM Cytosin  FIVENVSGMTSATFQHLLQDQIKVFSEA--GYNVQFKVLNASDYGVAQERKRIFIVGIRND-----YGL-EFQ-FPEPTH
tr|N9NU94|N9NU94_9GAMM Cytosin  FIVENVSGMTRATYDHLLKDQIKVFSEA--GYNVTYKVLNAADYGVAQERKRIFIVGIRAD-----LGL-NFS-FPEATH
tr|E7GW62|E7GW62_STRAP Modific  FVAENVKGMMTLGKGEVLKQIIEDFSSA--GYTVTAHLVNARDYGVPQSRERVFLVGVHKEKIEKKYGY-RYE-LPTPTH
tr|I0QBF4|I0QBF4_STROR Modific  FVAENVKGMMTLGKGEVLKQIIEDFSSA--GYTVSAHLVNARDYGVPQSRERVFLVGVHKKKIEERFGY-RYE-LPFPTH
tr|S4B3K9|S4B3K9_ENTCA Modific  FVAENVKGLMTMANGQVLNQIEQDFSAA--GYKVSTQLVNARDYGVPQSRERVFIVGVRKD----IVDTYNFE-YACPSA
tr|S9C1L1|S9C1L1_STRAG Restric  FVAENVKGMMTLGKGEVLRQIIEDFSSA--GYTVTAHLVNARDYGVPQARERVFLVGVHKEKIEERFGY-HYE-LPQPTH

                                      330       340       350       360       370       380       390       400
                                ....|....|....|....|....|....|....|....|....|....|....|....|....|....|....|....|
MHaeIII stabilized reference    ------HL------IKPTLKDVIWDLKD-NPIPALDKNKTNGNKCIYPN-HEYFIGSYSTIFMSRNRVRQWNEPAFTVQA
tr|A8SMR4|A8SMR4_9FIRM Cytosin  ------YE------TKLTFKDTIFDLKD-SAIPALEKNKTNGDNCKVLN-HEYFIGAYSPIFMSRNRVRQWDEQAFTVQA
tr|G9QUQ3|G9QUQ3_9PROT Cytosin  ------YS------YKMTFKDAIYDLKD-SAIPALSQNKTNGDKCQVLN-HEYFIGAYSPIFMSRNRVRQWNEQAFTVQA
tr|C7HVB2|C7HVB2_9FIRM Cytosin  -K----PY---D--YKLTFRDAIYDLKD-NAIPALEKNKTNGDNCNFSN-HEYFIGAYSPIFMSRNRVRDWDEQAFTVQA
tr|D1AWH4|D1AWH4_STRM9 Cytosin  -K----QY---G--YKVTFKDAIYDLKD-TAIPALEKNKTNGDKCKVLN-HEYFIGAYSPIFMSRNRVRQWEEQAFTVQA
sp|P34906|MTF1_FUSNU Modificat  -I----SE---K--ERKYLKDSIWDLKD-NALPGKDKNKTNADDCIVEN-HEYLTGSYSTIFMSRNRVRQWEQPAFTVQA
tr|R7P745|R7P745_9CLOT Cytosin  ------LK------EKPTLRDAIYDIKD-SAIPAVLKSKTNGDNCIIPN-HEYMTGSFSSIYMSRNRVRSWDEPSFTIQA
tr|T4PMD0|T4PMD0_CLODI Modific  ------FN------EKKCFKDIIFDLKD-NALPALEKNKTNGMSCKVLN-HEYFTGAYSTIFMSRNRVRSWEEPAFTVQA
tr|I7IJB3|I7IJB3_9BURK Cytosin  ------VS------KRRTLRDSILDLNN-SAIPALEKNKTNGTKCLVPN-HEYFIGEYSPIFMSRNRVRSWDEPAFTVQA
tr|F8KQQ2|F8KQQ2_HELBC Cytosin  ------LE------HSPTLRDVIWDLKD-SAVPALEKNKTNGDVLKIPN-HEYFVGSYSTIFMSRNRVRSWDEPAFTIQA
tr|G4QBU1|G4QBU1_TAYAM Cytosin  ------LS------KKCTLKDSIFDLAE-LAIPALEKNRTNGSNCLVPN-HEYFIGEFSPIFMSRNRVRSWDEPAFTVQA
tr|B0S4D0|B0S4D0_FINM2 Cytosin  -K----KF---N--YKPTLKDAIYDLKD-TAIPSLNRNKTNGDNCKVTN-HEYFTGSYSPIFMSRNRVRSWNEPAFTIQA
tr|R5X970|R5X970_9FUSO Cytosin  -I----KN---E--KRKYLKDIIWDLKD-NALSGKEKNKTNGENCIVEN-HEYLTGTYSTIFMSRNRVRQWEQPAFTVQA
UPI000372CBB5 status=active     ------YD------FKLTLKDVIYDLKE-TPIKALEKNKANKEKCIYPN-HEYYLGSYSTIFMSRNRVRQWDEQAFTVQA
tr|U2TAM1|U2TAM1_9FUSO Unchara  -V----AE---K--NRKVMRDILLDLEK-TAIPAKEKNKSNHDKCIVKN-HEYFTGNYSTIYMSRNRVRNWNEVSFTIQA
UPI00016C0771 status=active     ------QE------YKPVLRDILWDLKD-NAVPALDKNKKNASVKFLS--HEYFTGAFSTMFMSRNRVRQWDEPGFTVQA
tr|A8MHW3|A8MHW3_ALKOO Cytosin  ------IE------PRLTLKDAIWDLRE-TAIPALDKNKTNGDQCIVLN-HEYFIGSYSTIYMSRNRVRSWDEPSFTIQA
tr|G8X6Y8|G8X6Y8_FLACA Cytosin  ------YF------KKCTLENAIFDLKD-TAIPAKQGNKTNSCDCKYPN-HEYMTGDFSTIFMSRNRVRSWDEQSFTIQA
tr|I4AHT3|I4AHT3_FLELS Cytosin  ------KF------EKVTLETAISDLQD-SILPALELNNTNGNNCKVPN-HEYMIGGFSTMFMSRNRVRKWDEQSFTIQA
tr|K2Q409|K2Q409_9FLAO Cytosin  SK------------KKITLENKISDLKE-TAIPALQGNYTNGDQCSVPN-HEYLIGGFSSMYMSRNRVRSWDETSFTIQA
UPI0003695DD5 status=active     ------LD------HKFVLQDVIFDLKD-NAIPALINNKTNGDNCIIAN-HEYFVGGYSPIYMSRNRVRAWNEPSFTIQA
UPI0002E32502 status=active     ------NS------NYLTLKDTIWDLKE-SALPAQEKNKTNGSKCIVPN-HEYMTGGFSTIYLSRNRVRGWDEPSFTIQA
tr|A6EU94|A6EU94_9BACT Cytosin  ------TF------PKIPLEDVISDLQE-GVLPALEYNNTNGDNCVVPN-HEYMIGSFSTIFMSRNRVRSWDEQSYTIQA
tr|E8KGI3|E8KGI3_9PAST Cytosin  ID----DD------KKISLKDIIWDLKD-TAVPALERNQTNPQ--AVNN-NEYFTGSFSPIFMSRNRVKAWHEQGFTVQA
tr|N9DBX7|N9DBX7_9GAMM Cytosin  PI------------DKITLAQAIGDLSE-NVLPAKDGNLTNGADCKVLN-HEYMTGGFSSIYMSRNRVRSWDEVSFTIQA
tr|K0C3B4|K0C3B4_CYCSP Cytosin  ------VG------KKRYLKDIIWELAD-TALPSVGKNHTNGSKCTLPN-HEYLTGSFSSIYMSRNRVRAWDEHSFTIQA
tr|F9GXX4|F9GXX4_HAEHA Cytosin  SS----DE------KKITLKDIIWDLKD-TAVPALERNLKNPA--AINN-NEYFIGSFSPIFMSRNRVKAWHEQAFTVQA
tr|D1K024|D1K024_9BACE Modific  ------LK------HKPTLRESIWDLQD-SAIPALEKNKTNGNACKVPN-NEYFIGAYSPIFMSRNRVRSWDEPGFTVQA
UPI0002E6F434 status=active     TN------------DKITLQDVIFDLKD-NVLPAKEGNYTNGEDCEISN-HEYMIGGFSSIFMSRNRVRSWDEVSYTIQA
tr|D5ERM7|D5ERM7_PRER2 Cytosin  RD----DK------KKITLRDIIWDLKD-SAVPALEKNHHNPE--AINN-NEYFIGAYSTIYMSRNRVKAWDEQGFTVQA
UPI0003ABCEDB status=active     VD----DN------KKISLKDIIWDLQD-TAVPALERNQTNPE--AINN-NEYFIGSFSPIFMSRNRVKSWHEQGFTVQA
tr|L2F549|L2F549_9GAMM Cytosin  ------LE------NKLVLKDVIFDLKD-NAISALPNNKTNGDKCQIAN-HEYFIGGYSPIYMSRNRVRAWHEPSFTIQA
tr|R6WUU4|R6WUU4_9CLOT Cytosin  KD----NN------KKITLRDTIWDLQF-TAVPAGDKNYHNPN--AVNN-NEYFTGAFSPIFMSRNRVKSWDEQAFTVQA
tr|C9PPY5|C9PPY5_9PAST Cytosin  VN----DE------DKITLKDIIWDLQE-TAVPALAQNKSNPQ--AVNN-NEYFTGSFSPIFMSRNRVKSWNEQGFTVQA
tr|K5TXT5|K5TXT5_9VIBR Cytosin  KT------------ERRTLKHVIADLDG-TAVPALDKQKPNPDVKVLN--HEYMIGGFSTMFMSRNRVRTWDEPSFTIQA
```

```
tr|R5VXV7|R5VXV7_9FIRM Cytosin  ED----DD------KKITLRDIIWDLQD-TAVPAAEKNKHNPN--AINN-NEYFTGAFSTIFMSRNRVRDWDEQGFTVQA
tr|C2MAC7|C2MAC7_9PORP Cytosin  ------IP------KRLTLRDAIWDLRD-TAISARDSNKSNEDACLVPN-HEYFTGSYSPIYMSRNRVRSWDEPGFTVQA
tr|R7G2J8|R7G2J8_9FIRM Cytosin  VD----NE------KKLTLRDIIWNLQD-TAVPTSPHNKHNPN--AINN-NEYFTGAFSPIFMSRNRVKSWDEQAFTVQA
tr|F0QZK5|F0QZK5_BACSH DNAcyto  ------LK------HKPTLRECIWDLQD-TAIPARDKNKTNGDACIVPN-NEYFTGAYSPIFMSRNRVRSWDEPGFTVQA
sp|P08455|MTP2_NEIGO Modificat  VE----DK------DKITLKDVIWDLQD-TAVPSAPQNKTNPD--AVNN-NEYFTGSFSPIFMSRNRVKAWDEQGFTVQA
tr|A5FP46|A5FP46_DEHSB Cytosin  ------IA------HKPLLRDAIWDLRE-TAKPGGKTNKSIGN-LEFPN-HEYMVGGFSTIFMSRNRVRSWEEPSFTIQA
tr|D4WI15|D4WI15_BACOV DNA (Cy  ------QK------HKPTLRESIWDLQF-TAIPALEKNKTNGKACKIPN-NEYFIGAYSPIFLSRNRVRSWDEPGFTVQA
tr|E2PDE9|E2PDE9_NEIPO Cytosin  VK----DE------DKITLKDIIWDLQD-TAVPAAFQNKANPN--AINN-NEYFIGSFSPIFMSRNRVKAWHEQAFTVQA
tr|D2EGA8|D2EGA8_9EURY DNAcyto  SKSINSSSSNTSLQKWVTLKQAISDLPQ--AVPALDKNKPNPS-VKFPN-HEYMVGSFSTIYMSRNRIRGWDEQSFTIQA
tr|D7FEK9|D7FEK9_HELP3 Cytosin  HL------------KKLTLKDVIWDLKD-SVVCALAKNKRNPN--AINN-HEYFIGSYSPIFMSRNRVKNWDEQAFTIQA
tr|D1B3R7|D1B3R7_SULD5 DNAcyto  ------FP------LKRYLKDVIWDLKD-NALPAKEKNYTNGHACTLAN-HEYMTGSFSTIFMSRNRVRNWDEPSFTIQA
tr|D6ZKT7|D6ZKT7_MOBCV Cytosin  ED----DA------KKITLRDVIWDLQE-TAVPAAEKNRHNPQ--AINN-NEYFTGAFSPIFMSRNRVKNWDEQAFTVQA
tr|K2EZH0|K2EZH0_9BACT Cytosin  ------QK------YKPVLKDAIYDLRL--AKPALDKNKTNGDVLKIPN-HEYMNGGFSSIYMSRNRVRSWDEPSFTIQA
tr|F0S2I5|F0S2I5_DESTD DNAcyto  ------FD------RKKTLRDAIYDLKD-NVVEPLEKNRHNPK-VIVPN-HEYYIGSFSSIYMSRNRVRSWNEPSFTIQA
tr|R7K6Q8|R7K6Q8_9FIRM Cytosin  AD----DK------KKLTLRDTIWDLQY-TAVPAGARNYHNPK--AINN-NEFFTGAYSPIFMSRNRVKGWDEQAFTVQA
tr|R5R432|R5R432_9FIRM Cytosin  EE----DG------KKITLRDVIWDLQE-TAVPAEEKNHHNPK--AINN-NEYFIGEYSPIFMSRNRVKAWDEQAYTVQA
tr|D8G3S4|D8G3S4_9CYAN Modific  -G----KS---R--RILTLKDAIGDLQE-SVIPALSPNKTNGNDCLVPN-HEYMTGGFSSMYMSRNRVRCWNEPSFTIQA
tr|D3I3G0|D3I3G0_9BACT Modific  ------QK------HKPTLREAIWDLQE-TAIPAREKNHTNGDACIVPN-NEYFIGAYSPIFMSRNRVRSWDEPGFTVQA
tr|E3D818|E3D818_GARV3 Cytosin  ED----DE------KKLTLKDVIWDLQD-TAIPARERNHHNPV--AINN-NEYFIGAFSSIFMSRNRVRSWNEQGFTVQA
UPI0001D09BA1 status=active     AD----DK------HKLTLKDVMADLQK-TAVPASEKNYANPQ--AINN-NEYFTGSFSTIFMSRNRVKAWNEQGFTVQA
UPI00030E071A status=active     -K----QD---S--HTPTLKETIWDLRE-SALPALEKSKTNGEFCLVQN-HEYMIGGFSSMYMSRNRVRSWNEISFTIQA
tr|F1XAT6|F1XAT6_MORCA Cytosin  AE----DD------NKITLKDVIWDLQQ-TAVAALDKNQTNPK--AINN-NEYFIGSFSPIYMSRNRVKAWHEQGFTVQA
tr|I3CCM6|I3CCM6_9GAMM DNAmeth  ------VL------PKPTLKDAIWDLRL-TMPAKEKKGAYEGENFPILN-HEYVNGGFSSIYMSRNRVRSWDEPSFTIQA
tr|B6XDR0|B6XDR0_9ENTR Cytosin  KD------------KRKVLKDIIKDLDG-LAVPALSGQKPNPNVQVVN--HEYMIGDFSSIFMSRNRVRAWDEPSFTIQA
tr|K9YXG2|K9YXG2_DACSA DNAmeth  -T----PQ---N--NIPTLKDAIWNLRL-SAIPAKEKNKTRGNECLIPN-HEYMIGGFSSIYMSRNRVRNWTEPSFTIQA
tr|D4XUU8|D4XUU8_ACIHA Cytosin  ------IV------KKLVLKDIIKDLED-KAVPAINKSKPNPL-AIVPN-HEYMTGGFSSMYMSRNRVRSWDEPSFTIQA
tr|N8YEA4|N8YEA4_ACIBZ Cytosin  ------TK------ETRCLEDIIQDLEH-NVVPALNTSKPNPD-IAVAN-HEYMTGGFSSMYMSRNRVRAWNEPSFTIQA
tr|B4VSA7|B4VSA7_9CYAN C5 cyto  -V----PG---Y--RMFTLRDAIWNLRE-SAIPAFSNNKTRGEDCLVAN-HEYMVGGFSSIYMSRNRVRSWNEPSFTIQA
sp|P29567|MTHT_METTF Modificat  ------LN------KKVTLRDAIGDLPE--PKPALEKNRSNGENLEVPN-HEYMTGTFSSRYMSRNRVRSWDEVSFTIQA
tr|L8N028|L8N028_9CYAN DNAcyto  -Q----KS---L--YIPTLKNAIWDLRE-SALPALPTCKTNGENCLVRN-HEYMTGGFSSIYMSRNRVHNWDEPSFTIQA
tr|K0JMW8|K0JMW8_BRAPL Cytosin  ------EK------YKPTLKDAIYDLPL--AISAKNNNKTNGE-LEIPN-HEYINGSFSTIYMSRNRVRSWDEPSFTIQA
tr|K9XTJ8|K9XTJ8_STAC7 DNAcyto  -VKNYQIH------QLNNLKKAIWDLRE-SALPAQAKNKTNRDTCILPN-HEYAIGDFSSIYMSRNRVRSWDEPSFTIQA
UPI00036A774A status=active     EN----DD------KKITLKDIIWDLQN-TVVPAEEKNYHNPN--AINN-NEYFTGSFSPIFMSRNRVKSWNEQAFTVQA
UPI0003826E4D status=active     ------YG------KWVTLKEAIGDLLE--PRPASEKNKTNGK-LEIPN-HEYMNGGFSTIYMSRNRVRGWDEQSFTIQA
UPI00037126EF status=active     -NKQ---Q------SILTLKDAIWDLRE-SALAAKEKNQTNAQNCLIAN-HEYAIGSFSSMYMSRNRVRSWHEPSFTIQA
tr|K9TTW7|K9TTW7_9CYAN DNAcyto  ------SH------KNFTLRDAIYDLNL--IEPTKISSYLDKKNQKVPN-HEYLDSSFSSIYMSRNRVRSWDEPSFTIQA
tr|K9RM80|K9RM80_9CYAN DNAmeth  ------IN------NQLTLQNAIYDIKD--IEAIAIDSKMNQSNIVIPN-HEYKQGSFSSIYMSRNRVRSWNQPSFTIQA
tr|R7CTJ4|R7CTJ4_9BACE Cytosin  ------YG---K--PYVTLSKAIGDIME-VPHPYVN-EHVNPQYNRWLN-HDIFAGSWDAKFMARNRVRSWDEVSFTIQA
tr|D4WI14|D4WI14_BACOV Cytosin  ------QK------PLVTLQKAIGDITE-EP-HLYDNERVNQEYEKWTN-HDVFTGPFDTKFMARNRVRSWDEVSFTIQA
tr|F0QZK6|F0QZK6_BACSH Cytosin  ------LQ---N--AHITLQKAIGDINE-VPRFYADGDTVNQTYGRWLN-HDVFTGPFDAKFMSRNRVRAWNEVSFTIQA
tr|D1K023|D1K023_9BACE Cytosin  ------FG-----KPYVTLRKAIGDIME-NPHPYTNEG-VDQEYRKWLN-HDIFAGPWDAKFMARNRVRSWDETSFTIQA
UPI00037FAC0D status=active     ------LG-----KPYITLKKAIGDIVE-SPRSYTNENVIQ-EYGRWLN-HDIFMGPWDAKFMARNRVRSWDETSFTIQA
tr|R9HQV7|R9HQV7_BACVU Cytosin  ------FG-----KPYVTLRKAIGNITE-NPRSYTNENVIQ-EYGKWIN-HDIFAGLWDAKFMARNRVRSWNETSFTIQA
UPI0003717B77 status=active     -K----KR------EIVTLKEAIGDLPK--PLPAKEKNYTNGDDLEING-HEYFIGNFSSRFMSRNRRRGWDEVAYTIEA
tr|K9VHM5|K9VHM5_9CYAN DNAcyto  ------GT------NVLTLKDAIFDLQA--IAPMSVKGKLDNYHDCVPN-HECADLGFSSLYMSRNRVRSWSEPSFTIQA
tr|T4JUF1|T4JUF1_CLODI Modific  IEG-----------SYITLREAIGDLVD-NP-------------------GEYHDGSFSYIFMSRNRKRNWDEVAFTVQA
tr|S8B9I9|S8B9I9_CLOBO Cytosin  ------TY---TPKSYVTLEEAIGDLRD-NP-------------------GEWMEGSFSPIFMSRNRRRSWDEVGFTVQA
tr|J0QZQ6|J0QZQ6_9RHIZ DNA (Cy  ------LR------NKPTLRDAIGDLAN----------LKIGATKKVKN-HELLDSGYSPIFMSRNRVRSWEEQSYTILA
tr|R5PBE9|R5PBE9_9BACT Cytosin  ------AT---G--RKITLREAIGDIIE-RPRFFND-EMILTEHPARKN-HDVYTGNYDAKYMSRNRVRSWYEQSFTIQA
tr|G6A2H7|G6A2H7_STRIT Cytosin  ------YE------SKLTFKDAIFDLKD-SAIPALEKNKTNGDNCKVLN-HEYFIGAYSPIFMSRNRVRQWDEQAFTVQA
tr|Q9K765|Q9K765_BACHD Cytosin  -------------DKIVTLREALADLPEPSP-------------------EDICDAPYSSRFMSRNRKRNWDEVSYTIPA
tr|H1B1E9|H1B1E9_9FIRM Cytosin  ------SN------KIVTMREALSKVPA-------------------PNLGDVCTAPYSSRYMSRNRKRGWDNVSYTIPA
tr|A3DHU3|A3DHU3_CLOTH Cytosin  ------FP------YKVTLREAIGDMPE-------------------PKQSDICHASYSSRYMSRNRKRGWDEVSYTIPA
tr|K4ZRS7|K4ZRS7_PAEAL Cytosin  ------FN------KKVTLREALEHLPA-------------------PKDEDICDAPFSSRFMSRNRRRDWEDVSYTIPA
tr|F4PEX7|F4PEX7_BATDJ Cytosin  GEGKGKL-------PYITLKDAIIDLET-NP-------------------GEWFEGGYSPMYLSRNRKKKWTEQSFTIQA
tr|E5YPJ2|E5YPJ2_9BACL Cytosin  GEGLG-LN------PYVTLRDAIGDLVE-DP-------------------GPYFTGSFSTIYLSRNRKKTWEEQSFTIQA
sp|P13906|MTB1_LYSSH Modificat  GNEEG-LK------PYVTLEEAIGDLSL-DP-------------------GPYFTGSYSTIFMSRNRKKKWTDQSFTIQA
tr|J7X9T9|J7X9T9_BACCE Cytosin  GEGE--GL-----LPYVTLKDAIGDLEN-EP-------------------GPYFTGSYSTIFMSRNRKKNWDQQSFTIQA
tr|C7G547|C7G547_9FIRM Cytosin  GYGVEGLK------EVVTLRDAIGDLED-NP-------------------GDYFTGSYSTIFMSRNRKKLWTQPSFTIQA
sp|P06530|MTBR_BACIU Modificat  GE----ET---GLKPFKTLRDSIGDLVT-DP-------------------GPYFTGSYSSIYMSRNRKKSWDEQSFTIQA
tr|F0TJ68|F0TJ68_LACA3 Cytosin  TNGPDTDK------PYITLKEAISDLPE-HP-------------------ADYFEGTYSSIYMSRNRKKSWDDVSFTIQA
tr|B8DET5|B8DET5_LISMH Modific  GTKKGLL-------PFVTLRDSIFDLLD-KP-------------------GDFYEGSFSSMYLSRNRKKTWEEQSFTIQA
tr|B1HWG5|B1HWG5_LYSSC Modific  GDG---LK------PYVTLRDAIGDLEE-SP-------------------GPYFEGTYSPIYLSRNRKKTWEEQSFTIQA
tr|Q8GC38|Q8GC38_LEUCI Putativ  GEK---LL------PYVTLKDAIGDLPS-SP-------------------PDVFEGAFSPIFMSRNRKKSWNEQSFTIQA
tr|K4Z5B6|K4Z5B6_ENTFL DNAcyto  GDVSNINL-FDDRKPFVTLRDAIGDLEA-NP-------------------GDVFEGSYSTIYMSRNRKKSWEEQSFTIQA
tr|S3P180|S3P180_9GAMM Cytosin  GIG---LM------PKISIREALKNLPL------------------WPNVSEYYNYDFHWYYMSRNRKRGWDETSRTMVS
tr|N9NU94|N9NU94_9GAMM Cytosin  GNE---LT------AKVNLRQALKGLPD------------------WPDKKEYYDSGFHWYYMSRNRKRNWDETSRTIVS
tr|E7GW62|E7GW62_STRAP Modific  GDGTEINL-LAEKQPWVTLRDAISDLED-NP-------------------GEYFEGSYSTIYMSRNRKKSWEEQSFTIQA
tr|I0QBF4|I0QBF4_STROR Modific  GNPTEIDL-ISEKIPWVTLRDAIGDLEN-NP-------------------GEYFEGSYSTIYMSRNRKKSWEEQSFTIQA
tr|S4B3K9|S4B3K9_ENTCA Modific  THGTS-EK------PWVNLYDAIGDLPY-ET-------------------DDYYRGDYSSMYMSRNRKKTWNDQSFTIQA
tr|S9C1L1|S9C1L1_STRAG Restric  GNSEEIDL-FSEKLPWVTLKEAIGDLEN-NP-------------------GEYFEGSYSSIYMSRNRKKSWSEQSFTIQA

                                        410       420       430       440       450       460       470       480
                                ....|....|....|....|....|....|....|....|....|....|....|....|....|....|....|....|
MHaeIII stabilized reference    SGRQCQLHPQAPVMLKV------SKNLNKFV-EGKEHLYRRLTVRECARVQGFPDDFIFH--------------YESLND
tr|A8SMR4|A8SMR4_9FIRM Cytosin  SGRQCQLHPQAPVMPKV------EKNKNIFE-PGKEALYRRLTVRECARIQGFPDDFKFY--------------YTNLND
tr|G9QUQ3|G9QUQ3_9PROT Cytosin  SGRQCQLHPQAPIMPKV------EKNKNIFE-PGKENLYRRLTVRECARIQGFPDNFKFY--------------YTNLND
tr|C7HVB2|C7HVB2_9FIRM Cytosin  SGRQCQLHPQAPKMIKK------DKNKQVFE-PGKEELYRRLSVRECARIQGFPDDFKFY--------------YTNLND
tr|D1AWH4|D1AWH4_STRM9 Cytosin  SGRQCQLHPQAPKMPKV------EKNKNIFV-QGKEALYRRLTVRECARIQGFPDDFKFY--------------YTNLDN
sp|P34906|MTF1_FUSNU Modificat  SGRQCQLHPQAPTMIKI------DKNMYKFV-AGKENLYRRLSIRECARIQGFPDTFKFY--------------YTSLED
tr|R7P745|R7P745_9CLOT Cytosin  GGRHAPIHPQAPKMVEV------EKNKRIFV-PGKEHLYRRLSVRECARIQTFPDNFIFY--------------YTDVSA
tr|T4PMD0|T4PMD0_CLODI Modific  SGRQCQLHPQAPKMIKF------GKNDCRFV-EGQENLYRRLSVREAARVQGFPDDFEFV--------------YTSLND
tr|I7IJB3|I7IJB3_9BURK Cytosin  SGRQCQLHPQAPKMLKV------DVNKNIFD-PGSKHLYRRLSVRECARIQGFPDTFIFH--------------YNRVND
tr|F8KQQ2|F8KQQ2_HELBC Cytosin  SGRQCQLHPQAPKMPKI------GQNRHAFA-KESEHLYRRLSVRECARLQGFPDDFKFY--------------YQRLED
tr|G4QBU1|G4QBU1_TAYAM Cytosin  SGRQCQLHPQAPKMLKV------DVNKNIFV-PESKHLYRRLSVRECARIQGFPDNFIFH--------------YDRVDD
tr|B0S4D0|B0S4D0_FINM2 Cytosin  SGRQCQLHPQAPKMLKI------SKDKHIFV-PGKEKLYRRLTVRECARIQGFPDDFIFY--------------YKDVND
tr|R5X970|R5X970_9FUSO Cytosin  SGRQCQLHPQAPTMIKV------EKNVYKFV-DGKEELYRRLTIRECARIQGFPDTFKFY--------------YISLED
UPI000372CBB5 status=active     SGRQCQLHPQAPVMPKI------EKDKNIFA-KGYEHLYRRLTVRECARIQGFPDDFLFY--------------YDNIND
tr|U2TAM1|U2TAM1_9FUSO Unchara  SGRQAPQHPNAPKMLKV------GKDKFEFI-KGKERLYRRLSVRECARLQGFPDEFEFI--------------YDNLDT
UPI00016C0771 status=active     SGRQCQLHPQAPKMPKV------EKNKNVFA-EGQEHLYRRLSVREVARVQGFPDDFEFI--------------YTSVDT
tr|A8MHW3|A8MHW3_ALKOO Cytosin  SGRQAPQHPQAPKMEKV------ETNKQIFI-PGSEQLYRRLSVRECARVQTFPDDFKFY--------------YTGLQD
tr|G8X6Y8|G8X6Y8_FLACA Cytosin  GGRHAPIHPQAPKMKFI------EQNIRIFE-PGKEHLYRRLSVRECARIQTFPDNFIFH--------------YNSISA
tr|I4AHT3|I4AHT3_FLELS Cytosin  GGRHAPIHPQAPKMKLV------EKDVRIFV-PKYEHLYRRLSVRECARIQTFPDDFIFY--------------YKKVAA
tr|K2Q409|K2Q409_9FLAO Cytosin  GGRHAPIHPQAPKMVLT------GPDVRIFA-PGKEHLYRRLSVRECARIQTFPDDFIFH--------------YKAVAA
UPI0003695DD5 status=active     SGRQAPQHPNAPKMTKI------GENVFAFD---KHSPYRRLSVRECARIQGFDDDFEFV--------------YDNLND
```

```
UPI0002E32502 status=active     GGRHAPIHPQANKMIHV------GKDHFIFD-PNSPYSYRRLSIRECARIQTFPDAFIFD--------------YQNVAS
tr|A6EU94|A6EU94_9BACT Cytosin  GGRHAPIHPQAPKMKFI------EKNKRIFV-PGKEHLYRRLSVRECARIQTFPNDFIFH--------------YKKVAA
tr|E8KGI3|E8KGI3_9PAST Cytosin  SGRQCQLHPQAPKMEKH------GTNDYRFV-AGKENLYRRMTIREVARIQGFPDDFKFI--------------YKNTND
tr|N9DBX7|N9DBX7_9GAMM Cytosin  GGRHAPLHPQAPKMKFV------EQNIREFI-KGQEHLYRRLSIRECARIQTFPDNFFFD--------------YKKVSA
tr|K0C3B4|K0C3B4_CYCSP Cytosin  GGRHAPIHPQAPKMEFI------EQNKRIFV-PEKEHLYRRLSIRECARIQTFPDNHIFY--------------YKTLLA
tr|F9GXX4|F9GXX4_HAEHA Cytosin  SGRQCQLHPQAPKMEKH------GTNDYRFV-IGKEKLYRRMTVREVARIQGFPDDFKFI--------------YKNLND
tr|D1K024|D1K024_9BACE Modific  SGRQCQLHPQAPKMIKV------EKNLQKFV-EGSEHLYRRMTVREVARVQSFPDEFKFV--------------YEDVNY
UPI0002E6F434 status=active     GGRHAPIHPQAPKMKFI------EQNLREFV-RGSEDLYRRLSVRECARIQTFPDTFKFY--------------YKSVVA
tr|D5ERM7|D5ERM7_PRER2 Cytosin  SGRQCQLHPQAPKMIKF------DTNDCRFV-EGKEHLYRRMTVREVARVQGFPDSFKFI--------------YDKVDN
UPI0003ABCEDB status=active     SGRQCQLHPQAPKMEKY------GTNDYRFV-VGKEELYRRMTIREVARVQGFPDDFKFI--------------YKNTND
tr|L2F549|L2F549_9GAMM Cytosin  SGRQAPQHPNAPKMQQI------EPNRFEFV--GDESNYRRLSVRECARIQGFPDEFIFI--------------YDNLQD
tr|R6WUU4|R6WUU4_9CLOT Cytosin  SGRQCQIHPQAPKMTFI------SQNHREFV-KGKEHLYRRMTIREIARIQGFPDTFKFI--------------YDYTDN
tr|C9PPY5|C9PPY5_9PAST Cytosin  SGRQCQLHPQAPKMEKY------GINDYRFV-VGKENLYRRMTIREVARVQGFPDDFKFI--------------YQNTND
tr|K5TXT5|K5TXT5_9VIBR Cytosin  GGRHAPIHPGAPKMEKV------ETDKFKFV-EGHEDKYRRLSVRECARIQGFPNDFEFV--------------YNNVAA
tr|R5VXV7|R5VXV7_9FIRM Cytosin  SGRQCQLHPQAPKMVKF------GKNDCRFV-EGKEHLYRRMTIREVARVQGFPDDFKYI--------------YENTDV
tr|C2MAC7|C2MAC7_9PORP Cytosin  SGRQCQLHPQAPKMQKI------ETNKHIFD-PLNEKLYRRMTVREVARVQSFPDDFLFI--------------YDQVND
tr|R7G2J8|R7G2J8_9FIRM Cytosin  SGRQCQLHPQAPKMIKV------GKNDCRFV-EGKESLYRRMTIREVARVQGFPDDFQFI--------------YQDADD
tr|F0QZK5|F0QZK5_BACSH DNAcyto  SGRQCQLHPQAPKMIKV------EKNLQKFA-EGYEHLYRRMSVREVARIQSFPDSFKFI--------------YDDVNY
sp|P08455|MTP2_NEIGO Modificat  SGRQCQLHPQAPKMEKH------GANDYRFA-AGKETLYRRMTVREVARIQGFPDNFKFI--------------YQNVND
tr|A5FP46|A5FP46_DEHSB Cytosin  GGRHAPIHPQAPKMQLV------SKDKMEFK-PGDQHLYRRLSIRECARIQTFPDNFVFK--------------YAGLAD
tr|D4WI15|D4WI15_BACOV DNA (Cy  SGRQCQLHPQAPKMIKV------EKNLQKFA-EGYEHLYRRMTVREVARVQSFPDEFQFI--------------YDDVNY
tr|E2PDE9|E2PDE9_NEIPO Cytosin  SGRQCQLHPQAPKMEKH------GLNDYRFV-VGKEALYRRMTVREVARVQGFPDDFKFI--------------YQNIND
tr|D2EGA8|D2EGA8_9EURY DNAcyto  GGRHAPLHPDSCPMQKV------GTDKWVFT----GSYYRRLTVREAARIQTFPDNFIFY--------------YINLAQ
tr|D7FEK9|D7FEK9_HELP3 Cytosin  SGRQCQLHPQAPKMAKF------GKNDCRFI-ENYQHLYRRLSVRECARIQGFDDDFYFV--------------YENLND
tr|D1B3R7|D1B3R7_SULD5 DNAcyto  GGRHAPLHPQAPKMEFI------EQNKRIFV-KGSEHLYRRLSIRECARIQTFPDTHTFY--------------YDNLTA
tr|D6ZKT7|D6ZKT7_MOBCV Cytosin  SGRQCQLHPQAPKMVKF------GQNDCRFV-EGSENLYRRMTIREVARIQGFPDDFKFI--------------YKETNN
tr|K2EZH0|K2EZH0_9BACT Cytosin  GGRHAPIHPQAPKMKFI------EQNKRIFV-PGKEHLYRRLSVRECARIQTFPDDFIFK--------------YRDIAD
tr|F0S2I5|F0S2I5_DESTD DNAcyto  SGRHAPIHPQANKMIKV------SKDKYIFD-PESPYPYRRLSVRECARIQTFPDDFIFV--------------YEKLDA
tr|R7K6Q8|R7K6Q8_9FIRM Cytosin  SGRQCQLHPQAPKMRFI------EQNKREFV-RGKEHLYRRMTIREIARIQGFPDDFKFI--------------YNYTDD
tr|R5R432|R5R432_9FIRM Cytosin  SGRQCQLHPQAPKMVKY------GKNDCRFV-EGKEHLYRRMTVREIARVQGFPDDFKFV--------------YDGVND
tr|D8G3S4|D8G3S4_9CYAN Modific  GGRHAPIHPQANKMIRV------GKDEWIFD-VNSRQSYRRLSVRECARIQTFPDDFIFY--------------YQNLAA
tr|D3I3G0|D3I3G0_9BACT Modific  SGRQCQLHPQAPKMEKI------EKNLQRFV-PGKEHLYRRMTVREVARVQSFPDDYRFL--------------YDEVNY
tr|E3D818|E3D818_GARV3 Cytosin  SGRQCQLHPQAPKMIKF------GMNDCRFV-KGKESLYRRMTIREIARIQGFPDNFKFI--------------YDSLDT
UPI0001D09BA1 status=active     SGRQCQLHPQAPKMVKY------GENDFRFV-TGQEHLYRRMTVREVARLQGFPDNFKLI--------------YQKIDD
UPI00030E071A status=active     GGRHAPIHPQANKMIHV------GKDKWIFA-PDSLQTYRRLSVRECARIQTFPDDFIFY--------------YKDLAA
tr|F1XAT6|F1XAT6_MORCA Cytosin  SGRQCQLHPQAPKMQKH------GTNDFRFA-IGKEHLYRRMTVREVARIQGFPDDFKFI--------------YQNVND
tr|I3CCM6|I3CCM6_9GAMM DNAmeth  SGRHAPLHPQAPKMALV------ERDKRVFV-QGKEDLYRRLSVRECARIQTFPDDFIFK--------------YKNISD
tr|B6XDR0|B6XDR0_9ENTR Cytosin  GGRHAPIHPSAPKMEKV------ETDKFAFI---TGHEYRRLSVRECARIQGFPDNFEFL--------------YNNVAV
tr|K9YXG2|K9YXG2_DACSA DNAmeth  GGRHAPIHPQANKMIKV------EKDKFVFD-ENSLENYRRLSVRECARIQTFPDNFIFD--------------YDYVNN
tr|D4XUU8|D4XUU8_ACIHA Cytosin  GGRHAPIHPSSPKMIKI------DVDKFMFAYSELG--FRRLSVRECARIQSFPDSFVFK--------------YSDVNH
tr|N8YEA4|N8YEA4_ACIBZ Cytosin  GGRHAPIHPSAPKMVKL------EQDKFMFS-PSAE--YRRLSVRECARIQSFPDDFIFK--------------YSDVNH
tr|B4VSA7|B4VSA7_9CYAN C5 cyto  GGRHAPIHPQANKMIHI------GKDKWIFD-PQSPNPYQRLSVRECARIQTFDDDFIFY--------------YERLAA
sp|P29567|MTHT_METTF Modificat  GGRHAPCHPQANKMIKV------GPDKFIFD-PESPKPYRRLSVRECARIQGFPDDFIFY--------------YKNVAD
tr|L8N028|L8N028_9CYAN DNAcyto  GGRHAPIHPQANKMIWV------GKDEWMFD-PNSPNPYRRLSVRECARVQTFPDDFNFY--------------YKNLAQ
tr|K0JMW8|K0JMW8_BRAPL Cytosin  GGRHAPIHPQAPKMVFV------EKDKYIFA-KGHEDKYRRLSVRECARIQTFPDSFVFK--------------YNNIND
tr|K9XTJ8|K9XTJ8_STAC7 DNAcyto  SGRHAPIHPQANKMINI------GKDKFIFD-PNSIYPYRRLSLRECARIQTFPDNFLFD--------------YINLNA
UPI00036A774A status=active     SGRQCQLHPQAPKMQKI------GKDSFCFV-EGCEHLYRRMSVREIARIQGFPDDFQFI--------------YKKTND
UPI0003826E4D status=active     GGRHAPIHPQAPKMKLI------GKNKREFI-KGKEHLYRRLSVRECARIQTFPDNFVFY--------------YGNVAD
UPI00037126EF status=active     SSRHAPIHPQANKMINI------GEDRFIFD-PNSPYPYNRLSVRECARIQTFPDDFIFY--------------YKNINA
tr|K9TTW7|K9TTW7_9CYAN DNAcyto  GGRHAPIHPQANKMIWL------AKDKWMFD-PESPKSYRRLSVRECARIQTFPDDFIFQ--------------YNSITD
tr|K9RM80|K9RM80_9CYAN DNAmeth  GARHAPIHPQANKMIWV------EKDKWKFD-EKSPKPYRRLSVRECARIQTFPDNFIFK--------------YKYIGD
tr|R7CTJ4|R7CTJ4_9BACE Cytosin  QAKNCPLHPQAPKMKYI------SPNLRSFQ-QGAEHLYRRLSVRECARIQTFPDKFRFF--------------YDDVKN
tr|D4WI14|D4WI14_BACOV Cytosin  QAKNCPLHPQAPVMKYV------SPNQRIFL-PGYEHLYRRLSVRECARIQSFPDKFRFS--------------YTHIKE
tr|F0QZK6|F0QZK6_BACSH Cytosin  QAKNCPLHPQAPTMKYI------SPHKRIFA-VGYEHLYRRFSIRECARIQSFPDSFRFF--------------YNDIQE
tr|D1K023|D1K023_9BACE Cytosin  QAKNCPLHPQAPKMKYI------SQTQRVFQ-QGAEHLYRRLSVRECARIQTFPDKFRFF--------------YEDIKD
UPI00037FAC0D status=active     QAKNCPLHPQAPKMKYI------SQNQRVFL-QGAEHLYRRLSVRECARIQTFPDNFRFF--------------YKNVQD
tr|R9HQV7|R9HQV7_BACVU Cytosin  KAKNCPLHPQAPKMKYV------SQNQRVFL-QGAEHLYRRLSIRECARIQTFPDKFRFF--------------YDKVQE
UPI0003717B77 status=active     SGRHAKIHPSAGEMTKV------EKDKWIFD---ESKPYRRLSIRESARIQTFPDDFKFI--------------YERLND
tr|K9VHM5|K9VHM5_9CYAN DNAcyto  SGRHAPLHPQASKMILV------GKDKRIFD-PNSPLPYRRLSVRECARVQTFPDEFIFK--------------YKYIAD
tr|T4JUF1|T4JUF1_CLODI Modific  SGRQTQIHPDSPDMLPV------GRDERIFD-PESDREIRRMSVRECARLQTFPDDFEFL---------------SSVNE
tr|S8B9I9|S8B9I9_CLOBO Cytosin  SGRQTQIHPDSPEMEKI------DKDKWQFK-KDSGRTIRRMSVRECARIQTFPDCFEFL--------------SSSLNE
tr|J0QZQ6|J0QZQ6_9RHIZ DNA (Cy  TERHIPFHPQAPKMVKV-----EGQDLYEFA-KGFENKYRRLTIRECARIQTFPDDYEFV--------------YTNMRN
tr|R5PBE9|R5PBE9_9BACT Cytosin  QARNEPQHPQAPKMAYV------SSDKRIFA-KGHEHLYRRLSVRECARIQTFSDSFVFF--------------YTNIRN
tr|G6A2H7|G6A2H7_STRIT Cytosin  SGRQCQLHPQAPVMPKV-------------------------------------EKIKIY--------------LNLVRK
tr|Q9K765|Q9K765_BACHD Cytosin  MAKQVPLHPSSPDMVKL------GKDLWKFG---ENGVTRRFSWQEAAAIQTFPRDMHFV---------------GELTS
tr|H1B1E9|H1B1E9_9FIRM Cytosin  MAKQVPLWPGSPDMVKI------DKDVWRFG----DGETRRLSWQEAAAIQTFPADMQFC---------------GDLTS
tr|A3DHU3|A3DHU3_CLOTH Cytosin  MAKQVPLHPSSPDMIKI------AEDKWIFG----EGKTRRFSWQEAAVIQTFPRDMEFV---------------GNLTS
tr|K4ZRS7|K4ZRS7_PAEAL Cytosin  MAKQVPLHPSSPMMIKI------HKDLWSFG----EGKTRRFSWQEAAAIQTFPQGMEFA---------------GDLIS
tr|F4PEX7|F4PEX7_BATDJ Cytosin  SGRQAPLHPSGPEMIKL------DKDQWEL--PGDNSLHRRLSVKEIARIQTFPDWFEFSDGGNMDSSK-----NNRLNK
tr|E5YPJ2|E5YPJ2_9BACL Cytosin  SGRQAPLHPDGPLMVKM------DANWWDL--PGGEELNRRLSVKEIARIQTFPDWYDFT------TTPKKVSESSKLDY
sp|P13906|MTB1_LYSSH Modificat  SGRQAPIHPGGLPMEKV------DKNKWIF--PDGEENHRRLSVKEIKRIQTFPDWYEFSDGGNMKVSV-----NNRLDK
tr|J7X9T9|J7X9T9_BACCE Cytosin  SGRQAPIHPGGLPMENV------DKNKWVF--PDGEENNRRLSVKEIARIQTFPDWYEFSDGDNQKMSV-----NGRLDK
tr|C7G547|C7G547_9FIRM Cytosin  SGRQAPIHPAGEPMVHV------GKDKYIFS--DGEENNRRLSVKEIARIQTFPDWYEFS------RGTSNRNDNAKLDL
sp|P06530|MTBR_BACIU Modificat  SGRQAPLHPGGLSMKKI------GKDKWVF--PDGEENHRRLSVKEIARVQTFPDWFQFSQGTNSQTSI-----NNRLDK
tr|F0TJ68|F0TJ68_LACA3 Cytosin  SARQAPQYPGGKPMVKI------GRDKWEFQ----GDLNRRLSVKECARIQTFPDWFEFSDGGKD-----NVSLANRLNE
tr|B8DET5|B8DET5_LISMH Modific  SGRQAPLHPSGKPMEKL------DKDIWKLV----GNTNRRLSVKEIARIQTFPDWFEFSTGGNI-----KASKNHRLNQ
tr|B1HWG5|B1HWG5_LYSSC Modific  SGRQAPIHPSGEPMRYI------EKNKWELV-----GENRRLSVKEIARIQTFPDWFEFSDGAKE-----KMSESGRLDK
tr|Q8GC38|Q8GC38_LEUCI Putativ  SGRQAPIWPGGLPMTKI------GKDLWKFE----GEINRRLSVREISRIQTFPDWFSFS------AGDQKTNKNGKLDK
tr|K4Z5B6|K4Z5B6_ENTFL DNAcyto  SGRQAPQHPSGEPMTKL------EKNRWQFN----GENNRRLSVKEIARIQTFPDWFQFSDGENL-----NLSKNGRLDK
tr|S3P180|S3P180_9GAMM Cytosin  SARHMLLHPVSPDMVKV------GTDQWEFS---TSEPARRFTYKEAARLQGFRD-LIFP-----------REQEVNLEN
tr|N9NU94|N9NU94_9GAMM Cytosin  SARHTLLHPISPDMVKI------KTDHWEFV---SDAPARRFTYREAARIQGFGD-LIFP-----------REDEVTLEN
tr|E7GW62|E7GW62_STRAP Modific  SGRQAPQHPAGEPMTKYKDKEN--HTRWKFN----GEKNRRLSVKEISRIQTFPDWFEFSAGSPIGKSGKEISRNAQIDK
tr|I0QBF4|I0QBF4_STROR Modific  SGRQAPQHPGGSPMTQY-IDKEDGRKRWKFN-----GKNRRLSIKEIARIQTFPDWFEFSSGSPLGKSGREISRNAQIDK
tr|S4B3K9|S4B3K9_ENTCA Modific  SGRQAPQHPAGEPMEHV------GKDLWEFR----GSQNRRLSVREVARIQTFPDWYNFSDGNNG-----NATQNHRLNQ
tr|S9C1L1|S9C1L1_STRAG Restric  SGRQAPQHPAGSPMTQY-VDKTDGRKRWKFN-----GENRRLSIKEIARIQTFPDWFEFDMVSSIGKSGKEISRNAQIDK

                                        490       500       510       520       530       540       550       560
                                ....|....|....|....|....|....|....|....|....|....|....|....|....|....|....|....|
MHaeIII stabilized reference    GYKMIGNAVPVNLAYEIAKTIKSALEIRKGN-------------------------------------------------
tr|A8SMR4|A8SMR4_9FIRM Cytosin  AYKMIGNAVPVNLAYEMAKAIVSALDNSSANSNLLKTGTDN---------------------------------------
tr|G9QUQ3|G9QUQ3_9PROT Cytosin  AYKMIGNAVPVNLAYVIANEIMLALNKNKFR-------------------------------------------------
tr|C7HVB2|C7HVB2_9FIRM Cytosin  AYKMIGNAVPVNLAYEMAKAIDLALNKPSIFSYPKQLQMI----------------------------------------
tr|D1AWH4|D1AWH4_STRM9 Cytosin  AYKMIGNAVPVNLAYEIAKAINELFNN-----------------------------------------------------
sp|P34906|MTF1_FUSNU Modificat  GYKMVGNAVPVDLAYIIAKRIKETLTDKEKIKKEIRQKTLFD--------------------------------------
tr|R7P745|R7P745_9CLOT Cytosin  GYKMIGNAVPVNLAKAVGNQIRKSLEVLENEK------------------------------------------------
```

```
tr|T4PMD0|T4PMD0_CLODI Modific  GYKMIGNAVPVNLAYILAKQIKKYLENN----------------------------------------------------
tr|I7IJB3|I7IJB3_9BURK Cytosin  GYKMIGNAVPVELARIIANAISDKLNTLIRTPKLNYGT------------------------------------------
tr|F8KQQ2|F8KQQ2_HELBC Cytosin  GYKMVGNAVPVILAYKIARAIKKTLDTSIRVAI-----------------------------------------------
tr|G4QBU1|G4QBU1_TAYAM Cytosin  GYKMIGNAVPVELARIIAKSISEKLKKLVRTPKLKYGT------------------------------------------
tr|B0S4D0|B0S4D0_FINM2 Cytosin  GYKMIGNAVPINLAFVIAMNIKSHIMSVYC--------------------------------------------------
tr|R5X970|R5X970_9FUSO Cytosin  GYKMVGNAVPVDLAYVIAEKIKKTLTDKKKLKKKIKEKTLFD--------------------------------------
UPI000372CBB5 status=active     GYKMIGNAVPINLAYEIAKKIREVLEKKERSKINE---------------------------------------------
tr|U2TAM1|U2TAM1_9FUSO Unchara  GYKMIGNAVPVNLAYEIAKHIKQYLL------------------------------------------------------
UPI00016C0771 status=active     GYKMIGNAVPVGLAYAVAEQIKKYI-------------------------------------------------------
tr|A8MHW3|A8MHW3_ALKOO Cytosin  AYKMIGNAVPVELAYHVANQIMTYLK------------------------------------------------------
tr|G8X6Y8|G8X6Y8_FLACA Cytosin  GYKMIGNAVPVNLAFHLAESIKEQIIKNEILIENLYLTKQT---------------------------------------
tr|I4AHT3|I4AHT3_FLELS Cytosin  GYKMIGNAVPVNLAYFLAKSIKEQILSNQKKNFIQQEKLTA---------------------------------------
tr|K2Q409|K2Q409_9FLAO Cytosin  GYKMIGNAVPVDLAHVIASQIKKDLNQLKTKKQIKTNKADTSSTPINGNYIKEKMLELVNS-------------------
UPI0003695DD5 status=active     GYKMIGNAVPVNLAYAVAKEIYQVLNNL----------------------------------------------------
UPI0002E32502 status=active     AYKMLGNAVPVNLAYALARVIFDDLRGVESFTEKEKSTTMVGVNIN----------------------------------
tr|A6EU94|A6EU94_9BACT Cytosin  GYKMIGNAVPVNLAKFLANSIMEQIKANHKTNKAVKKTQKLKEAIAV---------------------------------
tr|E8KGI3|E8KGI3_9PAST Cytosin  AYKMIGNAVPVNLAYEIAVGIKKALEMSKSKKQIVRVVQTHQPIQPTQQLSMF---------------------------
tr|N9DBX7|N9DBX7_9GAMM Cytosin  AYKMIGNAVPVKMGKTLAEKIYSDLNAIKISTNRTFSEEVNGAVVKEKLSEIVNRMSA----------------------
tr|K0C3B4|K0C3B4_CYCSP Cytosin  GYKMVGNAVPPNLAYFLAKQIYSDLAFANKIQSQKYEVREPAIASSYLASNTYSEFDLAEVVA-----------------
tr|F9GXX4|F9GXX4_HAEHA Cytosin  AYKMIGNAVPVNLAYEIALSIKQALQDSVLPKN-----------------------------------------------
tr|D1K024|D1K024_9BACE Modific  AYKMIGNAVPVNLAYHVAMQIRKTLVEKGIVIA-----------------------------------------------
UPI0002E6F434 status=active     GYKMIGNAVPVKMGEILANRILKDLKGLKVKSDVKMQKVITKVNGGVVRERMVEIINNIPVNA-----------------
tr|D5ERM7|D5ERM7_PRER2 Cytosin  AYKMIGNAVPVNLAYEIALAIKDAIEF-----------------------------------------------------
UPI0003ABCEDB status=active     AYKMIGNAVPVNLAFEIAVAIKNALEGETMKNSIIPHQQNSQLSMF----------------------------------
tr|L2F549|L2F549_9GAMM Cytosin  GYKMIGNAVPVNLAYAIGRRVYGVLLGLGEK-------------------------------------------------
tr|R6WUU4|R6WUU4_9CLOT Cytosin  AYKMIGNAVPVNLAYEVAVAIKQALQAKEEK-------------------------------------------------
tr|C9PPY5|C9PPY5_9PAST Cytosin  AYKMIGNAVPVNLAFEIACAIKKELD------------------------------------------------------
tr|K5TXT5|K5TXT5_9VIBR Cytosin  GYKMIGNAVAVPFAKALALSIQEHLAQAKDLKAA----------------------------------------------
tr|R5VXV7|R5VXV7_9FIRM Cytosin  AYKMIGNAVPVNLAYEVALGIKAALE------------------------------------------------------
tr|C2MAC7|C2MAC7_9PORP Cytosin  GYKMVGNAVPVNLAYHVALSIKQALKQAGLEIHKSEPFVEQQSQRSMTQSTLFTQ-------------------------
tr|R7G2J8|R7G2J8_9FIRM Cytosin  AYKMIGNAVPVNLAYEIAIAIKNTLETIIFR-------------------------------------------------
tr|F0QZK5|F0QZK5_BACSH DNAcyto  GYKMIGNAVPVNLAYHVALQIKKTLIEKGAIQPG----------------------------------------------
sp|P08455|MTP2_NEIGO Modificat  AYKMIGNAVPVNLAYEIAAAIKKTLER-----------------------------------------------------
tr|A5FP46|A5FP46_DEHSB Cytosin  GYKMVGNAVPVEFAYNIAMKISEDLRSYEKRTDTKR--------------------------------------------
tr|D4WI15|D4WI15_BACOV DNA (Cy  GYKMIGNAVPVNLAYHVAMQIKKTLVEKGIIVDLK---------------------------------------------
tr|E2PDE9|E2PDE9_NEIPO Cytosin  AYKMIGNAVPVNLAYEIAVAIKKELII-----------------------------------------------------
tr|D2EGA8|D2EGA8_9EURY DNAcyto  GYKMVGNAVPVKLAEAIAKRIMSDMKTFSKATEKICAKD-----------------------------------------
tr|D7FEK9|D7FEK9_HELP3 Cytosin  AYKMIGNAVPVSLAKEIAISIYKVLH------------------------------------------------------
tr|D1B3R7|D1B3R7_SULD5 DNAcyto  GYKMVGNAVPPNLAFFLADAIAKQLTQANILKKVS---------------------------------------------
tr|D6ZKT7|D6ZKT7_MOBCV Cytosin  AYKMIGNAVPVNLAFEIATAIRLFLTGRGHEVKGMAPKGKKAHGCKQQ--------------------------------
tr|K2EZH0|K2EZH0_9BACT Cytosin  GYKMIGNAVAVNFAQHLAKKIYEDLKI-----------------------------------------------------
tr|F0S2I5|F0S2I5_DESTD DNAcyto  GYKMIGNAVPVKLAEILAVSIKETLRRKIGKERTKILLPLFLA-------------------------------------
tr|R7K6Q8|R7K6Q8_9FIRM Cytosin  AYKMIGNAVPVNLAYEVAIAIKKVLQ------------------------------------------------------
tr|R5R432|R5R432_9FIRM Cytosin  AYKMIGNAVPVNLAYEIAVGIKDALKNKND--------------------------------------------------
tr|D8G3S4|D8G3S4_9CYAN Modific  GYKMIGNAVPVNFSYAVAQAIYEDLFNEKQESKEREECSTYI--------------------------------------
tr|D3I3G0|D3I3G0_9BACT Modific  GYKMIGNAVPVNLAYHVAMSIIETLKRHNTNFTD----------------------------------------------
tr|E3D818|E3D818_GARV3 Cytosin  GYKMIGNAVPVNLAFEIATAIKKVLS------------------------------------------------------
UPI0001D09BA1 status=active     GYKMIGNAVPVQLAYEIAVAIKQVFQAA----------------------------------------------------
UPI00030E071A status=active     GYKMVGNAVPVNFSYALAQAIYTDLFSVKREIVEYKPLPLQLTLNLF---------------------------------
tr|F1XAT6|F1XAT6_MORCA Cytosin  GYKMIGNAVPVNLAYEIAIAIHDCLQNHQATNHL----------------------------------------------
tr|I3CCM6|I3CCM6_9GAMM DNAmeth  GYKMVGNAVPVKFAKHLASVIFRDILE-----------------------------------------------------
tr|B6XDR0|B6XDR0_9ENTR Cytosin  GYKMIGNAVAVPFAKILATSIRKHLDHIN---------------------------------------------------
tr|K9YXG2|K9YXG2_DACSA DNAmeth  GYKMVGNAVPVNFARAIAQIIYYDLLNQTIPVDLNQRQSEPIQLTLPVG-------------------------------
tr|D4XUU8|D4XUU8_ACIHA Cytosin  GYKMIGNAVNVDFAKILADSISQALHLSCKANLRSA--------------------------------------------
tr|N8YEA4|N8YEA4_ACIBZ Cytosin  GYKMIGNAVNVDFAKILAESILNALNQANSILKAS---------------------------------------------
tr|B4VSA7|B4VSA7_9CYAN C5 cyto  GYKMIGNAVPVNLSYALAKSIYQDLLAADSPNKPNQIQVHPITTS-----------------------------------
sp|P29567|MTHT_METTF Modificat  GYTMVGNAVPVKLAEELAKKIKKDLEGVLN--------------------------------------------------
tr|L8N028|L8N028_9CYAN DNAcyto  GYKMIGNAVPVNLGYALAKAIKLQIVEVDDTSRNIYQLQSKDSNYLKVGILNKASIQRVG--------------------
tr|K0JMW8|K0JMW8_BRAPL Cytosin  AYKMIGNAVPVNLAFKIAKKIRKDLECL----------------------------------------------------
tr|K9XTJ8|K9XTJ8_STAC7 DNAcyto  GYKIVGNAVPVNLGYALAEAIKSYLTEPINIKQNEQSLQLSLNLN-----------------------------------
UPI00036A774A status=active     AYKMIGNAVPVNLAYEIAIAIKMFLQGEGDKVNGIEMERNK---------------------------------------
UPI0003826E4D status=active     GYKMIGNAVPVKLAEAIAKKIMQDLKGMQEKDRGVICQTESQKSKEAKLCLASNQ-------------------------
UPI00037126EF status=active     GYKMIGNAVPVNFSQALATAIKEQLLSHETAYRQRNIKAVQLNLDLDLTSAN----------------------------
tr|K9TTW7|K9TTW7_9CYAN DNAcyto  GYKMVGNAVPVNLARILATKIKEDIQQYLTFGVCKTVRQHQLCMQLTLLNTKSTHLF-----------------------
tr|K9RM80|K9RM80_9CYAN DNAmeth  GYKMIGNAVPVMLAKILSEKVFLDIKQYLESGFYKAVIKQEVPRQLTLF-------------------------------
tr|R7CTJ4|R7CTJ4_9BACE Cytosin  GYKMVGNAVPPRLAKVLALSIKDALISAQASQK-----------------------------------------------
tr|D4WI14|D4WI14_BACOV Cytosin  GYKMVGNAVPPRLARCLALSIKDALGSMNGKKEADV--LVAYYKDEHQLRMTLRNKLYYVRTGFRRGALQMPIGATSPKY
tr|F0QZK6|F0QZK6_BACSH Cytosin  GYKMVGNAVPPRLAKFIALNIKNAFTSIHSAEKEF--VLVGYYKDEKQLHLTLQNKLYYVRSGFRRGALQMPVGMPVPAY
tr|D1K023|D1K023_9BACE Cytosin  GYKMVGNAVPPRLAKFLALSIKKALVSVEERKAETINVLVAYYKDNNQLRQTLKNKLYYVRAGLRRGALQIPIGMSYPIY
UPI00037FAC0D status=active     GYKMVGNAVPPRLAKFLALSIKEALFSIQVRKSETSNVLVAYYKNDYQLQQTLRNRLYYVRAGLRRGALQIPVGMAYPIY
tr|R9HQV7|R9HQV7_BACVU Cytosin  GYKMVGNAVPPRLAKFLALAIKDSLNASQVKDTKPVNVLVAYYKDDNQLRLTLENRLYYVRAGLRRGALQIPKGIAYPVY
UPI0003717B77 status=active     GYKMIGNAVPVKLSKVLAEVISNDLDSIKNLTNKKVSSF-----------------------------------------
tr|K9VHM5|K9VHM5_9CYAN DNAcyto  GYKMVGNAVPVNLARVLASKIFGDIVLGHRASAKQGIVMATCQSARE---------------------------------
tr|T4JUF1|T4JUF1_CLODI Modific  NYKMIGNAVPVKLAEEVAKQLKVALESKGIK-------------------------------------------------
tr|S8B9I9|S8B9I9_CLOBO Cytosin  NYKMIGNAVPVKLAEAIAKNLKKCIEEGTY--------------------------------------------------
tr|J0QZQ6|J0QZQ6_9RHIZ DNA (Cy  AYKMIGNAVPIKLAYWIAKTIKKDLSRNDNIETANTD-------------------------------------------
tr|R5PBE9|R5PBE9_9BACT Cytosin  GYKMVGNAVPPRLAYALAVQIQKMCFR-----------------------------------------------------
tr|G6A2H7|G6A2H7_STRIT Cytosin  HY------------------IED---------------------------------------------------------
tr|Q9K765|Q9K765_BACHD Cytosin  KYKQIGNAVPVKLAEAVAEKVYEKLNECLRNNQQEYKEKVVV--------------------------------------
tr|H1B1E9|H1B1E9_9FIRM Cytosin  VYKQIGNAVPCKLAEAIAIRVRDVLENIDESEEN----------------------------------------------
tr|A3DHU3|A3DHU3_CLOTH Cytosin  KYRQIGNAVPVKLAEVIGKKLYEILEEKLNANVKELEQKVGE--------------------------------------
tr|K4ZRS7|K4ZRS7_PAEAL Cytosin  KYKQIGNAVPVKLAEVIAVALRQCLTNELEKRHNTY--------------------------------------------
tr|F4PEX7|F4PEX7_BATDJ Cytosin  QYKQIGNAVPVDLAKAIAMPIALWAKGNMESIKQLVLF------------------------------------------
tr|E5YPJ2|E5YPJ2_9BACL Cytosin  IYKQIGNAVPVLLAKAIAKPLADWAVKFKENEELLVESIGRLETNRLRRRGKQWSLLLRRATSPRNRGKRVSKVFNRSSY
sp|P13906|MTB1_LYSSH Modificat  QYKQIGNAVPVFLARAVAKSIAQFAADYLKDNHPHEAPQMKLFI------------------------------------
tr|J7X9T9|J7X9T9_BACCE Cytosin  IYKQIGNAVPVLLARAIVKPIAEYAVNQPVHQDDQLVFNI----------------------------------------
tr|C7G547|C7G547_9FIRM Cytosin  VYKQIGNAVPVRLALAVAEPIAVFAKELLEKEKEEAEYVVIRNVGEQKRMMA----------------------------
sp|P06530|MTBR_BACIU Modificat  QYKQIGNAVPVLLAKAVASPIANWAINYLESSPNNKIKNRERKLSIRTFLRIKTS-------------------------
tr|F0TJ68|F0TJ68_LACA3 Cytosin  KYKQIGNAVPVLLAEKIARPVMQFLENYK---------------------------------------------------
tr|B8DET5|B8DET5_LISMH Modific  QYKQIGNAVPVKLAFEMLLPIAKYMNEQKNRC------------------------------------------------
tr|B1HWG5|B1HWG5_LYSSC Modific  KYKQIGNAVPVLLAKEIAKPIAEFAKMHLQNKDEQEEPMLQLSLF-----------------------------------
tr|Q8GC38|Q8GC38_LEUCI Putativ  QYKQIGNAVPVQLARVVLQPIARFFVNNLSNL------------------------------------------------
tr|K4Z5B6|K4Z5B6_ENTFL DNAcyto  IYKQIGNAVPVLLAKAMTQPIADFISKNIAGIE-----------------------------------------------
tr|S3P180|S3P180_9GAMM Cytosin  KYKVVGNAVPPPLFEVIAKKLKNYF-------------------------------------------------------
tr|N9NU94|N9NU94_9GAMM Cytosin  KYKVVGNAVPPPLFEVIARTLRSLL-------------------------------------------------------
```

```
tr|E7GW62|E7GW62_STRAP Modific  IYKQVGNAVPVLLARAIIQPIADFLYSQYLK-------------------------------------------------
tr|I0QBF4|I0QBF4_STROR Modific  VYKQIGNAVPVLLARAIIQPIADFLNAHLEEIRKID--------------------------------------------
tr|S4B3K9|S4B3K9_ENTCA Modific  QYKQIGNAVPVYLGEKMVRPIIEFFHNHPELL------------------------------------------------
tr|S9C1L1|S9C1L1_STRAG Restric  VYKQIGNAVPVLLARAILQPIADFLNQNFAEIRNEE--------------------------------------------

                                        570       580       590       600       610       620       630       640
                                ....|....|....|....|....|....|....|....|....|....|....|....|....|....|....|....|
MHaeIII stabilized reference    --------------------------------------------------------------------------------
tr|A8SMR4|A8SMR4_9FIRM Cytosin  --------------------------------------------------------------------------------
tr|G9QUQ3|G9QUQ3_9PROT Cytosin  --------------------------------------------------------------------------------
tr|C7HVB2|C7HVB2_9FIRM Cytosin  --------------------------------------------------------------------------------
tr|D1AWH4|D1AWH4_STRM9 Cytosin  --------------------------------------------------------------------------------
sp|P34906|MTF1_FUSNU Modificat  --------------------------------------------------------------------------------
tr|R7P745|R7P745_9CLOT Cytosin  --------------------------------------------------------------------------------
tr|T4PMD0|T4PMD0_CLODI Modific  --------------------------------------------------------------------------------
tr|I7IJB3|I7IJB3_9BURK Cytosin  --------------------------------------------------------------------------------
tr|F8KQQ2|F8KQQ2_HELBC Cytosin  --------------------------------------------------------------------------------
tr|G4QBU1|G4QBU1_TAYAM Cytosin  --------------------------------------------------------------------------------
tr|B0S4D0|B0S4D0_FINM2 Cytosin  --------------------------------------------------------------------------------
tr|R5X970|R5X970_9FUSO Cytosin  --------------------------------------------------------------------------------
UPI000372CBB5 status=active     --------------------------------------------------------------------------------
tr|U2TAM1|U2TAM1_9FUSO Unchara  --------------------------------------------------------------------------------
UPI00016C0771 status=active     --------------------------------------------------------------------------------
tr|A8MHW3|A8MHW3_ALKOO Cytosin  --------------------------------------------------------------------------------
tr|G8X6Y8|G8X6Y8_FLACA Cytosin  --------------------------------------------------------------------------------
tr|I4AHT3|I4AHT3_FLELS Cytosin  --------------------------------------------------------------------------------
tr|K2Q409|K2Q409_9FLAO Cytosin  --------------------------------------------------------------------------------
UPI0003695DD5 status=active     --------------------------------------------------------------------------------
UPI0002E32502 status=active     --------------------------------------------------------------------------------
tr|A6EU94|A6EU94_9BACT Cytosin  --------------------------------------------------------------------------------
tr|E8KGI3|E8KGI3_9PAST Cytosin  --------------------------------------------------------------------------------
tr|N9DBX7|N9DBX7_9GAMM Cytosin  --------------------------------------------------------------------------------
tr|K0C3B4|K0C3B4_CYCSP Cytosin  --------------------------------------------------------------------------------
tr|F9GXX4|F9GXX4_HAEHA Cytosin  --------------------------------------------------------------------------------
tr|D1K024|D1K024_9BACE Modific  --------------------------------------------------------------------------------
UPI0002E6F434 status=active     --------------------------------------------------------------------------------
tr|D5ERM7|D5ERM7_PRER2 Cytosin  --------------------------------------------------------------------------------
UPI0003ABCEDB status=active     --------------------------------------------------------------------------------
tr|L2F549|L2F549_9GAMM Cytosin  --------------------------------------------------------------------------------
tr|R6WUU4|R6WUU4_9CLOT Cytosin  --------------------------------------------------------------------------------
tr|C9PPY5|C9PPY5_9PAST Cytosin  --------------------------------------------------------------------------------
tr|K5TXT5|K5TXT5_9VIBR Cytosin  --------------------------------------------------------------------------------
tr|R5VXV7|R5VXV7_9FIRM Cytosin  --------------------------------------------------------------------------------
tr|C2MAC7|C2MAC7_9PORP Cytosin  --------------------------------------------------------------------------------
tr|R7G2J8|R7G2J8_9FIRM Cytosin  --------------------------------------------------------------------------------
tr|F0QZK5|F0QZK5_BACSH DNAcyto  --------------------------------------------------------------------------------
sp|P08455|MTP2_NEIGO Modificat  --------------------------------------------------------------------------------
tr|A5FP46|A5FP46_DEHSB Cytosin  --------------------------------------------------------------------------------
tr|D4WI15|D4WI15_BACOV DNA (Cy  --------------------------------------------------------------------------------
tr|E2PDE9|E2PDE9_NEIPO Cytosin  --------------------------------------------------------------------------------
tr|D2EGA8|D2EGA8_9EURY DNAcyto  --------------------------------------------------------------------------------
tr|D7FEK9|D7FEK9_HELP3 Cytosin  --------------------------------------------------------------------------------
tr|D1B3R7|D1B3R7_SULD5 DNAcyto  --------------------------------------------------------------------------------
tr|D6ZKT7|D6ZKT7_MOBCV Cytosin  --------------------------------------------------------------------------------
tr|K2EZH0|K2EZH0_9BACT Cytosin  --------------------------------------------------------------------------------
tr|F0S2I5|F0S2I5_DESTD DNAcyto  --------------------------------------------------------------------------------
tr|R7K6Q8|R7K6Q8_9FIRM Cytosin  --------------------------------------------------------------------------------
tr|R5R432|R5R432_9FIRM Cytosin  --------------------------------------------------------------------------------
tr|D8G3S4|D8G3S4_9CYAN Modific  --------------------------------------------------------------------------------
tr|D3I3G0|D3I3G0_9BACT Modific  --------------------------------------------------------------------------------
tr|E3D818|E3D818_GARV3 Cytosin  --------------------------------------------------------------------------------
UPI0001D09BA1 status=active     --------------------------------------------------------------------------------
UPI00030E071A status=active     --------------------------------------------------------------------------------
tr|F1XAT6|F1XAT6_MORCA Cytosin  --------------------------------------------------------------------------------
tr|I3CCM6|I3CCM6_9GAMM DNAmeth  --------------------------------------------------------------------------------
tr|B6XDR0|B6XDR0_9ENTR Cytosin  --------------------------------------------------------------------------------
tr|K9YXG2|K9YXG2_DACSA DNAmeth  --------------------------------------------------------------------------------
tr|D4XUU8|D4XUU8_ACIHA Cytosin  --------------------------------------------------------------------------------
tr|N8YEA4|N8YEA4_ACIBZ Cytosin  --------------------------------------------------------------------------------
tr|B4VSA7|B4VSA7_9CYAN C5 cyto  --------------------------------------------------------------------------------
sp|P29567|MTHT_METTF Modificat  --------------------------------------------------------------------------------
tr|L8N028|L8N028_9CYAN DNAcyto  --------------------------------------------------------------------------------
tr|K0JMW8|K0JMW8_BRAPL Cytosin  --------------------------------------------------------------------------------
tr|K9XTJ8|K9XTJ8_STAC7 DNAcyto  --------------------------------------------------------------------------------
UPI00036A774A status=active     --------------------------------------------------------------------------------
UPI0003826E4D status=active     --------------------------------------------------------------------------------
UPI00037126EF status=active     --------------------------------------------------------------------------------
tr|K9TTW7|K9TTW7_9CYAN DNAcyto  --------------------------------------------------------------------------------
tr|K9RM80|K9RM80_9CYAN DNAmeth  --------------------------------------------------------------------------------
tr|R7CTJ4|R7CTJ4_9BACE Cytosin  --------------------------------------------------------------------------------
tr|D4WI14|D4WI14_BACOV Cytosin  LLLHNCSNRYLYAMVEDHPKVMSGSELSHLGFAPSGNEYLTFKLKTAECINLECLNLADVKFRGNKRDIAIPYIANIQEL
tr|F0QZK6|F0QZK6_BACSH Cytosin  LLLHHKKSRFLYKLIPKSPSCVTAADLSAKGFSPSGNEYLTFGLENTKEIHIKDLNLQTIQLP-NGRNATFPYITDIETL
tr|D1K023|D1K023_9BACE Cytosin  LLLHNHNNKFLFRIIPDYPKLISASDLIKLGFMPSGKEYFAFRLESAQSINIVGVDLSKVQIKGKNHNKAIPYITPIQDF
UPI00037FAC0D status=active     LLLHNRSNKFLFRIIPEYPKLMSASGLKKLGFMPSGKEYFAFRLESPLSTNLAGMDLSKVRIKGKNRNIAIPYITTIQEL
tr|R9HQV7|R9HQV7_BACVU Cytosin  LLLHNHNNRFLFRIIPEYPKLMSTSDLIELGFTPSGKEYFAFRLESTQNINLAGMDLSKLQIKGRSHNIAIPYISDIQEI
UPI0003717B77 status=active     --------------------------------------------------------------------------------
tr|K9VHM5|K9VHM5_9CYAN DNAcyto  --------------------------------------------------------------------------------
tr|T4JUF1|T4JUF1_CLODI Modific  --------------------------------------------------------------------------------
tr|S8B9I9|S8B9I9_CLOBO Cytosin  --------------------------------------------------------------------------------
tr|J0QZQ6|J0QZQ6_9RHIZ DNA (Cy  --------------------------------------------------------------------------------
tr|R5PBE9|R5PBE9_9BACT Cytosin  --------------------------------------------------------------------------------
tr|G6A2H7|G6A2H7_STRIT Cytosin  --------------------------------------------------------------------------------
tr|Q9K765|Q9K765_BACHD Cytosin  --------------------------------------------------------------------------------
tr|H1B1E9|H1B1E9_9FIRM Cytosin  --------------------------------------------------------------------------------
tr|A3DHU3|A3DHU3_CLOTH Cytosin  --------------------------------------------------------------------------------
```

```
tr|K4ZRS7|K4ZRS7_PAEAL Cytosin  --------------------------------------------------------------------------------
tr|F4PEX7|F4PEX7_BATDJ Cytosin  --------------------------------------------------------------------------------
tr|E5YPJ2|E5YPJ2_9BACL Cytosin  VYRRGQSQTLVMNRKNIVYRRRKSRSK-----------------------------------------------------
sp|P13906|MTB1_LYSSH Modificat  --------------------------------------------------------------------------------
tr|J7X9T9|J7X9T9_BACCE Cytosin  --------------------------------------------------------------------------------
tr|C7G547|C7G547_9FIRM Cytosin  --------------------------------------------------------------------------------
sp|P06530|MTBR_BACIU Modificat  --------------------------------------------------------------------------------
tr|F0TJ68|F0TJ68_LACA3 Cytosin  --------------------------------------------------------------------------------
tr|B8DET5|B8DET5_LISMH Modific  --------------------------------------------------------------------------------
tr|B1HWG5|B1HWG5_LYSSC Modific  --------------------------------------------------------------------------------
tr|Q8GC38|Q8GC38_LEUCI Putativ  --------------------------------------------------------------------------------
tr|K4Z5B6|K4Z5B6_ENTFL DNAcyto  --------------------------------------------------------------------------------
tr|S3P180|S3P180_9GAMM Cytosin  --------------------------------------------------------------------------------
tr|N9NU94|N9NU94_9GAMM Cytosin  --------------------------------------------------------------------------------
tr|E7GW62|E7GW62_STRAP Modific  --------------------------------------------------------------------------------
tr|I0QBF4|I0QBF4_STROR Modific  --------------------------------------------------------------------------------
tr|S4B3K9|S4B3K9_ENTCA Modific  --------------------------------------------------------------------------------
tr|S9C1L1|S9C1L1_STRAG Restric  --------------------------------------------------------------------------------


                                ....|.
MHaeIII stabilized reference    ------
tr|A8SMR4|A8SMR4_9FIRM Cytosin  ------
tr|G9QUQ3|G9QUQ3_9PROT Cytosin  ------
tr|C7HVB2|C7HVB2_9FIRM Cytosin  ------
tr|D1AWH4|D1AWH4_STRM9 Cytosin  ------
sp|P34906|MTF1_FUSNU Modificat  ------
tr|R7P745|R7P745_9CLOT Cytosin  ------
tr|T4PMD0|T4PMD0_CLODI Modific  ------
tr|I7IJB3|I7IJB3_9BURK Cytosin  ------
tr|F8KQQ2|F8KQQ2_HELBC Cytosin  ------
tr|G4QBU1|G4QBU1_TAYAM Cytosin  ------
tr|B0S4D0|B0S4D0_FINM2 Cytosin  ------
tr|R5X970|R5X970_9FUSO Cytosin  ------
UPI000372CBB5 status=active     ------
tr|U2TAM1|U2TAM1_9FUSO Unchara  ------
UPI00016C0771 status=active     ------
tr|A8MHW3|A8MHW3_ALKOO Cytosin  ------
tr|G8X6Y8|G8X6Y8_FLACA Cytosin  ------
tr|I4AHT3|I4AHT3_FLELS Cytosin  ------
tr|K2Q409|K2Q409_9FLAO Cytosin  ------
UPI0003695DD5 status=active     ------
UPI0002E32502 status=active     ------
tr|A6EU94|A6EU94_9BACT Cytosin  ------
tr|E8KGI3|E8KGI3_9PAST Cytosin  ------
tr|N9DBX7|N9DBX7_9GAMM Cytosin  ------
tr|K0C3B4|K0C3B4_CYCSP Cytosin  ------
tr|F9GXX4|F9GXX4_HAEHA Cytosin  ------
tr|D1K024|D1K024_9BACE Modific  ------
UPI0002E6F434 status=active     ------
tr|D5ERM7|D5ERM7_PRER2 Cytosin  ------
UPI0003ABCEDB status=active     ------
tr|L2F549|L2F549_9GAMM Cytosin  ------
tr|R6WUU4|R6WUU4_9CLOT Cytosin  ------
tr|C9PPY5|C9PPY5_9PAST Cytosin  ------
tr|K5TXT5|K5TXT5_9VIBR Cytosin  ------
tr|R5VXV7|R5VXV7_9FIRM Cytosin  ------
tr|C2MAC7|C2MAC7_9PORP Cytosin  ------
tr|R7G2J8|R7G2J8_9FIRM Cytosin  ------
tr|F0QZK5|F0QZK5_BACSH DNAcyto  ------
sp|P08455|MTP2_NEIGO Modificat  ------
tr|A5FP46|A5FP46_DEHSB Cytosin  ------
tr|D4WI15|D4WI15_BACOV DNA (Cy  ------
tr|E2PDE9|E2PDE9_NEIPO Cytosin  ------
tr|D2EGA8|D2EGA8_9EURY DNAcyto  ------
tr|D7FEK9|D7FEK9_HELP3 Cytosin  ------
tr|D1B3R7|D1B3R7_SULD5 DNAcyto  ------
tr|D6ZKT7|D6ZKT7_MOBCV Cytosin  ------
tr|K2EZH0|K2EZH0_9BACT Cytosin  ------
tr|F0S2I5|F0S2I5_DESTD DNAcyto  ------
tr|R7K6Q8|R7K6Q8_9FIRM Cytosin  ------
tr|R5R432|R5R432_9FIRM Cytosin  ------
tr|D8G3S4|D8G3S4_9CYAN Modific  ------
tr|D3I3G0|D3I3G0_9BACT Modific  ------
tr|E3D818|E3D818_GARV3 Cytosin  ------
UPI0001D09BA1 status=active     ------
UPI00030E071A status=active     ------
tr|F1XAT6|F1XAT6_MORCA Cytosin  ------
tr|I3CCM6|I3CCM6_9GAMM DNAmeth  ------
tr|B6XDR0|B6XDR0_9ENTR Cytosin  ------
tr|K9YXG2|K9YXG2_DACSA DNAmeth  ------
tr|D4XUU8|D4XUU8_ACIHA Cytosin  ------
tr|N8YEA4|N8YEA4_ACIBZ Cytosin  ------
tr|B4VSA7|B4VSA7_9CYAN C5 cyto  ------
sp|P29567|MTHT_METTF Modificat  ------
tr|L8N028|L8N028_9CYAN DNAcyto  ------
tr|K0JMW8|K0JMW8_BRAPL Cytosin  ------
tr|K9XTJ8|K9XTJ8_STAC7 DNAcyto  ------
UPI00036A774A status=active     ------
UPI0003826E4D status=active     ------
UPI00037126EF status=active     ------
tr|K9TTW7|K9TTW7_9CYAN DNAcyto  ------
tr|K9RM80|K9RM80_9CYAN DNAmeth  ------
tr|R7CTJ4|R7CTJ4_9BACE Cytosin  ------
tr|D4WI14|D4WI14_BACOV Cytosin  F-----
```

```
tr|F0QZK6|F0QZK6_BACSH Cytosin  RKELK-
tr|D1K023|D1K023_9BACE Cytosin  IYRINA
UPI00037FAC0D status=active     ISE---
tr|R9HQV7|R9HQV7_BACVU Cytosin  IIK---
UPI0003717B77 status=active     ------
tr|K9VHM5|K9VHM5_9CYAN DNAcyto  ------
tr|T4JUF1|T4JUF1_CLODI Modific  ------
tr|S8B9I9|S8B9I9_CLOBO Cytosin  ------
tr|J0QZQ6|J0QZQ6_9RHIZ DNA (Cy  ------
tr|R5PBE9|R5PBE9_9BACT Cytosin  ------
tr|G6A2H7|G6A2H7_STRIT Cytosin  ------
tr|Q9K765|Q9K765_BACHD Cytosin  ------
tr|H1B1E9|H1B1E9_9FIRM Cytosin  ------
tr|A3DHU3|A3DHU3_CLOTH Cytosin  ------
tr|K4ZRS7|K4ZRS7_PAEAL Cytosin  ------
tr|F4PEX7|F4PEX7_BATDJ Cytosin  ------
tr|E5YPJ2|E5YPJ2_9BACL Cytosin  ------
sp|P13906|MTB1_LYSSH Modificat  ------
tr|J7X9T9|J7X9T9_BACCE Cytosin  ------
tr|C7G547|C7G547_9FIRM Cytosin  ------
sp|P06530|MTBR_BACIU Modificat  ------
tr|F0TJ68|F0TJ68_LACA3 Cytosin  ------
tr|B8DET5|B8DET5_LISMH Modific  ------
tr|B1HWG5|B1HWG5_LYSSC Modific  ------
tr|Q8GC38|Q8GC38_LEUCI Putativ  ------
tr|K4Z5B6|K4Z5B6_ENTFL DNAcyto  ------
tr|S3P180|S3P180_9GAMM Cytosin  ------
tr|N9NU94|N9NU94_9GAMM Cytosin  ------
tr|E7GW62|E7GW62_STRAP Modific  ------
tr|I0QBF4|I0QBF4_STROR Modific  ------
tr|S4B3K9|S4B3K9_ENTCA Modific  ------
tr|S9C1L1|S9C1L1_STRAG Restric  ------
```
