# Supplementary material for: Systematic Mapping of Protein Mutational Space by Prolonged Drift Reveals the Deleterious Effects of Seemingly Neutral Mutations
Source: PLoS Comput Biol. 2015 Aug 14;11(8):e1004421. doi: 10.1371/journal.pcbi.1004421 (PMC4537296; doi:10.1371/journal.pcbi.1004421)
Supplement: S1 Table — (PDF) [file pcbi.1004421.s012.pdf]

		Average	SD	By position in codon		
				1st	2nd	3rd
**Transitions**	C->T	0.22%	0.10%	0.22%	0.22%	0.22%
	G->A	0.21%	0.10%	0.21%	0.23%	0.20%
	A->G	0.19%	0.06%	0.17%	0.19%	0.20%
	T->C	0.18%	0.06%	0.17%	0.19%	0.17%
**Fraction (Transitions)**		0.56				
**Transversion**	T->A	0.15%	0.07%	0.14%	0.17%	0.14%
	A->T	0.15%	0.07%	0.14%	0.16%	0.15%
	G->T	0.10%	0.06%	0.10%	0.09%	0.10%
	C->A	0.10%	0.07%	0.10%	0.11%	0.09%
	C->G	0.03%	0.03%	0.04%	0.04%	0.03%
	T->G	0.03%	0.04%	0.04%	0.04%	0.03%
	G->C	0.03%	0.03%	0.03%	0.04%	0.03%
	A->C	0.03%	0.03%	0.03%	0.03%	0.03%
**Fraction (Transversion)**		0.44				
**Transitions/Transversion**		1.28				
