# Supplementary material for: Systematic Mapping of Protein Mutational Space by Prolonged Drift Reveals the Deleterious Effects of Seemingly Neutral Mutations
Source: PLoS Comput Biol. 2015 Aug 14;11(8):e1004421. doi: 10.1371/journal.pcbi.1004421 (PMC4537296; doi:10.1371/journal.pcbi.1004421)
Supplement: S2 Table — Compensatory mutations were defined as enriched mutations, either by assigned beneficial fitness effect for individual mutations by (W rel >1.1) or high positional fitness effect (the averaged W rel per position as calculated in Fig 3A, W rel (Positional) >1.1). Shown are the W rel of mutations that were enriched in the selected G17 library. Also noted are the sequence divergence and the frequency of these exchanges in the natural diversity relative to M.HaeIII, and the frequency of the mutation under the selection for new functions [51]. (PDF) [file pcbi.1004421.s013.pdf]

Mutation	Positional W_{rel}(G17)	W_{rel}(G17)	Orthologs		New Functions
			Frequency	Divergence	
N2D	1.30	1.02	4%	0.29	
N2S		1.11	5%	0.45	
A26G	1.52	0.95			
A26T		0.98	4%	0.26	32%
A26V		1.07	19%	0.29	3%
P86A	1.50	1.11	18%	0.33	
P86S		1.09	10%	0.33	
K102E	1.43	0.95			
K102I		1.15			
K102Q		1.07	57%	0.25	
K102T		1.01	2%	0.36	
K104I	1.16	0.99	1%	0.60	
K104N		0.97	1%	0.45	
K104Q		0.94	1%	0.47	
K104R		1.06	4%	0.30	
N120H	3.17	1.13	1%	0.45	
N120I		0.96	3%	0.48	22%
N120K		0.99	13%	0.32	96%
N120S		1.10	18%	0.36	
N120T		1.19	6%	0.30	3%
N120Y		0.97			
E125D	1.18	0.93	4%	0.30	4%
E125G		1.11	8%	0.32	4%
Q128R	1.54	1.10	2%	0.37	3%
D131A	1.52	1.01	7%	0.33	
D131H		1.00			
D131V		1.12			36%
I139F	1.97	0.95	16%	0.32	6%
I139M		1.07	2%	0.36	
I139V		1.17	5%	0.36	18%
I140M	2.75	1.14	1%	0.52	
I140N		1.09	5%	0.30	
I140S		1.12	7%	0.38	
I140V		0.98			
N168K	1.10	1.05	26%	0.28	80%
I173L	1.26	0.98	5%	0.33	
I173M		0.95			
I173T		0.93	10%	0.33	
I173V		1.04	3%	0.40	

L176S	1.12	1.02	7%	0.26	7%
L176V		1.05	1%	0.44	
N191S	1.47	1.11	21%	0.26	7%
N191T		0.96	30%	0.26	
P192A	0.69	1.12	54%	0.25	7%
T220S	1.79	1.14	31%	0.46	14%
Q244H	1.98	1.12	34%	0.46	100%
N262H	4.72	1.28	3%	0.29	4%
N262S		1.03			
N262T		0.98			
N262Y		1.36	11%	0.30	100%
S296C	1.40	0.93			
S296N		1.02	33%	0.26	93%
S296R		1.02	13%	0.29	
E325D	1.97	1.13	5%	0.30	3%
E325G		0.92	1%	0.64	
E325K		0.98	13%	0.30	
I326F	1.45	0.98	2%	0.46	
I326L		0.93	2%	0.44	
I326N		1.01	14%	0.25	
I326S		0.93	12%	0.29	
I326T		0.91	4%	0.29	
I326V		0.99	1%	0.36	
