# Supplementary material for: Systematic Mapping of Protein Mutational Space by Prolonged Drift Reveals the Deleterious Effects of Seemingly Neutral Mutations
Source: PLoS Comput Biol. 2015 Aug 14;11(8):e1004421. doi: 10.1371/journal.pcbi.1004421 (PMC4537296; doi:10.1371/journal.pcbi.1004421)
Supplement: S3 Table — ‘Sensitivity (TPR)’–True positives rate; correctly identified as deleterious mutations, TP, out of the total deleterious mutations. ‘Specificity (TNR)’- True negatives rate; correctly identified as neutral mutations, TN, out of the total neutral mutations. ‘FPR’–false positive rate; incorrectly identified as deleterious mutations, FP, and are in fact neutral out of the total neural mutations. ‘FNR’—false negative rate; incorrectly identified as neutral mutations, FN, and are in fact deleterious out of the total neural mutations. Accuracy = (TP + TN)/(TP + TN + FP + FN) (PDF) [file pcbi.1004421.s014.pdf]

Functional outcome			Prediction tool	Score threshold	Performance				
Deleterious	Neutral	Total			Sensitivity (TPR)	Specificity (TNR)	Accuracy (ACC)	FPR	FNR
$W_{rel} \leq 0.6$ 1,234	$W_{rel} > 0.6$ 723	1,957	PROVEAN	-2.5	83.3%	63.5%	**76.0%**	36.5%	16.7%
			SIFT	0.05	73.0%	77.0%	**74.5%**	23.0%	27.0%
$W_{rel} \leq 0.6$ 1,234	$W_{rel} > 0.8$ 415	1,649	PROVEAN	-2.5	83.3%	72.0%	**80.5%**	28.0%	16.7%
			SIFT	0.05	73.0%	82.2%	**75.3%**	17.8%	27.0%
