# Supplementary material for: Systematic Mapping of Protein Mutational Space by Prolonged Drift Reveals the Deleterious Effects of Seemingly Neutral Mutations
Source: PLoS Comput Biol. 2015 Aug 14;11(8):e1004421. doi: 10.1371/journal.pcbi.1004421 (PMC4537296; doi:10.1371/journal.pcbi.1004421)
Supplement: S4 Table — 'Threshold'—refers to the background frequencies observed in each library at the unmutated region. "≤ Threshold"—refers to the number of codons in the mutated M.HaeIII's ORF in each library with frequencies below the threshold that were excluded from the analysis. "> Threshold"—refers to the number of codons in the mutated M.HaeIII's ORF in each library with frequencies above the threshold, and thus were included in the analysis. (PDF) [file pcbi.1004421.s015.pdf]

| | | |
|---|---|---|
| G0 | ≤ Threshold | 117 |
| | Threshold | 0.030% |
| | **> Threshold** | **2,844** |
| G3 | ≤ Threshold | 683 |
| | Threshold | 0.025% |
| | **> Threshold** | **2,278** |
| G7 | ≤ Threshold | 875 |
| | Threshold | 0.025% |
| | **> Threshold** | **2,086** |
| G17 | ≤ Threshold | 842 |
| | Threshold | 0.027% |
| | **> Threshold** | **2,118** |
| Total | **Original** | 2,961 |
| | **>Threshold** | **2,907** |
